# Use of Endogenous Retroviral Sequences (ERVs) and structural markers for retroviral phylogenetic inference and taxonomy

**DOI:** 10.1186/1742-4690-2-50

**Published:** 2005-08-10

**Authors:** Patric Jern, Göran O Sperber, Jonas Blomberg

**Affiliations:** 1 Section of Virology, Department of Medical Sciences, Uppsala University, Uppsala, Sweden; 2 Unit of Physiology, Department of Neuroscience, Uppsala University, Uppsala, Sweden

## Abstract

**Background:**

Endogenous retroviral sequences (ERVs) are integral parts of most eukaryotic genomes and vastly outnumber exogenous retroviruses (XRVs). ERVs with a relatively complete structure were retrieved from the genetic archives of humans and chickens, diametrically opposite representatives of vertebrate retroviruses (over 3300 proviruses), and analyzed, using a bioinformatic program, RetroTector^©^, developed by us. This rich source of proviral information, accumulated in a local database, and a collection of XRV sequences from the literature, allowed the reconstruction of a Pol based phylogenetic tree, more extensive than previously possible. The aim was to find traits useful for classification and evolutionary studies of retroviruses. Some of these traits have been used by others, but they are here tested in a wider context than before.

**Results:**

In the ERV collection we found sequences similar to the XRV-based genera: alpha-, beta-, gamma-, epsilon- and spumaretroviruses. However, the occurrence of intermediates between them indicated an evolutionary continuum and suggested that taxonomic changes eventually will be necessary. No delta or lentivirus representatives were found among ERVs. Classification based on Pol similarity is congruent with a number of structural traits. Acquisition of dUTPase occurred three times in retroviral evolution. Loss of one or two NC zinc fingers appears to have occurred several times during evolution. Nucleotide biases have been described earlier for lenti-, delta- and betaretroviruses and were here confirmed in a larger context.

**Conclusion:**

Pol similarities and other structural traits contribute to a better understanding of retroviral phylogeny. "Global" genomic properties useful in phylogenies are **i.) **translational strategy, **ii.) **number of Gag NC zinc finger motifs, **iii.) **presence of Pro N-terminal dUTPase (dUTPase^Pro^), **iv.) **presence of Pro C-terminal G-patch and **v.) **presence of a GPY/F motif in the Pol integrase (IN) C-terminal domain. "Local" retroviral genomic properties useful for delineation of lower level taxa are **i.) **host species range, **ii.) **nucleotide compositional bias and **iii.) **LTR lengths.

## Background

Retroviral and related endogenous retroviral sequences (ERVs) are integral parts of most eukaryotic genomes, sometimes constituting over 50% of them [1]. Their ability to transpose and transfer horizontally [2,3], confers genetic flexibility to complex genomes like those of humans [4], chimpanzees [5], other primates and vertebrates.

The origin of retroviruses is lost in a prebiotic mist. Assuming a 0.2% neutral substitution rate per million years [6] and a 50% divergence limit for nucleotide sequence recognition, retroviral sequences >250 Million years old cannot be found in current genomes. If any of their genes are selected for, they may stay recognizable longer. Thus, although the ERV record has limitations, the reconstruction of retrovirus evolution differs fundamentally from that of other viruses, due to the ERVs in the ever richer archive of genomic assemblies. According to the VIIth ICTV report [7], *Retroviridae *borders to *Pararetroviridae *(e.g. Hepatitis B), *Metaviridae *(Gypsy-like) and *Pseudoviridae *(Copia-like). Together with the even more more distant relatives Mal-R [8], DIRS [9] retrotransposons and chromoviruses [10], not included here, they show that retroviruses are parts of a vast retrotransposon sequence universe. In this work, we concentrated on retroviruses. An ancestral retrovirus likely had structural traits which at present are common denominators of the diverse related sequences. Although some structural traits may be absent in individual viruses, readily identifiable common denominators are 5'LTR, PBS, Gag (MA, CA and NC), Pro, Pol, Env, PPT and 3'LTR [11]. The most universal trait is the *pol *gene, with its reverse transcriptase (RT), RNAse H and integrase (IN). The use of other conserved but distinguishing traits in phylogenetic inference and retroviral classification discussed here are: nucleotide bias, number of zinc fingers, translational strategy, C-terminal Pro and Pol motifs, presence of dUTPase and accessory genes and LTR length. Env is an unreliable evolutionary marker, exemplified by the hybrid betaretroviral MPMV [11], but can be useful in narrow phylogenies to demarcate a specific group.

Retroviral taxonomy has traditionally been based on observed phenotypic qualities of exogenous retroviruses (XRVs) [7]. Classification using ERVs, with an almost complete lack of phenotypic information, necessitates a nucleotide sequence analytical approach. Seven retroviral genera have been described (alpha-, beta-, gamma-, delta-, epsilon-, lenti- and spuma-like retroviruses) using sequence similarities, mainly in the Pol RT region. Although much work remains before all ERVs are fully characterized, ERVs have also been divided into loosely defined classes, originally based on HERVs [12-14]. When analyzing the RT region, the gammaretroviruses cluster as class I and betaretroviruses as class II elements [12]. The spuma- and spumalike elements group within the class III [14]. Lenti- and deltaretroviruses have no known endogenous counterparts [15]. This was also the case in our computerized genomewide screenings (see below).

ERV classification and grouping originally was based on sequence similarity between the proviral PBS and the host tRNA [11]. This classification has proved useful for some ERVs, e.g. HERV-E [16] and mostly for HERV-H [17]. However, it is inconsistent for many other ERV groups that have alternative PBSes [18] e.g. HERV-H/F [17], ERV3 [16], and ERV9/HERV-W [19]. We did not extend these analyses here.

In several papers [[17,20] and Jern et al. *submitted*], we have used Pol similarity for ERV classification. Pol is highly conserved, and its large size (800–1100 aa) provides adequate information for a relatively detailed classification. This is facilitated by the program RetroTector^© ^[Sperber G.O. et al. *in preparation*], which reconstructs probable Pol proteins ("puteins") from different reading frames in the often damaged gene candidates. The puteins are favored over nucleotide sequences since they are more conserved, easier to align and therefore allow phylogenetic inference and taxonomy over greater evolutionary distances. This is further discussed in the Methods and Results sections of this paper. A number of reliable distinguishing features must be defined to enable a durable retroviral taxonomy which can encompass the many new ERVs and XRVs, and to trace their evolution. In this study, we compared phylogenetic trees, based on Pol similarity, with distinct structural features of possible use as taxonomic and phylogenetic markers.

## Results and Discussion

### Genomic ERV collection

Using the program RetroTector^© ^(see methods), we screened the human hg16 [4] and chicken gg01 [21] genomes for ERVs. We found them to encompass 3149 and 260 proviral sequences with a RetroTector^© ^score of more than 300, respectively. A detailed account will be published separately [Blomberg J. et al. *in preparation*]. Based on experience from randomized data set scores (data not shown), this threshold separated false from true retroviral elements with a wide margin. We collected the sequences into an ERV databank, from which we extracted representative sequences for use in matching structural traits against sequence similarity based phylogenetic inference. Sequences scoring over 300 from the hg16 and gg01 genomes were analyzed for the presence of Pol. Those with a recognizable Pol were grouped into respective genera according to sequence similarity (Table 1). ERVs were found in all retroviral genera, except lenti- and deltaretroviruses. Our bioinformatic screening of a larger dataset thus confirmed the results of Herniou et al. [15]. As genomic assemblies from more species become available, analysis of upcoming retroviral sequences will increase the precision of phylogenetic inference and retroviral taxonomy.

**Table 1:** Detected ERV structural traits in the human and chicken genomes

**Genome(s)**^1^	**Genus**	**Class**	**ERVs**^2^	**Gag-Pro f.s.**^3^			**Pro-Pol f.s.**^3^			**dUTPase**^4^	**C-term. motifs**^5^	
		*(ERV)*		-*1*	*0*	*1*	-*1*	*0*	*1*	*(dUTPase*^Pro^*)*	*G-patch*	*GPY/F*
gg01	alpha		34	**4**	1	1	**10**	2	1	0	0	0
gg01	alpha-beta		67	**5**	2	0	**5**	3	1	21	0	0
gg01 and hg16	beta	II	582	**49**	18	14	**50**	22	27	363	68	0
gg01 and hg16	gamma	I	2069	14	**55**	13	32	**64**	43	0	0	264
gg01 and hg16	delta		(-)^6^	(-)	(-)	(-)	(-)	(-)	(-)	(-)	(-)	(-)
gg01 and hg16	epsilon		n.d.^7^	n.d.	n.d.	n.d.	n.d.	n.d.	n.d.	n.d.	n.d.	n.d.
gg01 and hg16	lenti		(-)	(-)	(-)	(-)	(-)	(-)	(-)	(-)	(-)	(-)
gg01 and hg16	spuma-like	III	193	n.d.	n.d.	n.d.	n.d.	n.d.	n.d.	n.d.	n.d.	n.d.

^1 ^Human genome version 16 (hg16) and Chicken genome (gg01). Alpha and alpha-beta retroviruses were only detected in the chicken genome

^2 ^Detected ERVs from the databank collection, with RetroTector^© ^score >300 and recognized Pol puteins.

^3 ^Predicted frameshift (f.s.) translation strategy between the respective putein ORFs in elements with score >1000, and no f.s. in the near 3'-end.

^4 ^Detected dUTPase N-terminal of protease domain (dUTPase^Pro^) in elements with score >300.

^5 ^Detected C-terminal Pro (G-patch) and Pol (GPY/F) motifs elements with score >300.

^6 ^(-): no delta or lentiviral ERVs were detected in the genomes.

^7 ^(n.d.): not determined. The scarcity of epsilon like elements [19] and the highly mutated nature of both epsilon and spuma-like elements, in the human genome did not provide sufficient information to conduct a thorough analysis.

### Phylogenetic reconstruction based on Pol

**Figure 1:**
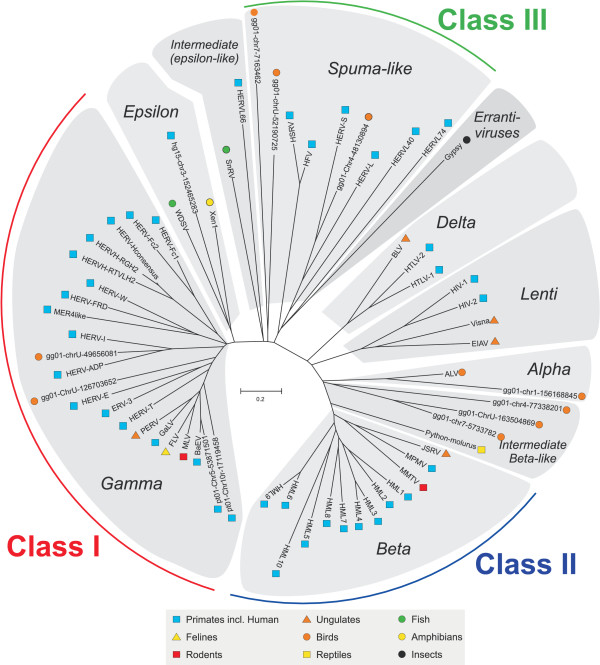
**Representative unrooted Pol neighbor joining (NJ) dendrogram. **Unrooted Pol neighbor joining (NJ) dendrogram (500 bootstraps consensus) of the seven retroviral genera: alpha-, beta-, gamma-, delta-, epsilon-, lenti- and spuma-like retroviruses. The somewhat more loosely defined (endogenous) retroviral classes are indicated in the periphery. The various host species are indicated with symbols next to each taxonomic unit. The novel sequences are named according to their chromosomal positions within respective genomes. (hg15 and 16: Human genome; gg01: Chicken genome and pt01: chimpanzee genome). The two pt01 sequences were unique to chimpanzee and not found in humans [Jern et al. *submitted*].

Using the whole Pol proteins/puteins retrieved from the genetic archive, we reconstructed an unrooted retroviral neighbor joining (NJ) tree. We used the whole Pol and the principle of pairwise deletions in the alignment and distance matrix analyses to avoid problems with missing portions in RT as in e.g. the large HERV-H group [17]. To reconstruct a comprehensible condensed phylogeny, we chose to include only 12 representative ERVs from the human hg16 [4], chimpanzee pt01 [5] (collected and analyzed earlier [Jern et al. *submitted*]) and chicken gg01 [21] genome assemblies. The human and chicken sequences were chosen because they are diametrically opposite representatives of vertebrate retroviruses (including over 3300 proviruses). The representative ERVs from chimpanzee (one BaEV like and one PTERV1 like [3], not found in humans [Jern et al. *submitted*]) were included to provide a broad based phylogeny. The remaining representative ERVs, all with high RetroTector^© ^scores, were selected to contribute with different aspects, e.g. intermediate positions in the tree, while still keeping the size of the Pol tree manageable. Annotated exogenous retroviruses retrieved from GenBank were added to form a tree backbone structure useful for taxonomic reference (Figure 1). The Pol NJ (500 bootstrap consensus) tree structure was confirmed using an array of maximum likelihood (ML) analyses (data not shown). The wealth of mutated ERV sequences sometimes makes delineation of genera and groups difficult. We have earlier used the Pol similarity (>80%) based clustering as a primary criterion for retroviral groups [16,17]. This corresponds to the finer branches of the genera (Figure 1), which themselves tend to have internal Pol similarities over 60% (Additional file 1). The phylogenetic Pol tree shows the seven retroviral genera, defined from clustering of ERVs next to the earlier classified XRVs (see bootstrap supports in NJ tree, additional file 1), and the three loosely defined ERV classes [12-14] (Figure 1). Further, the tree shows two major branches, ending in gamma- and betaretroviruses, respectively. They consistently have very high bootstrap supports (Additional file 1). The continuous influx of new data will eventually necessitate a revision of the retroviral genera. This was out of scope for the present study. An especially amorphous part of the tree is its center. In numerous phylogenetic analyses with a sequence set (not shown here), we found that the spuma-like group referred to here, includes both the exogenous spumaviruses and a diverse group of related endogenous retroviral sequences (primarily ERV-L). These and other centrally located elements often are highly mutated and difficult to analyze. Further, the tree (Figure 1) shows ERV and XRV sequences intermediate between the major genera. In the left major branch (the main "gamma" branch), Snakehead retrovirus (SnRV) is intermediate between epsilon and spumalike retroviruses. In the main "beta-branch", several chicken ERVs and the reptilian Python RV [22] are intermediate between the previously recognized delta, lenti, alpha and betaretroviruses, supporting a gradual evolution of betaretroviruses from delta/lenti and alpharetrovirus-like ancestors.

### Host species

Although host species is not a structural feature, it is an easily definable trait, and is therefore discussed here. Retroviral classification using host species is at first sight appealing: Classical gammaretroviruses are murine, epsilon piscine, alpha avian and beta mammalian. However, as seen in figure 1, this order is not maintained when additional XRVs and ERVs are included. It has been shown that some avian retroviruses share similarity with human gammaretroviral (class I) HERV-I elements [23], and probably are the results of horizontal transfers [15]. In our screening, we confirmed these avian HERV-I like elements and also show a novel avian sequence extracted from the chicken genome that is similar to HERV-E (Figure 1). Further, it has been shown that piscine elements grouped together with some human elements [15]. In a recent bioinformatic study, we found human epsilon-like proviral elements [19]. One of them was included into the phylogeny (Figure 1). Transspecies transfers between vertebrates have been discussed repeatedly [15,22,23]. Indeed, the genomes of the two vertebrate species used here encompass ERVs clustering with five retroviral genera, indicating widespread cross-species transmissions (Figure 1). Several such horizontal transmission events have been described for gammaretroviruses [[3] and Jern et al. *submitted*] and lentiviruses [2]. Although co-evolution with the host (vertical transmission) is the dominant mode of retroviral transmission, occasional horizontal transmissions make the host species an often unreliable taxonomic marker.

### Gag zinc fingers

**Figure 2:**
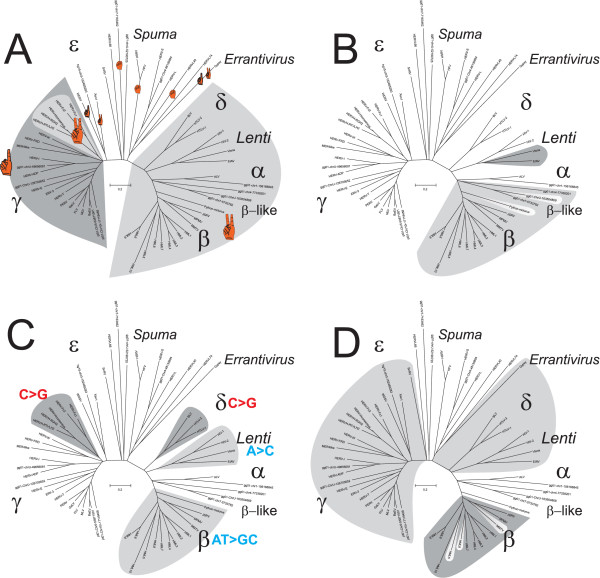
**Structural traits projected onto the Pol dendrogram. **The pol dendrograms in panels A to D are all derived from figure 1. **A**. The number of recognized Gag NC zinc finger motifs within respective genera. Detections of two NC zinc fingers are marked in light grey for all genera to the right from deltaretroviruses to betaretroviruses, and also for the gammaretroviral HERV-H. The remaining gammaretroviral elements (dark grey) had one NC zinc finger **B. **Presence of dUTPase is highlighted in grey. The non-primate lentiviral dUTPase^PolA ^(dark grey) is found within Pol and the dUTPase^Pro ^(light grey) are found N-terminal of Pro. The dUTPase^Pro ^appears to occasionally have been lost, indicated by the two uncolored intermediate chicken and python ERVs. dUTPase^PolB^. of foamy viruses is not indicated. **C. **Nucleotide biases may be useful in demarcating retroviral groups locally and the most obvious found are here highlighted. For more detail see refs [31, 40]. **D**. Genera with detected Pol C-terminal GPY/F motifs are marked light grey and Pro C-terminal G-patch marked in dark grey (exclusively in betaretroviruses). Some betaretroviruses missed a G-Patch and are therefore unmarked.

In addition to Pol, the Gag is also suitable for structural analysis. It is relatively conserved and has well documented functional domains for retroviral RNA packaging, assembly and budding [24-30]. Analysis of the nucleocapsid (NC) from the different genera showed a difference in number of zinc finger motifs, involved in the retroviral RNA interaction [26,28]. Two zinc fingers were detectable in lenti-, alpha-, beta-, epsilon- and some gammaretroviruses (the HERV-H group), whereas the remaining gammaretroviruses had only one, and the spuma-like HERV-L and spumaviruses themselves had none (Figure 2A). The gammaretroviral MLV has a charged amino acid segment upstream of the zinc finger. Recently, we demonstrated that this feature appears to gradually have replaced the loss of the second NC zinc finger in the MLV like group [31]. In the extended data set used here, we could also see that the intermediate SnRV has only one zinc finger (Additional file 2), an indication of several zinc finger loss events. Spumaretroviruses and their related sequences, present in vertebrates and reptiles [15], stand out as structurally different. They have no zinc fingers. They have a separately spliced *pro *ORF and a relatively low Pol similarity (47.1–61.8%) to other retroviruses. Because most other retroviruses and related viruses (Gypsy and Copia) have NC zinc fingers, it is likely that the spuma-like elements lost theirs. The sequences of the main "beta" branch all have two NC zinc fingers. Aside from this "global" aspect, the uneven distribution and various numbers of NC zinc fingers in the comprehensive sequence collection (Figure 2A), makes the zinc finger trait useful for group delineation rather than for general taxonomy.

### Translational strategy

In order to produce differing amounts of the different retroviral proteins, the retrovirus may either use **i.) **ribosomal frameshifting, **ii.) **nonsense codon readthrough or **iii.) **splicing, as translational strategies. Well studied gamma and epsilonretroviruses have a distinct genomic structure where a *gag-pol *transcript with one ORF is produced [32]. The *env *transcript is a result of splicing activity, a general strategy for all retroviruses. However, the distantly related *Errantivirus *Cer1, which has all genes in a single ORF (Additional file 2), may possibly represent an original retroelement translational strategy without splicing. A single large polyprotein is also used by some other, even more, distantly related RNA viruses e.g. *Picornaviruses*. The difference in degree of Gag and Pol expression is regulated by a stop codon suppression readthrough after *gag *[11]. This genomic structure is shared with the closest related epsilon and even the intermediate epsilonlike SnRV. Mining in our collected ERV databank, we selected sequences with high RetroTector^© ^scores and analyzed their "putein" reading frames. However, definition of the original proviral ORFs is difficult because of the gradual accumulation of postintegrational indel mutations. To minimize such errors, we excluded sequences with predicted frameshifts near the 3'-end of the respective gene and only included ERVs with RetroTector^© ^scores over 1000, thus ensuring a relatively intact provirus. Results from the remaining 436 elements are shown in table 1. In the gammaretroviral genus (RetroTector^© ^defined using motif similarities to known exogenous, and endogenous, gammaretroviral counterparts), we could detect ERVs with not only the predicted lack of frameshifts, "0 f.s.", but also "-1 f.s.", and "+1 f.s." in the Gag-Pro, and Pro-Pol boundaries (Table 1). However, "0 f.s." between Gag and Pro was detected in 67%, while "+1 f.s." and "-1 f.s." were detected in 16% and 17%, respectively. In the Pro-Pol boundary there were 46%, 31% and 23% for "0 f.s.", "+1 f.s." and "-1 f.s." respectively (Table 1).

Thus there is a propensity, however weaker in Pro-Pol, for gammaretroviral ERVs to enclose their Gag, Pro and Pol in the same reading frame. As a comparison, the analyses of exogenous gammaretroviral FLV and MLV genomic structures are also shown (Additional file 2 and [11]). They are known to use the stop codon suppression mechanism in a single *gag/pro/pol *("0/0") frame. Although this analysis could not be performed for the few rather damaged epsilon-like HERVs [19], the epsilon retrovirus, WDSV, and the epsilon/spumalike intermediate also shared the single gag-pro-pol frame translational strategy with gammaretroviruses (Additional file 2 and [11]).

The betaretroviral ERVs have been described to have a different translational strategy [11]. There were 60% (-1 fs), 22% (0 fs) and 17% (+1 fs) in the Gag-Pro boundary. Between Pro and Pol there were 51% (-1 fs), 22% (0 fs) and 27% (+1 fs) (Table 1). Thus the betaretroviral ERV frame shift propensities, however weaker between Pro and Pol, agree with the predictions according to the related exogenous MMTV and JSRV (Additional file 2) with the Gag, Pro and Pol in different reading frames separated by "-1" frameshifts, a "-1/-1" pattern. This translational strategy is also recognized in the new intermediate betalike group of chicken and reptiles. We also found that the results ("-1/-1") for chicken alpha ERVs (Table 1) deviated somewhat from the expected "0/-1" pattern (see exogenous RSV in additional file 2 and [11]). The computer aided analysis of the exogenous delta and lentiretroviruses conformed with previous descriptions [11]. HIV had "-1/0", whereas HTLV had "-1/+1" in the Gag-Pro and Pro-Pol boundaries, respectively (Additional file 2). To summarize, we find support for similar translational strategies among ERVs and XRVs, although ERV sequences are harder to analyze due to postintegrational frameshifts. Further, two major directions in the Pol phylogeny could be noted (Figure 1). The viral sequences in the left main branch, the "gamma" branch, often have their *gag, pro *and *pol *within the same reading frame. Genera in the right main "beta-branch" (Figure 1), with *gag, pro *and *pol *separated in different ORFs, may use different forms of ribosomal frameshifting [11]. Despite the imprecision of reading frame predictions in ERVs (Table 1), we judge inferred translational strategy to be a "global" marker. It is especially suitable for distinction between the extremes of the major gamma and betaretroviral branches in figure 1.

### Presence of dUTPase

A dUTPase that prevents incorporation of uracil into the retroviral DNA by dUTP degradation, can be advantageous for some retroviruses. A dUTPase was, in compliance with earlier results [33,34], detected by RetroTector^© ^in both betaretroviruses and non-primate lentiviruses (Figure 2B). However, the localization of the dUTPase differs between the genera. Non-primate lentiviral dUTPase is located within the *pol *gene (here dubbed dUTPase^PolA^) [11], whereas the betaretroviral dUTPase is located N-terminal of Pro (here dubbed dUTPase^Pro^). A third dUTPase acquisition event (Additional file 3. Here dubbed dUTPase^PolB^) in MuERV-L [35,36], is located C-terminal of IN. In the ERV dataset, dUTPase^Pro ^was detected in 363 betaretroviral ERVs and 21 intermediate beta-like(alpha-beta) chicken ERVs (Table 1). Thus, many of the chicken intermediate beta-like ERVs lacked detectable dUTPase. Neither could it be found in the intermediate Python ERV (Figure 2B). dUTPase^PolB ^was not tested for.

To investigate the different retroviral acquisitions of dUTPases, we conducted a minimum evolution (ME) analysis, using 389 dUTPase sequences (Additional file 3). The ME tree shows that human betaretroviral dUTPase^Pro ^(HML1-10; [20,37] and Blikstad et al, *in preparation*) and chicken alphabetaretroviral dUTPase^Pro ^(GGERVAB1-14; Blomberg et al, *in preparation*) form one branch together with the more studied mammalian betaretroviral MMTV and MPMV dUTPase^Pro ^sequences. This indicates that dUTPase^Pro ^has a monophyletic origin and was acquired by an alpha-like retrovirus, earlier in evolution than previously suggested (see [38]), just before or during the formation of betaretroviruses, see figure 2B. The absence of dUTPase from the betaretrovirus like non-mammalian Python retroviruses [22] is in approximate accord with this interpretation. Judging from the ME tree, acquisition of dUTPase^PolA ^(by non-primate lentiviruses) and dUTPase^PolB ^(by the spumalike ERV-L) may also have been single events (Additional file 3). The validity of the detected dUTPases is illustrated by the consensus sequences of the conserved motifs, DSDYxGEIQ, IAQLilD and GGFGST (Additional file 4).

### Nucleotide frequency bias

RNA editing, dependent on encapsidation of a host RNA editing enzyme, creates a combination of phenotypic and genotypic traits. In lentiviruses, the host enzyme APOBEC3G is responsible for G to A hypermutation, thus generating an A bias [39]. Although manifested in the retroviral genotype, the nucleotide bias can thus be the result of a phenotypic trait. Nucleotide biases were previously also demonstrated in delta- and betaretroviruses [40]. Using the ERV dataset and the additional XRVs, we confirmed this for lentiviruses, delta- and a subset of gammaretroviruses (Figure 2C), while the spuma-like genus did not show obvious biases [31,40]. Recently we described a group of human gammaretroviral ERVs, the HERV-H-like and adjacent HERV-H- like branching together close to the gammaretroviral root (Figure 2C), to have a uniquely strong G/C bias [31]. In analogy with the lentiviral bias, it is reasonable to assume that HERV-H-like sequences also met an innate antiretroviral defense involving a host RNA editing enzyme. However, the mechanism is unknown and must be different from the cytidine deamination caused by APOBEC3G. Mutational bias caused by the error-prone reverse transcriptase (for a review, see [41]) can also not be ruled out. Reverse transcriptase of different retroviruses has *in vitro *shown different mutational biases [42]. It has been discussed as a contributing factor for the observed skewed nucleotide composition [43].

### C-terminal Protease G-patch domain

Several RNA-binding proteins include a glycine rich domain of about 48 amino acids called "G-patch". This was also present in a betaretroviral MPMV protease C-terminal domain [44]. In self-processing, this domain has been reported to be cleaved from the Pro as a separate protein [45]. The role of this small protein is not determined, but participation in the transport of unspliced retroviral mRNA (see [46]), was suggested [44]. Recently, G-patch was indeed shown to bind single stranded RNA [47]. Further, this G-patch has proved useful in phylogenetic studies, but has shown some inconsistency [48]. In order to extend the phylogenetic investigations and to determine if G-patches are present in other retroviral genera than the described mammalian betaretroviruses, we analyzed the ERV collection for detectable G-patch in the Pro C-terminal domains (Additional file 4). We found 68 positive ERVs (table 1), exclusively within the betaretroviral genus (Figure 2D). Irregularities [48] were also apparent in our Pol phylogeny (Figure 2D), where a G-patch was either degenerated or missing in three of the betaretroviruses, hence uncolored. The validity of the detected G-patch motifs is evident from the consensus sequence, GYx_2_GxGLGx_4_Gx_n_G (Additional file 4). An interesting observation was that dUTPase^Pro ^occurs in avian beta-like intermediate ERVs (Figure 2B), but without the G-patch (Figure 2D). In fact, no chicken betaretrovirus had a detectable G-patch, while dUTPase^Pro ^was often readily detectable. From these data, and those of others [48], we conclude that G-patch entered the genus betaretrovirus after dUTPase^Pro ^and that presence of G-patch may be a useful marker for mammalian betaretroviruses, independent of dUTPase^Pro^.

### C-terminal polymerase IN motif

The C-terminal end of retroviral Pol integrases (IN) has interesting features. Its terminal position allows for addition of functional modules without disturbing the basic integrase functions, represented by the HHCC zinc finger and the DD35E catalytic domains. Alterations in this C-terminal IN domain may alter the specificity of the integration [49]. The C-terminal domain sometimes contains the motif GPY/F (Additional file 4 and [50]). To this domain, another "chromo" (chromatin-binding) -domain is sometimes appended [50], which interacts with chromatin via DNA-binding proteins [49,51]. Recently, we showed that HERV-H and ERV3 have GPY/F-domains [16,31]. Here we used our ERV collection to extend the analysis. We found 264 ERVs with GPY/F motifs (Table 1). A larger portion had a similar mutated, but still detectable, C-terminal IN region (data not shown). An extended consensus GPY/F motif of the ERVs was computed, Wx_n_GPyxV (Additional file 4). Its typical sequence demonstrates the validity of the detected GPY/F motifs. All these ERVs were gammaretroviral. No betaretroviral element was detected with this domain (Figure 2D). Further, GPY/F motifs were found in epsilon, delta, lenti and errantiviruses (Figure 2D). Thus, in figure 2D, we can demarcate a line where GPY/F, or mutated remnant motifs, can be detected to the left, from the lentiviral branch towards gammaretroviruses, in analogy to how the translational strategies (see above) separated the Pol tree into two major branches.

### Accessory genes

**Figure 3:**
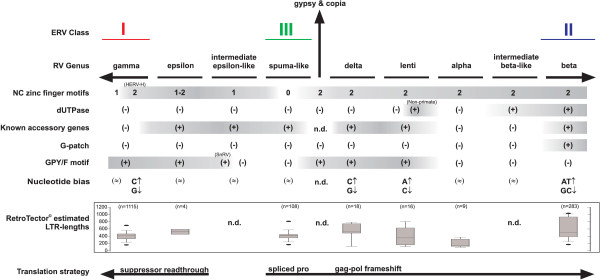
**Structural traits summary**. Simplified view of the different genotypic traits suggested for retroviral phylogeny inference. The branch for Gypsy and Copia represent an imagined midpoint reference in the tree. The number of NC zinc fingers, presence of dUTPase (dUTPase^PolB^ is not indicated), known accessory genes, C-terminal Pro (G-patch) and Pol (GPY/F) motifs are shown. Nucleotide bias was defined to 25 ± 5 %. (↑) shifted upwards; (↓) shifted downwards; (≈) uncertain bias. Exploration of the LTR lengths of the different groups as detected by RetroTector^© ^are shown as boxplots. In addition, the translational strategy may be used in the phylogeny to separate the gammaretroviruses (including class I ERVs) from spuma-like elements (class III ERVs), deltaretroviruses, lentiviruses, alpharetroviruses and the betaretroviruses (class II ERVs) with respective intermediate groups. The Gypsy and Copia are not included in the translational strategy analysis.

The presence of accessory genes in complex retroviruses can also be used for evolutionary inference (Figure 3). Recognition of unknown accessory genes is a difficult bioinformatic problem and absence of accessory genes is hard to ascertain. The analysis therefore rests on demonstrable ones. The delta and lenti genera have several accessory genes with similar functions as integral parts of their replication strategy. They can to some extent replace each other; *rex *and *rev*, *tax *and *tat *[11]. The sometimes drastic influences of these *trans*-activating gene products on cellular functions may have kept these viruses out of the germline. Recently, the betaretroviral HERV-K(HML2) was shown to have the accessory genes, *rec *and/or *np9 *[52,53], and is thus a complex retrovirus. *rec *is at least functionally related to *rev *and *rex *[54]. Also the epsilon (WDSV) and spumaretroviruses have accessory ORFs, Orf1, 2 and 3, and Bel etc., respectively [11]. The phylogeny of accessory genes (see [55]) is a separate issue, which we do not study further here. From the available information, the accessory genes mainly contribute to rather local properties in the retroviral tree.

### LTR lengths

As a final point in the conceptual use of structural traits in phylogenies, a brief exploration of LTR lengths showed a significant difference between the most distantly related gamma and beta genera, where gammaretroviral LTRs are short and betaretroviral LTRs significantly longer (Figure 3). LTR length is therefore a useful additional property for the distinction of these genera.

## Conclusion

Inferring phenotypic traits and phylogenies from interpreted genotypic (sequence) ERV properties is similar to the use of fossilized remains for similar purposes in paleontology. The analysis will gather strength with increasing numbers of analyzed host genomes. Pol similarities and structural traits like the ones discussed here, contribute to a better understanding of the retroviral phylogeny. There are at least two major retroviral branches. One contains the gammaretroviruses (including class I ERVs) together with the epsilonretroviruses, and another which includes betaretroviruses (including class II ERVs) together with delta, lenti and alpharetroviruses with their respective intermediate groups. In between, closer to an imaginary root of the retroviral evolutionary tree, we find the older spuma and spuma-like (class III ERVs) retroviruses. The two major branches, schematized in figure 3, differ in "global" genomic properties as **i.) **translational strategy, **ii.) **number of Gag NC zinc finger motifs, **iii.) **presence of dUTPase, **iv.) **presence of Pro C-terminal G-patch and **v.) **presence of GPY/F motifs in the IN C-terminal domain. "Local" retroviral properties useful for more narrow delineation of taxa are **i.) **host species, **ii.) **nucleotide compositional bias and **iii.) **LTR lengths.

## Methods

### Data collection

Genomic data were downloaded from the UCSC genome browser , and annotated retroviral reference sequences included in the phylogenies were extracted from GenBank .

GenBank accession numbers or chromosomal positions in *Homo sapiens *(version hg16 and 15) for reference sequences in the main phylogenetic tree were as follows: ALV [NC001408], RSV [NC001407], MMTV [NC001503], MPMV [NC001550], JSRV [M80216], HML1 (Chr19-21849393), HML2 (Chr11-101600013), HML3 (Chr1-48344461), HML4 (Chr8-75679221), HML5 [AC004536], HML6 (consensus), HML7 (Chr6-121300220), HML8 (Chr3-131452286), HML9 (Chr9-62700428), HML10 (Chr6-32017925), HERV-H (consensus), HERV-H/RGH2 [D11078], HERV-H/RTVLH2 [M18048], HERV-Fc1 [AL354685], HERV-Fc2 [AC019088], HERV-W (Chr7-9105739), ERV9 [AC073410], ERV3 (Chr7-63865366), HERV-E [M10976], MLV [NC001501], MoLV [AF033811], BaEV [D10032], GaLV [M26927], HERV-ADP [AC005741], HERV-FRD [AC004022], HERV-I (Chr16-72821350), HERV-T (Chr14-104635791), HERV-S [AC004385], FLV [NC001940], PERV [AJ293656], WDSV [NC001867], Xen1 [AJ506107], SnRV [NC001724], BLV [NC001414], HTLV-1 [NC001436], and HTLV-2 [NC001488], Gypsy [AJ000387], HERV-L (RepBase), HSRV [AF033816], HFV [NC001736], MER4like (Chr13-54208300), HERV-L66 (RepBase), HERV-L74 (RepBase), HERV-L40 (RepBase) and Python molurus [AAN77283].

### Endogenous retroviral sequences

We used the bioinformatic program RetroTector^©^, developed by us, to screen the downloaded genomic sequences for proviral integrations. Briefly, the program recognizes conserved retroviral consensus motifs and constructs putative proteins ("puteins") from the different reading frames in the gene candidates. Codon statistics, frequency of stop codons and alignment to known retroviral proteins are used to approximate an original ORF. Finally the puteins are validated and classified using alignments of earlier described proteins from the literature. The validity of the puteins used for alignment and phylogenetic inference, can be confirmed by inspection of excised parts of RT and IN from the full Pol alignment (Additional file 5). The program yields a preliminary genus classification based on motif usage. In several papers, the computerized motif based preliminary retroviral classification was shown to be consistent and robust with reference to other means of classification [16,17,19]. Using a RetroTector^© ^cutoff score of more than 300, we found 3149 proviral sequences in the human genome version hg16 [4] and 260 proviral sequences in the chicken gg01 [21], which were included into our ERV databank. From this databank, we could extract representative proviral sequences for later analyses. The extracted representative sequences had high RetroTector^© ^scores and were selected for their contribution to phylogenetic reconstruction, with preference for intermediates between previously recognized retroviral genera (see figure. 1)

### Data analysis

Multiple alignments were conducted using ClustalX (1.83) [56]. A consensus NJ was produced in MEGA2.1 [57] using the pairwise deletion option, Poisson amino acid correction and 500 bootstraps. A set of maximum likelihood analyses using the PHYLIP program package [58] were used to verify the tree topologies. Consensus analysis of C-terminal Pro (G-patch) and Pol (GPY/F) motifs were conducted using WebLogo at , with default settings

Statistics were extracted from the ERV databank collected through the RetroTector^© ^analysis of the different genomes.

The Pol FASTA sequences are included into the additional files (Additional file 6).

## List of Abbreviations used

aa amino acids

CA Capsid

dUTPase deoxyuridine triphosphatase

Env Envelope

ERVs Endogenous retroviral sequences

Gag Group specific antigen

HERVs Human endogenous retroviral sequences

IN Integrase

LTR Long terminal repeat

MA Matrix

NC Nucleocapsid

PBS Primer binding site

Pol Polymerase

PPT Polypurine tract

Pro Protease

RNAse H Ribonuclease H enzyme

RT Reverse transcriptase

XRV Exogenous retrovirus

## Competing interests

The author(s) declare that they have no competing interests.

## Authors' contributions

**PJ **conceived the study, participated in its design and coordination, carried out the molecular genetic studies and drafted the manuscript. **GOS **developed algorithms and carried out the programming needed for the analyses. **JB **conceived the study, and participated in its design and coordination, participated in programming and helped to draft the manuscript. All authors read and approved the final manuscript.

## Supplementary Material

Additional File 1 **Pol similarity matrix**. Pol NJ cladogram (1000 bootstraps and pairwise deletions) aligned to a similarity matrix based on PAM250. Click here for file

Additional File 2 **Retroviral genomic structures**. RetroTector^© ^output of selected retroviral genomic structures described in the text. Click here for file

Additional File 3 **dUTPase phylogenetic tree**. Retroviral dUTPase acquisitions. Minimum Evolution (ME) tree (100 bootstraps). Click here for file

Additional File 4 **Motif consensus validations**. WebLogo consensus of the partial dUTPase^Pro^, C-terminal Pro (G-patch) and Pol (GPY/F) motifs. Click here for file

Additional File 5 **Putein validations**. Validation of puteins from Pol alignment. Excised parts of RT and IN are shown. Click here for file

Additional File 6 **Pol FASTA sequences**. Pol FASTA sequences. Click here for file
