# Supplementary material for: Use of Endogenous Retroviral Sequences (ERVs) and structural markers for retroviral phylogenetic inference and taxonomy
Source: Retrovirology. 2005 Aug 10;2:50. doi: 10.1186/1742-4690-2-50 (PMC1224870; doi:10.1186/1742-4690-2-50)
Supplement: Additional File 1 — Pol similarity matrix. Pol NJ cladogram (1000 bootstraps and pairwise deletions) aligned to a similarity matrix based on PAM250. [file 1742-4690-2-50-S1.pdf]

Pol similarity matrix of endogenous retroviruses (ERVs) and reference sequences

Percent similarity using pairwise deletions and PAM250. The shortest sequence of the two aligned to itself equals 100 % identity.

>80 % Pol similarity is highlighted in red

>90 % Pol similarity is highlighted in **red and bold**

ERVs are presented with chromosome positions in respective genomes

hg15: *Homo sapiens* genome version 15

pt01: *Pan troglodytes* genome version 1

gg01: Gallus gallus genome version 1

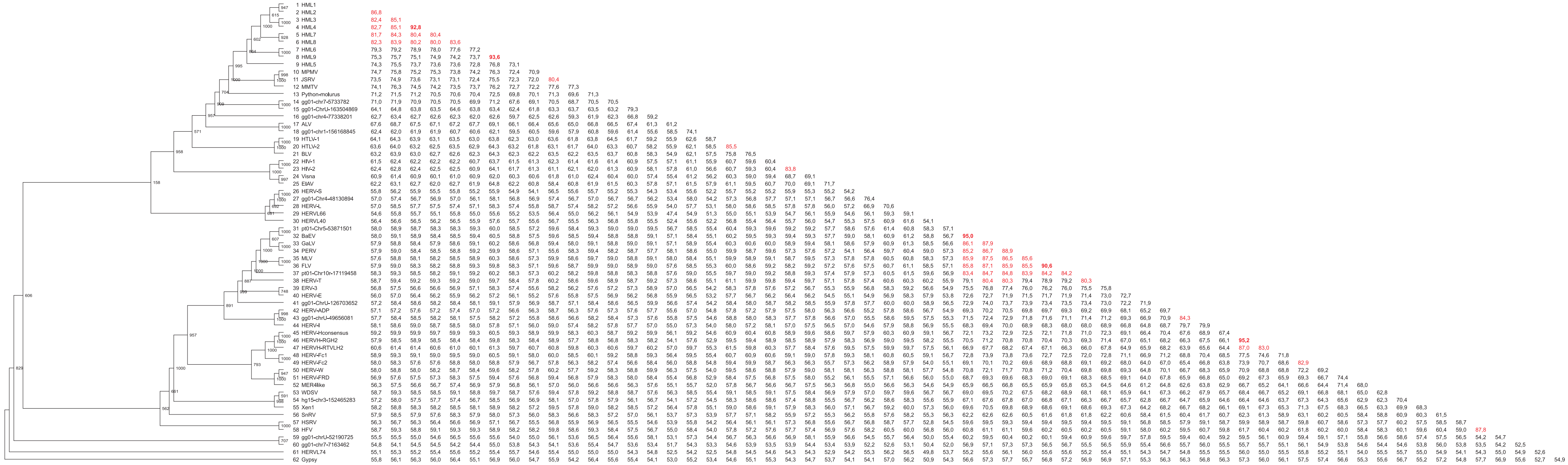

	1	2	3	4	5	6	7	8	9	10	11	12	13	14	15	16	17	18	19	20	21	22	23	24	25	26	27	28	29	30	31	32	33	34	35	36	37	38	39	40	41	42	43	44	45	46	47	48	49	50	51	52	53	54	55	56	57	58	59	60	61	62
1 HML1																																																														
2 HML2	86,8																																																													
3 HML3	82,4	85,1																																																											
4 HML4	82,7	85,1	**92,8**																																																									
5 HML7	81,7	84,3	80,4	80,4																																																							
6 HML8	82,3	83,9	80,2	80,0	83,6																																																					
7 HML6	79,3	79,2	78,9	78,0	77,6	77,2																																																				
8 HML9	75,3	75,7	75,1	74,9	74,2	73,7	**93,6**																																																			
9 HML5	74,3	75,5	73,7	73,6	73,6	72,8	76,8	73,1																																																	
10 MPMV	74,7	75,8	75,2	75,3	73,8	74,2	76,3	72,4	70,9																																																
11 JSRV	73,5	74,9	73,6	73,1	73,1	72,4	75,5	72,3	72,0	80,4																																														
12 MMTV	74,1	76,3	74,5	74,2	73,5	73,7	76,2	72,7	72,2	77,6	77,3																																										
13 Python-molurus	71,2	71,5	71,2	70,5	70,6	70,4	72,5	69,8	70,1	71,3	69,6	71,3																																									
14 gg01-chr7-5733782	71,0	71,9	70,9	70,5	70,5	69,9	71,2	67,6	69,1	70,5	68,7	70,5	70,5																																								
15 gg01-ChrU-163504869	64,1	64,8	63,8	63,5	64,6	63,8	63,4	62,4	61,8	63,3	63,7	63,5	63,2	79,3																																							
16 gg01-chr4-77338201	62,7	63,4	62,7	62,6	62,3	62,0	62,6	59,7	62,5	62,6	59,3	61,9	62,3	66,8	59,2																																						
17 ALV	67,6	68,7	67,5	67,1	67,2	67,7	69,1	66,1	66,4	65,6	65,0	66,8	66,5	67,4	61,3	61,2																																					
18 gg01-chr1-156168845	62,4	62,0	61,9	61,9	60,7	60,6	62,1	59,5	60,5	59,6	57,9	60,8	59,6	61,4	55,6	58,5	74,1																																				
19 HTLV-1	64,1	64,3	63,9	63,1	63,5	63,0	63,8	62,3	63,0	63,6	61,8	63,8	64,5	61,7	59,2	55,9	62,6	58,7																																			
20 HTLV-2	63,6	64,0	63,2	62,5	63,5	62,9	64,3	63,2	61,8	63,1	61,7	64,0	63,3	60,7	58,2	55,9	62,1	58,5	85,5																																		
21 BLV	63,2	63,9	63,0	62,7	62,6	62,3	64,3	62,3	62,2	63,5	62,2	63,5	63,7	60,8	58,3	54,9	62,1	57,5	75,8	76,5																																	
22 HIV-1	61,5	62,4	62,2	62,2	62,2	60,7	63,7	61,5	61,3	62,3	61,4	61,6	61,4	60,9	57,5	57,1	61,1	55,9	60,7	59,6	60,4																																
23 HIV-2	62,4	62,8	62,4	62,5	62,5	60,9	64,1	61,7	61,3	61,1	62,1	62,0	61,3	60,9	58,1	57,8	61,0	56,6	60,7	59,3	60,4	83,8																															
24 Visna	60,9	61,4	60,9	60,1	61,0	60,9	62,0	60,3	60,6	61,8	61,0	62,4	60,4	60,0	57,4	55,4	61,2	56,2	60,3	59,0	59,4	68,7	69,1																														
25 EIAV	62,2	63,1	62,7	62,0	62,7	61,9	64,8	62,2	60,8	58,4	60,8	61,9	61,5	60,3	57,8	57,1	61,5	57,9	61,1	59,5	60,7	70,0	69,1	71,7																													
26 HERV-S	55,8	56,2	55,9	55,5	55,8	55,2	55,9	54,9	54,1	56,5	55,6	55,7	55,2	55,3	54,3	53,4	55,6	52,2	55,7	55,2	55,2	55,9	55,3	55,2	54,2																												
27 gg01-Chr4-48130894	57,0	57,4	56,7	56,9	57,0	56,1	58,1	56,8	56,9	57,4	56,7	57,0	56,7	56,7	56,2	53,4	58,0	54,2	57,3	56,8	57,7	57,1	57,1	56,7	56,6	76,4																											
28 HERV-L	57,0	58,5	57,7	57,5	57,4	57,1	58,3	57,4	55,8	58,7	57,4	58,2	57,2	56,6	55,9	54,0	57,7	53,1	58,0	58,6	58,5	57,8	57,8	56,0	57,2	66,9	70,6																										
29 HERVL66	54,6	55,8	55,7	55,1	55,8	55,0	55,6	55,2	53,5	56,4	55,0	56,2	56,1	54,9	53,9	47,4	54,9	51,3	55,0	55,1	53,9	54,7	56,1	55,9	54,6	56,1	59,3	59,1																									
30 HERVL40	56,4	56,6	56,5	56,2	56,5	55,9	57,6	55,7	55,6	56,7	55,5	56,3	56,8	55,8	55,5	52,4	55,6	52,2	56,8	55,4	56,4	55,7	56,0	54,7	55,3	57,5	60,9	61,6	54,1																								
31 pt01-Chr5-53871501	58,0	58,9	58,7	58,3	58,3	59,3	60,0	58,5	57,2	59,6	58,4	59,3	59,0	59,0	59,5	56,7	58,5	55,4	60,4	59,3	59,6	59,2	59,2	57,7	58,6	57,6	61,4	60,8	58,3	57,1																							
32 BaEV	58,0	59,1	58,9	58,4	58,5	59,4	60,5	58,8	57,5	59,6	58,5	59,4	58,8	58,8	59,1	57,1	58,4	55,1	60,2	59,5	59,3	59,4	59,3	57,7	59,0	58,1	60,9	61,2	58,8	56,7	**95,0**																						
33 GaLV	57,9	58,8	58,4	57,9	58,6	59,1	60,2	58,6	56,8	59,4	58,0	59,1	58,8	59,0	59,1	57,1	58,9	55,4	60,3	60,6	60,0	58,9	59,4	58,1	58,6	57,9	60,9	61,3	58,5	56,6	86,1	87,9																					
34 PERV	57,9	59,0	58,4	58,5	58,8	59,2	59,9	58,6	57,1	55,6	58,3	59,4	58,2	58,7	57,7	58,1	58,6	55,0	59,9	58,7	59,6	57,3	57,6	57,2	54,1	56,4	59,7	60,4	59,0	57,3	85,2	86,7	88,9																				
35 MLV	57,6	58,8	58,1	58,2	58,5	58,9	60,3	58,6	57,3	59,9	58,6	59,7	59,0	58,8	59,1	58,0	58,4	55,1	59,9	58,9	59,1	58,7	59,5	57,3	57,8	57,8	60,5	60,8	58,3	57,3	85,9	87,5	86,5	85,6																			
36 FLV	57,9	59,0	58,3	58,2	58,8	59,3	59,8	58,3	57,1	59,6	58,7	59,9	59,0	58,9	59,0	57,6	58,5	55,3	60,0	58,6	59,2	58,2	59,2	57,2	57,6	57,5	60,7	61,1	58,5	57,1	85,8	87,1	85,9	85,5	**90,6**																		
37 pt01-Chr10r-17119458	58,3	59,3	58,5	58,2	59,1	59,2	60,2	58,3	57,3	60,2	58,2	59,8	58,8	58,3	58,8	57,6	59,0	55,5	59,7	59,0	59,2	58,8	59,3	57,4	57,9	57,3	60,5	61,5	59,6	56,9	83,4	84,7	84,8	83,9	84,2	84,2																	
38 HERV-T	58,7	59,4	59,2	59,3	59,2	59,0	59,7	58,4	57,8	60,2	58,6	59,6	58,9	58,7	59,2	57,3	58,6	55,1	61,1	59,9	59,8	59,4	59,7	57,1	57,8	57,4	60,6	60,3	60,2	55,9	79,1	80,4	80,3	79,4	78,9	79,2	80,3																
39 ERV-3	56,8	57,5	56,6	56,6	56,9	57,1	58,3	57,4	55,6	58,2	56,2	57,6	57,2	57,3	58,9	57,0	56,5	54,2	58,3	57,8	57,6	57,2	56,7	55,3	55,9	56,8	58,3	59,2	56,6	54,9	75,5	76,8	77,4	76,0	76,2	76,0	75,5	75,8															
40 HERV-E	56,0	57,0	56,4	56,2	55,9	56,2	57,2	56,1	55,2	57,6	55,8	57,5	56,9	56,2	56,8	55,9	56,5	53,2	57,7	56,7	56,2	56,4	56,2	54,5	55,1	54,9	56,9	58,3	57,9	53,8	72,6	72,7	71,9	71,5	71,7	71,9	71,4	73,0	72,7														
41 gg01-ChrU-126703652	57,2	58,4	58,6	58,2	58,4	58,1	59,1	57,9	56,9	58,7	57,1	58,4	58,6	56,5	59,9	56,6	57,4	54,2	58,4	58,0	58,7	58,2	58,5	55,9	57,8	57,7	60,0	60,0	58,9	56,5	72,9	74,0	73,7	73,9	73,4	73,5	73,4	73,0	72,2	71,9													
42 HERV-ADP	57,1	57,2	57,6	57,2	57,4	57,0	57,2	56,6	56,3	58,7	56,3	57,6	57,3	57,6	57,7	55,6	57,0	54,8	57,8	57,2	57,9	57,5	58,0	56,3	56,6	55,2	57,8	58,6	56,7	54,9	69,3	70,2	70,5	69,8	69,7	69,3	69,2	69,9	68,1	65,2	69,7												
43 gg01-chrU-49656081	57,7	58,4	58,5	58,2	58,1	57,5	58,2	57,2	55,8	58,6	56,6	58,2	58,4	57,3	57,6	55,8	57,5	54,6	58,8	58,0	58,3	57,7	57,8	56,6	57,0	55,5	58,6	59,5	57,5	55,3	71,5	72,4	72,9	71,8	71,6	71,1	71,4	71,2	69,3	66,9	70,9	84,3																				
44 HERV-I	58,1	58,6	59,0	58,7	58,5	58,0	57,8	57,1	56,0	59,0	57,4	58,2	57,8	57,7	57,0	55,0	57,3	54,0	58,0	57,2	58,1	57,0	57,5	56,5	57,0	54,6	57,9	58,8	56,9	55,5	68,3	69,4	70,0	68,9	68,3	68,0	68,0	68,9	66,8	64,8	68,7	79,7	79,9																			
45 HERV-Hconsensus	59,2	59,9	59,9	59,7	59,9	59,3	60,5	59,3	58,9	59,9	58,3	60,3	58,7	59,2	59,9	56,1	59,2	54,6	60,9	60,4	60,8	58,9	59,6	58,6	59,7	57,9	60,3	60,9	59,1	56,7	72,1	73,2	72,9	72,5	72,1	71,8	71,0	72,3	69,1	66,4	70,4	67,6	68,9	67,4																		
46 HERVH-RGH2	57,9	58,5	58,9	58,5	58,4	58,4	59,8	58,3	58,4	58,9	57,7	58,8	56,8	58,3	58,2	54,1	57,6	52,9	59,5	59,4	58,9	58,5	58,9	57,9	58,3	56,9	59,0	59,5	58,2	55,5	70,5	71,2	70,8	70,8	70,4	70,3	69,3	71,4	67,0	65,1	68,2	66,3	67,5	66,1	**95,2**																	
47 HERVH-RTVLH2	60,6	61,4	61,4	60,6	61,0	60,1	61,3	59,7	60,7	60,8	59,8	60,3	60,6	59,7	60,2	57,0	59,7	55,3	61,5	59,8	60,3	57,7	58,4	57,6	59,5	57,5	59,9	59,7	57,5	56,1	66,9	67,7	68,2	67,4	67,1	66,3	66,0	67,8	64,9	65,9	68,2	63,9	65,6	64,4	87,0	83,0																
48 HERV-Fc1	58,9	59,3	59,1	59,0	59,5	59,0	60,5	59,1	58,0	60,0	58,5	60,1	59,2	58,8	59,3	56,4	59,5	55,4	60,7	60,9	60,6	59,1	59,0	57,8	59,3	58,1	60,8	60,5	59,1	56,7	72,8	73,9	73,8	73,6	72,7	72,5	72,0	72,8	71,1	66,9	71,2	68,8	70,4	68,5	77,5	74,6	71,8															
49 HERV-Fc2	58,0	58,3	57,6	57,6	58,8	58,0	58,8	57,9	56,7	57,8	56,3	58,2	57,4	56,6	58,4	56,0	58,8	54,8	59,4	59,9	58,7	56,3	56,3	55,7	57,3	56,2	58,9	57,9	54,0	55,1	69,1	70,1	70,2	69,6	68,9	68,8	69,1	69,2	68,0	64,0	67,0	65,4	66,8	63,8	73,9	70,7	68,6	82,9														
50 HERV-W	58,0	58,8	58,0	58,2	58,7	58,4	59,6	58,2	57,8	60,2	57,7	59,2	58,3	58,8	59,9	56,3	57,5	54,0	59,5	58,6	58,8	57,9	59,0	58,1	58,1	56,3	58,8	58,1	57,7	54,8	70,8	72,1	71,7	70,8	71,2	70,4	69,8	69,8	69,3	64,8	70,1	66,7	68,3	65,9	70,9	68,8	68,8	72,2	69,2													
51 HERV-FRD	56,9	57,6	57,5	57,3	58,3	57,5	59,4	57,6	56,8	59,4	56,8	57,9	58,3	58,0	58,4	55,4	56,8	52,9	58,4	57,5	56,8	57,5	58,0	55,2	56,1	55,5	57,1	56,6	56,0	55,0	68,7	69,3	69,6	68,3	69,0	69,1	68,3	68,5	69,1	64,0	67,8	65,9	66,8	65,0	69,2	67,3	65,9	69,3	66,7	74,4												
52 MER4like	56,3	57,5	56,6	56,7	57,4	56,9	57,9	56,8	56,1	57,0	56,0	56,6	56,6	56,3	57,6	55,1	55,7	52,0	57,8	56,7	56,6	56,7	57,5	56,3	56,8	55,0	56,6	56,3	54,6	54,9	65,9	66,5	66,8	65,5	65,9	65,8	65,3	64,5	64,6	61,2	64,8	62,6	63,8	62,9	66,7	65,2	64,1	66,6	64,4	71,4	68,0											
53 WDSV	58,7	59,3	58,5	58,5	59,1	58,8	59,7	58,7	57,6	59,4	57,8	59,2	58,8	58,7	57,6	56,3	58,5	55,4	59,1	58,5	59,1	57,5	58,4	56,9	57,9	57,0	59,7	59,6	56,7	56,7	69,0	69,5	70,2	67,5	68,2	68,9	68,1	68,1	65,9	64,1	67,3	66,2	67,9	65,7	68,4	66,7	65,2	69,1	66,8	68,1	65,0	62,8										
54 hg15-chr3-152465283	57,2	58,0	57,5	57,7	57,4	56,7	58,5	56,9	56,9	58,1	57,0	57,8	57,9	56,1	56,7	54,1	57,2	54,5	58,3	58,6	58,6	57,4	58,8	55,5	56,7	56,2	58,6	58,3	55,6	55,9	67,1	67,6	67,8	67,0	66,8	67,1	66,3	66,7	65,7	62,8	66,7	64,7	65,9	64,6	66,4	64,6	63,7	67,3	64,3	65,6	62,9	62,3	70,4									
55 Xen1	58,2	58,8	58,3	58,2	58,5	58,1	58,9	58,2	57,2	59,5	57,8	59,0	58,2	58,5	57,2	56,4	57,8	55,1	59,0	58,6	59,1	57,9	58,3	56,0	57,1	56,7	59,2	60,0	57,3	56,0	69,6	70,5	69,8	68,9	68,6	69,1	68,6	69,3	67,3	64,2	68,2	66,7	68,2	66,1	69,1	67,3	65,3	71,3	67,5	68,3	66,5	63,3	69,9	68,3								
56 SnRV	57,9	58,5	57,9	57,6	58,3	57,9	58,0	57,3	56,0	58,3	56,6	58,3	57,2	57,0	56,1	53,7	57,3	53,9	57,7	57,1	58,2	55,9	57,2	55,3	56,2	55,8	57,6	58,2	55,3	56,3	62,2	62,6	62,6	60,5	61,6	61,8	61,8	62,2	60,6	58,4	61,5	60,4	61,7	60,7	62,3	61,3	58,9	63,1	60,2	60,5	58,4	58,8	60,9	60,3	61,5							
57 HSRV	56,3	56,7	56,3	56,4	56,6	56,9	57,1	56,7	55,5	56,8	55,9	56,9	56,5	55,5	54,6	53,9	55,8	54,2	56,4	56,1	56,1	57,3	56,8	55,6	56,7	56,8	58,7	57,7	52,8	54,5	59,6	59,5	59,3	59,4	59,4	59,5	59,4	59,5	59,1	56,8	58,5	57,9	59,1	58,7	59,9	58,9	58,7	59,8	60,7	58,6	57,3	57,7	60,2	57,5	58,5	58,7						
58 HFV	58,7	59,3	58,8	59,1	59,3	59,3	58,9	58,2	58,2	59,8	58,6	59,3	58,4	57,5	56,7	55,0	58,4	54,0	57,8	57,2	57,6	57,7	57,4	56,9	57,6	58,2	60,5	60,0	56,8	56,0	60,8	61,1	61,1	59,6	60,2	60,5	60,2	60,5	59,1	58,0	60,2	59,5	60,7	59,8	61,7	60,4	60,2	61,8	60,2	60,0	58,4	58,3	60,1	59,6	60,4	59,0	87,8					
59 gg01-chrU-52190725	55,5	55,5	55,0	54,6	56,5	55,6	55,6	54,0	55,0	56,1	53,6	56,5	56,4	55,6	58,1	53,1	57,3	54,4	56,7	56,3	56,6	56,9	58,1	55,9	56,6	54,5	55,7	56,4	50,0	55,4	60,2	59,5	60,4	60,2	60,1	59,4	60,9	59,6	59,7	57,8	59,5	59,4	60,4	59,2	59,5	56,1	60,9	59,4	59,1	57,1	55,8	56,6	58,6	57,4	57,5	56,5	54,2	54,7				
60 gg01-chr7-7163462	54,8	54,1	54,5	54,5	54,2	54,4	55,0	53,8	54,3	54,1	53,6	55,4	54,7	53,6	53,4	51,7	54,3	53,3	54,6	53,9	53,5	53,9	54,4	53,4	53,9	52,2	52,6	53,1	50,4	52,0	56,9	57,1	57,3	57,3	56,5	56,7	55,5	56,5	55,9	55,4	56,6	55,7	56,0	55,5	55,7	55,7	55,1	56,1	54,9	53,8	54,6	54,4	54,6	53,8	56,0	53,8	53,5	54,2	52,5			
61 HERVL74	55,1	55,3	55,2	55,4	55,6	55,2	55,4	55,7	54,6	55,4	55,0	55,0	55,0	54,3	54,8	52,5	54,2	52,5	54,8	54,5	54,6	54,3	54,3	52,9	54,2	55,3	56,2	56,5	49,8	53,7	55,2	55,6	56,1	56,0	55,6	55,6	55,2	55,4	55,1	54,3	54,7	54,8	55,5	55,5	56,0	55,0	55,5	55,8	55,2	55,1	54,0	55,5	55,7	55,0	54,9	54,1	54,3	55,0	54,9	52,6		
62 Gypsy	55,8	56,1	56,3	56,0	56,4	55,1	56,9	56,0	54,7	55,9	54,2	56,4	55,6	55,4	54,1	53,0	55,2	53,4	54,6	55,1	55,3	54,3	54,7	53,7	54,1	54,1	57,0	56,2	50,9	54,3	56,6	57,3	57,7	55,7	56,8	57,2	56,9	56,9	57,1	55,3	56,3	56,3	56,8	56,3	57,3	56,0	56,1	57,5	57,4	56,6	55,3	55,6	56,7	55,2	57,2	54,8	57,7	56,9	55,6	52,7	54,9	
