# Supplementary material for: Use of Endogenous Retroviral Sequences (ERVs) and structural markers for retroviral phylogenetic inference and taxonomy
Source: Retrovirology. 2005 Aug 10;2:50. doi: 10.1186/1742-4690-2-50 (PMC1224870; doi:10.1186/1742-4690-2-50)
Supplement: Additional File 2 — Retroviral genomic structures. RetroTector© output of selected retroviral genomic structures described in the text. [file 1742-4690-2-50-S2.pdf]

Selected RetroTector© outputs

Selected endogenous retroviruses (ERVs) and exogenous retroviruses (XRVs) extracted from the human genome (hg16), chicken genome (gg01) and from annotations in GenBank.

(Graphic display of LTRs depend on the availability of full LTRs [U5-R-U3].)

(Graphic display quality of ERVs depend on their ages [accumulated mutations])

Legend

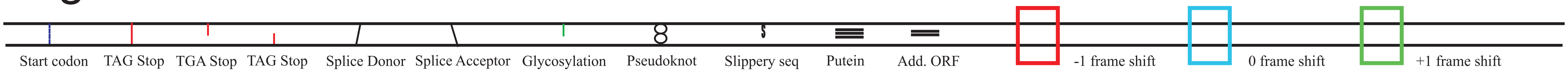

Motifs

5LT **5'LTR**

PBS **Primer binding site**

Gag **Group specific antigen**

MA1, MA2 .... Matrix
CA0, CA1, CA2 .... Capsid
NC1, NC2 .... Nucleocapsid (zinc fingers)

Pro **Protease**

DU0, DU1, DU2 .... dUTPase (deoxyuridine triphosphatase)
PR1, PR2, PR3 .... Protease

Pol **Polymerase**

RT1, RT2, RT3, RT4, RT5, RT6 .... Reverse transcriptase
DL1, DL2 .... dUTPase (deoxyuridine triphosphatase)
In1, IN2, IN3, IN4, IN5, IN6, IN7 .... Integrase

Env **Envelope**

SU2, SU3 .... Surface unit
TM2, TM3, TM4, TM5 .... Transmembrane protein

PPT **Polypurine tract**

3LT **3'LTR**

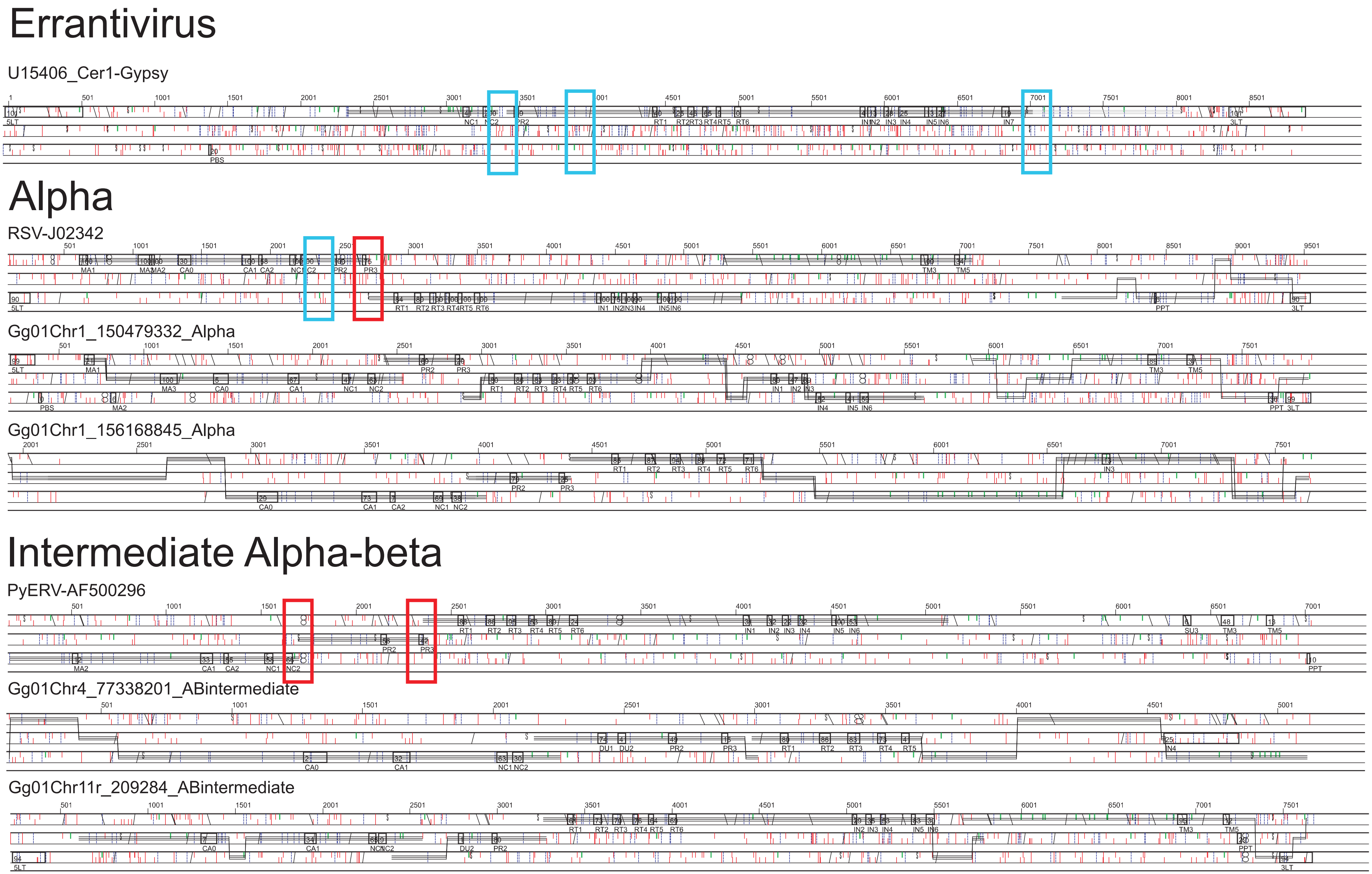

Errantivirus
U15406_Cer1-Gypsy
Alpha
RSV-J02342
Gg01Chr1_150479332_Alpha
Gg01Chr1_156168845_Alpha
Intermediate Alpha-beta
PyERV-AF500296
Gg01Chr4_77338201_ABintermediate
Gg01Chr11r_209284_ABintermediate

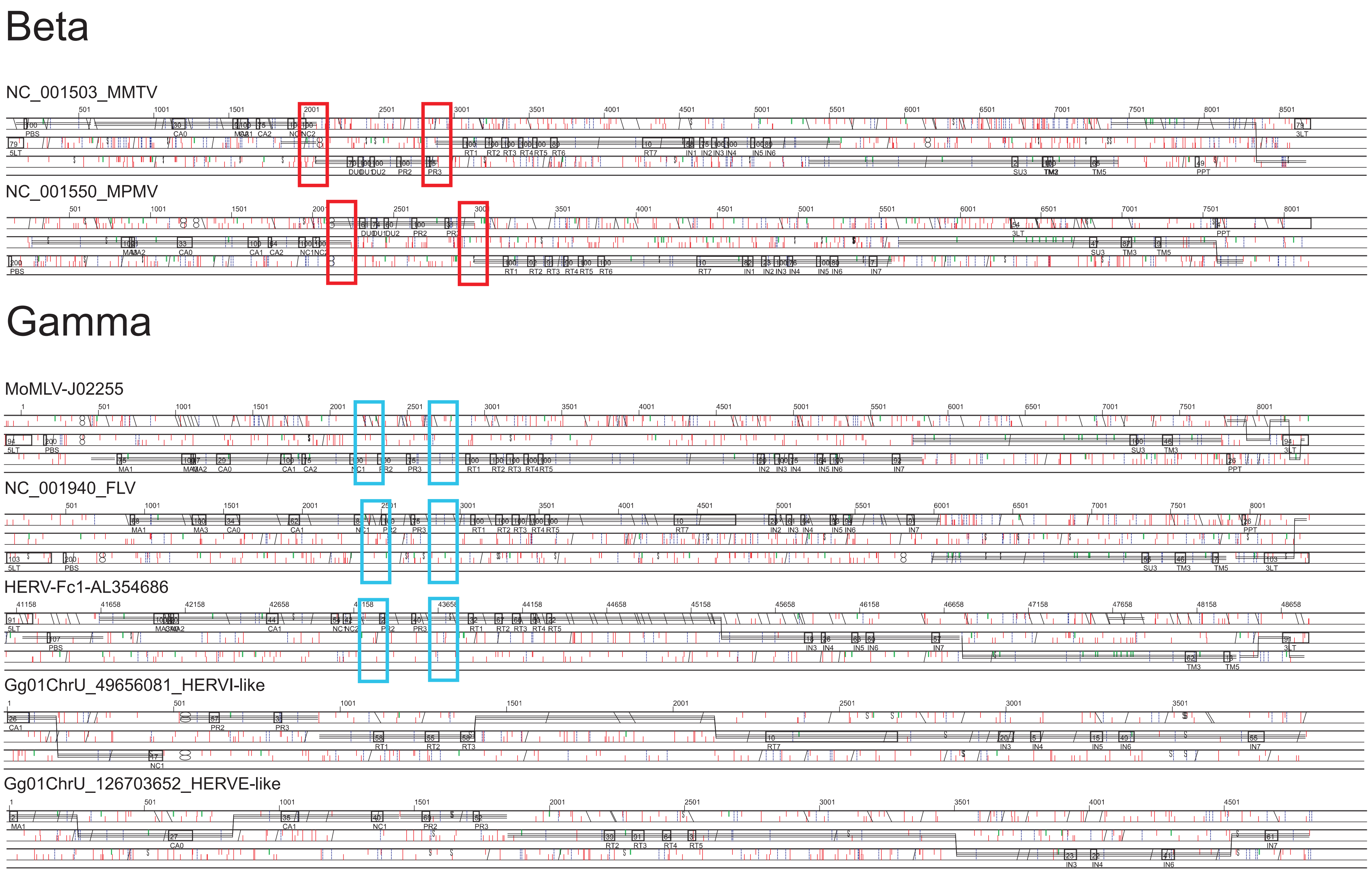
Beta
NC_001503_MMTV
NC_001550_MPMV
Gamma
MoMLV-J02255
NC_001940_FLV
HERV-Fc1-AL354686
Gg01ChrU_49656081_HERVI-like
Gg01ChrU_126703652_HERVE-like

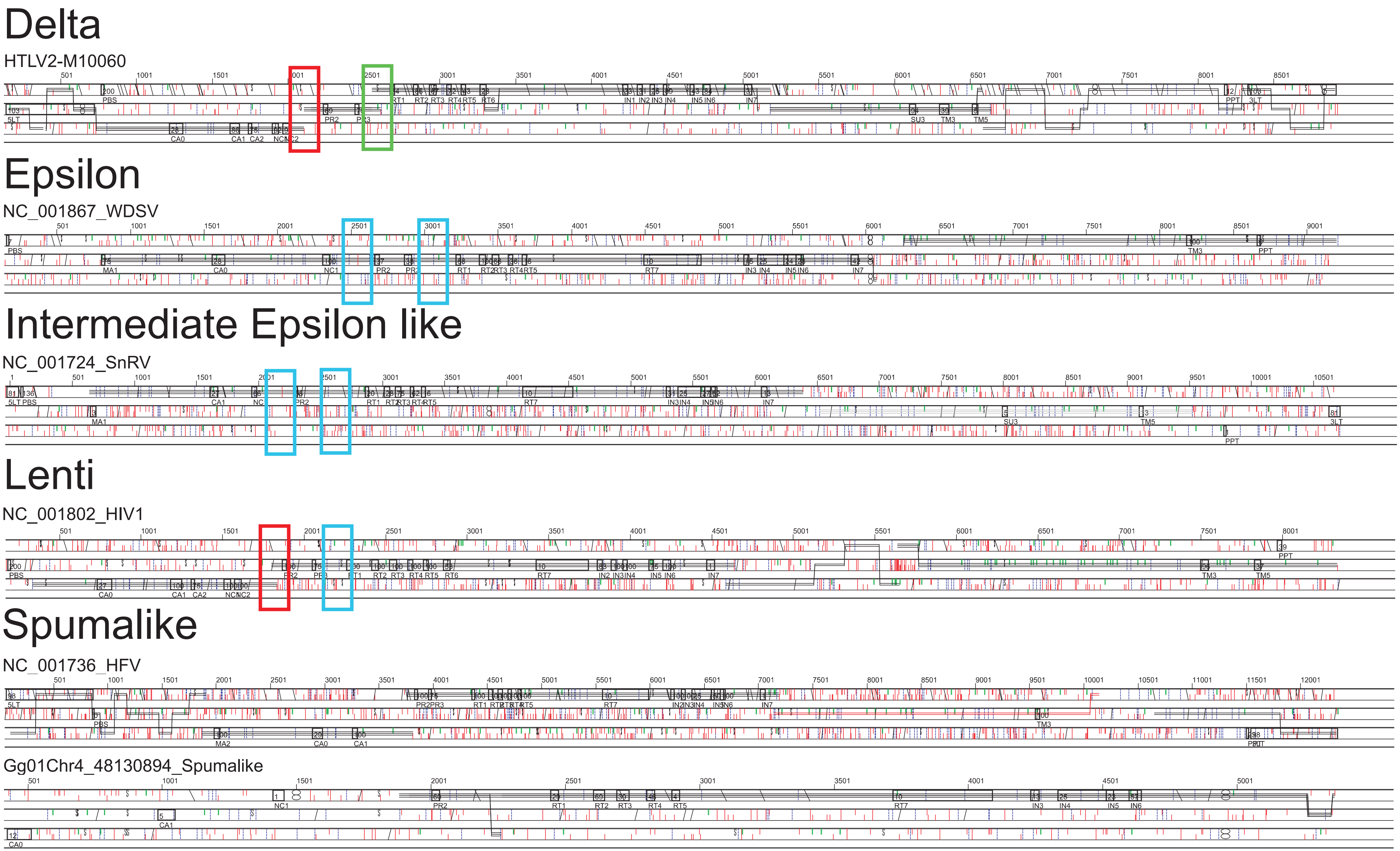
Delta
HTLV2-M10060
Epsilon
NC_001867_WDSV
Intermediate Epsilon like
NC_001724_SnRV
Lenti
NC_001802_HIV1
Spumalike
NC_001736_HFV
Gg01Chr4_48130894_Spumalike
