# Supplementary figures and images for: Use of Endogenous Retroviral Sequences (ERVs) and structural markers for retroviral phylogenetic inference and taxonomy
Source: Retrovirology. 2005 Aug 10;2:50. doi: 10.1186/1742-4690-2-50 (PMC1224870; doi:10.1186/1742-4690-2-50)

dUTPases  
Minimum Evolution (MEGA3.0)  
bootstraps >50% are shown

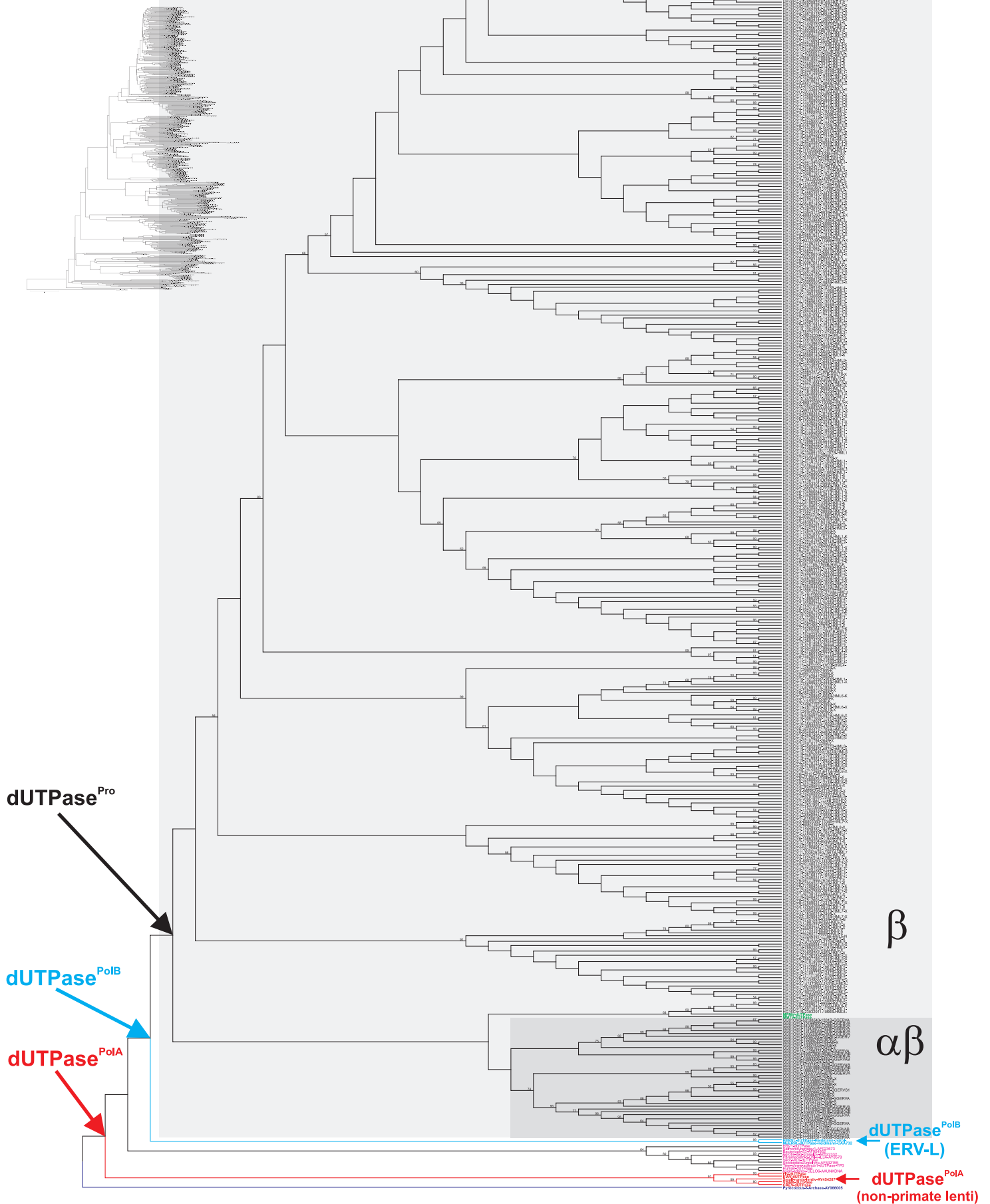

Supplement: Additional File 3 — dUTPase phylogenetic tree. Retroviral dUTPase acquisitions. Minimum Evolution (ME) tree (100 bootstraps). [file 1742-4690-2-50-S3.pdf]
