# Supplementary material for: Use of Endogenous Retroviral Sequences (ERVs) and structural markers for retroviral phylogenetic inference and taxonomy
Source: Retrovirology. 2005 Aug 10;2:50. doi: 10.1186/1742-4690-2-50 (PMC1224870; doi:10.1186/1742-4690-2-50)

WebLogo consensus of ERV dUTPase^{Pro}

RetroTector motifs are indicated over the consensus

(Alignment including 385 RetroTector predicted possible dUTPases.)

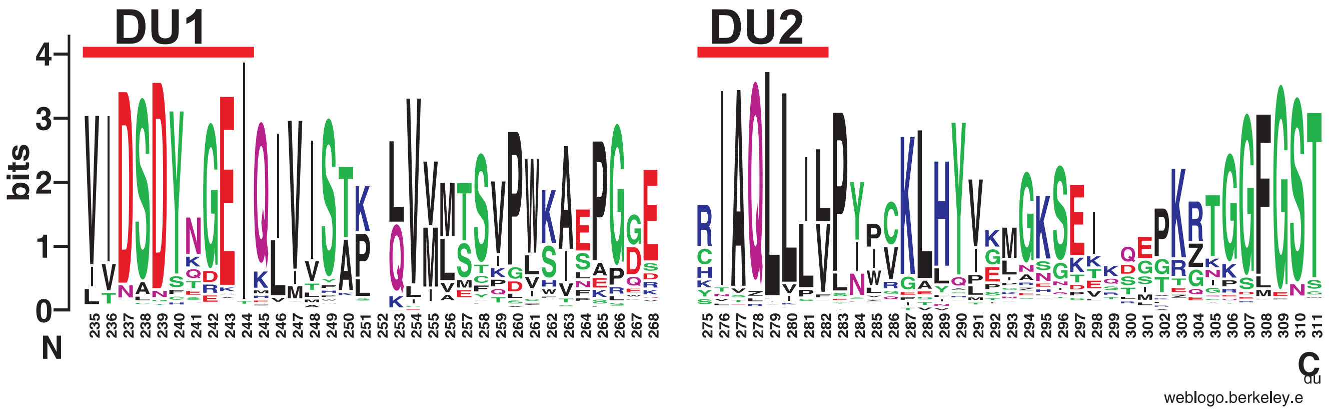

WebLogo consensus of ERV C-terminal Pro G-patch domain

(alignment including 78 RetroTector predicted possible G-patches)

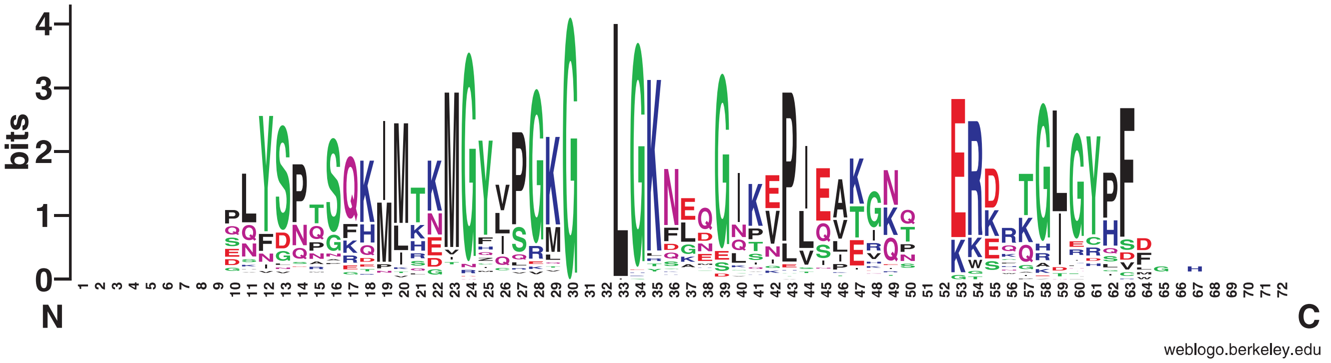

WebLogo consensus of ERV C-terminal Pol IN GPY/F domain

(alignment including 560 RetroTector predicted possible GPY/F motifs)

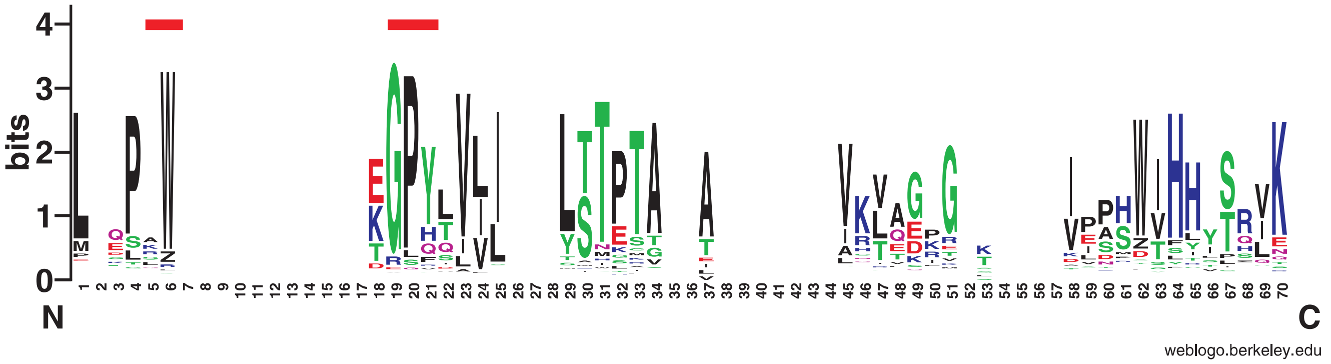

Supplement: Additional File 4 — Motif consensus validations. WebLogo consensus of the partial dUTPasePro, C-terminal Pro (G-patch) and Pol (GPY/F) motifs. [file 1742-4690-2-50-S4.pdf]
