# Supplementary material for: Use of Endogenous Retroviral Sequences (ERVs) and structural markers for retroviral phylogenetic inference and taxonomy
Source: Retrovirology. 2005 Aug 10;2:50. doi: 10.1186/1742-4690-2-50 (PMC1224870; doi:10.1186/1742-4690-2-50)

Selected RetroTector© RT and IN Motifs from the Pol alignment with WebLogo consensus

	RT1	RT4	RT5	IN2 and IN3 (HHCC)	IN5	IN6
**HERV-S**	YHFPVWPVRK	FHYIDDIMIL	GWTINESKVQ	LHRKLRHGGGGVNWCN-RS-VGAELSLPSQDILEACQKCSACTQA	SLAIESDRRTHFT---	HVSYNPQITGMIEQHS-ILK
**gg01-Chr4-48130894**	YNSPIWPVRK	FHYIDDLMLT	GWAINPQKVQ	LHRKLRHAGQKTMWAAAKA-WGLPIQLP--DIVQACQDCDACSRM	PEVIESDQGTHFT---	HLPYNPTGAGLIERYNGILK
**HERV-L**	FNSPIWPVQK	VHYIDDIMLI	GWEINPTKIQ	AHEQSGHGGRMEVMHG----LSNMDPLTKADLAMATAECPICQQE	PHSIASDQGTHFT---	HVPHHPEAAGLIERWNGLLK
**pt01-Chr5-53871501**	ZNTPLLPVKK	LQYVDDLLLA	GYRASAKKAQ	MHAWTHLSSKKLRLLIEK--TDFLIPRVGTLLEQVTLACKACQQV	PKVIGSDNGPAFVSQV	HCAYRPQSSGQVERMNRTIK
**BaEV**	WNTPLLPVKK	LQYVDDLLLA	GYRASAK-AQ	MHAWTHLGNRKLKLLIEK--TDFLIPRASTLIEQVTSACKVCQQV	PKVIGSDNGPAFVSQV	HCAYRPQSSGQVERMNRTIK
**MLV**	WNTPLLPVKK	LQYVDDLLLA	GYRASAK-AQ	LHQLTHLSFSKMKALLERSHSPYYMLNRDRTLKNITETCKACAQV	PQVLGTDNGPAFVSKV	HCAYRPQSSGQVERMNRTIK
**FLV**	WNTPLLPVKK	LQYVDDLLLA	GYRASAKKAQ	LHKLTHLSARKMKTLLEREETGFYLPNRDLHLRQVTESCRACAQI	PQVLGSDNGPAFISQV	HCAYRPQSSGQVERMNRSIK
**GaLV**	WNTPLLPVKK	LQYVDDLLVA	GYRVSAK-AQ	LHQLTHLGPEKLLQLVNR--TSLLIPNLQSAVREVTSQCQACAMT	PKVLGSDNGPAFVAQV	HCAYRPQSSGQVERMNRTIK
**PERV**	WNTPLLPVRK	LQYVDDLLLA	GYRASAKKAQ	IHRLTHLGTKHLQQLVRT--SPYHVLRLPGVADSVVKHCVPCQLV	PKVIGSDNGPAFVAQV	HCAYRPQSSGQVERMNRTIK
**pt01-Chr10r-17119458**	WNTPLLPVRK	LQYVDDLLIA	GYRASAKKAQ	IHRTTHLGARRMIDLIRR--SKLKIRHIDETASSIVTSCKVCQLN	PIQIGSDNGPAFVAKV	HCAYRPQSSGQVERMNRTLK
**HERV-T**	WNTPFLPVQK	LQYVDDILIT	GYGVSAKKAQ	LHSTTHLG-TKLAQLLRS---CFKIPHLQSLTDQAVLWCTACAQV	PAAIGSDNGPAFTSSI	HCAYQPQSSGQVECMNHTLK
**ERV-3**	WNTPLLLVQK	LQYIDNLLLA	GYRVSKKKAQ	LH-HELVGKTALETLLGR---HFYVPRLTAITQAVCEQYLTCAQN	SVTLGSDNRLAFLAEI	H-------------------
**HERV-E**	WNTPLLPVPK	LQYVDDLLLG	GIRCPRKKAQ	VHKTTHLGQESLEKLLGW---YFYISHLSALAKTVTQRCVTCRQH	PLRIGSDNGLVFVADL	HAAYQPQSSGKVERMNRTIK
**gg01-ChrU-126703652**	YSITILPVRK	LQYVDNLLIA	GYRVSPQZAQ	EHRKTHWGTEALYKYLVZ---QVAAZNLSTTTRQVTQQCEVCLQN	LGVISSDRCLHFVAKI	HTPYRPQLSGQVEKMNHZLK
**HERV-ADP**	YNTPILPVKS	LQYVDYLLIS	GLZVSKNKMQ	LHKGSHWGPQALCDAILR---NYVCIGIZTLTKQVCGSCVICQSL	AENIDSDNGSHFTSRV	RTPWHPPSSGKVERMNQTFK
**gg01-chrU-49656081**	FNTPILPVRK	LQYVDDLLLS	GLRVSKNKLQ	LHQGSHWGVQAMCDAVLR---KYVSAGIYTLAKQVCRECAVCQRV	VEHIDSHQGSHFTSQV	HTPWHPSSSGKVERMNQTIK
**HERV-I**	YNTPILPVKK	LQYVDDILIS	GLRVSKGKLQ	YLR-THWGSQAMCDAVLR---VYGCIRIYTLAKQVTDSCLICKKT	IQSTDSDNGTHFTAYV	HIPWHLSSSRRVERMNQTLK
**HERV-Hconsensus**	YNSPILPVQK	IQYIDDLLLC	GYRVSPSKAQ	FHNLFHVGYKPLARLLEP---LISFPSWKSILKEITSQCSICYST	PTSIQSDNGPAFTSQI	HIPYHPQSSGKVERTNGLLK
**HERVH-RGH2**	----------	IQYIGDVLLV	GY---PSKAQ	FHNLFHVGYKPLACLLGP---LISFPSWKSILKEITSQCSICYSA	PFSIQSDNGPAFTSK-	HIPZHPQSSGKVKPTNGLLK
**HERVH-RTVLH2**	YHSPILPVLK	----------	--------AQ	---------------LRT---LISFPSWKSIPKEITSQCSICYST	PTSIQSHSRPAFIGEI	YIPYSPQSSGKVEQTNSLLK
**HERV-Fc1**	CNTPILPIRK	LLYVDDLLLC	GYRVTPHKVQ	VHKTLLIGPKLLHQFLEP---IFLCPGLQSLIHQVHQTCAVCSTV	PRSLQSDNGPAFVSKI	HIPYRPQSSGKVERANSLLK
**HERV-Fc2**	CNTPILPVRK	IQYVDDLLLC	GYRVTPSKAQ	MHRSLHIGPKALYNFLEP---IIYHPSLYSLLKQIHQECHVCTVA	PDSP-SQDGSLCFQEI	DHACNPRTLR--GRGGQITZ
**HERV-W**	CSTPILGVQK	SFGVDDLLLA	GYMVSKP-AQ	LHQTFHLGIDSTHQMAKS---LFTGPGLFKTIKQIVRACEVCQRN	PRGLQSDNSPAFQATV	CAZ-RPQSSGKVEKMNETLK
**HERV-FRD**	YNTPILTVKK	LQYMDDLLIC	RYKVSKAKTQ	FYNFFHLAKDSLGQICKZ---VFSGKGLNKTIQQVCQAYTLCTIN	PRSLQSDNGPSFISZV	FTLPGDQPSGKVERTKETLE
**MER4like**	CNTLTLGVQK	LQYVDDIPLG	RYKVSKSKSZ	LHQAFPLGQDKTYLLAQR---LFTGKNLLKMAKQVANALKTALKI	PWYLQTDYGSCFKAVV	FSAWRPQFSEKVEKTSDIIR
**WDSV**	CNTPIFPIKK	CIYMDDVLIA	GHKVSKKKLQ	IHGVSHTHKGGIMSYFSKF---WTHPKASQTIDLILGHCQICLKH	PDQIDSDQGTHFTAKI	HCPGHPRSSGIVERTNRTLK
**hg15-chr3-152465283**	CNTPILAVKK	IQYIDEYMLP	GHKASVEKLK	LHSTTHHGTDKMIQLEKN-----TGGVTSKTAKMVYNQCLTCQSN	PEKSSSDRGTHFIGQV	HCPYHPHSSEKVERTNVILK
**Xen1**	CNTPLFPVKE	LQYVDDLLLC	GCKASKEKLQ	AHGPTHVSKNLMNNLISKL---YFAPGITTLTRNYTAACTICAQC	PEVIESDQGPVFTASV	HTPYHPQSSGKVERMNGTLK
**ALV**	WNTPVFVIRK	LHYMDDLLLA	GFTISPDKIQ	LHTALHIGPRALSKAC-N----IS-MQQAREVVQTCPHCN--SAP	PKAIKTDNGSCFT---	GIPGNSQGQAMVERANRLLK
**gg01-chr1-156168845**	WNTPIFVIRK	VHYMDDLLLA	GFTISQEKVQ	LDSISQKGPYWYSRIN-R----SDPFTDVFPRDRTHITGE--KSI	GIPVKLVGGPCYLGQL	RMRTKRDLQQLSPNCS----
**MPMV**	WNTPIFVIKK	IHYMDDILIA	GLHIAPEKVQ	AHTLHHLNAQTLRLMF-N----IP-REQARQIVKQCPICVTYLPV	PKQIKTDNGPGYT---	GIPYNPQGQGIVERAHLSLK
**JSRV**	----------	VHYMDDILLA	GLVIADEKIQ	SHDLHHQNSHSLRLQF-K----IS-REAARQIVKSCSTCPQFFVL	PQTLKTDNGPGYT---	GIPYNPQGQGIVERAHQRIK
**MMTV**	WNTPVFVIKK	VHYMDDILLA	GLVVSTEKIQ	SHALHHQNAAALRFQF-H----IT-REQAREIVKLCPNCPDWGHA	PQKIKTDNAPAYV---	GIPYNPQGQAIVERTHQNIK
**HML1**	WNSPVFVIQK	IHYTDDILCA	GLVIAPEKIQ	FHALTHVNAAGLRNKF-P----LT-WKQAKIIVRHCPTCQVLILQ	PEKLKTDNGPGYT---	GIPYNSRGQALVERAKKTLK
**HML2**	WNSPVFVIQK	IHYIDDILCA	GLAIASDKIQ	LHALTHVNAAGLKNKF-D----VT-WKLAKDIVQHCTQCQVLHLP	PEKIKTDNGPGYC---	GIPYNSQGQAIVERTNRTLK
**HML3**	WHSPVFVIKK	IHYMDDILCA	GLIIAPDKIQ	FHNSTHVNASGLQCRY-S----IT-WKEAKAIIQZCPTCQMVHSS	PASIKTDNATGYT---	GIPYNSQGQAIVERMNLSLK
**HML4**	WNSPVFIIKK	IHYMDDILCA	GLITAPDKIQ	FHNLTHVNASGIKHRY-S----IT-WKEAKAIIQZCPTCQMVHSS	SASIKTDNASGYT---	GIPYNSQGQAIVEZMHLSLK
**HML7**	WNSPVFVVQK	LHYMDDILIA	GLNIAPDKIQ	FHQLTHTNVSGLKHKY-S----LS-WKQPKQIVQHCSQCQVLVLP	PGELKTDNGPAYC---	GIPYNPQGQAIVEKSNRTLK
**HML8**	WNSPVFLVKK	LHYIDDILIA	GLHITQGTIQ	FHNLTHVNAAGLKDKF-A----LS-WKQAKFIVHSCPQCQVFVLP	PYQIKIDYTAGYV---	GIAYNPQGQAVVERANRTLK
**HML6**	WNSPVFVIPK	IHFMDDILLA	NLKIAPEKVQ	SHALLHQNTSALTRMF-H----LP-XSQAXAIVQACPTCQHVPGV	PKQLKTDNGPAYV---	GIPYNPRGQGIIERAHQTLQ
**HML9**	-NSPVFVIPK	IHYMDDILLA	NLKIAPEKVQ	SHAFLHQNTSALTRMF-H----LP-CSQAZAIAQAYPTCQPVPGV	PTQZKTDNGPAYV---	EIPYNPQGQGIIEWAHQTLQ
**HML5**	WNSPIFIIPK	IHFMDDILLA	GLIIAPEKVQ	SHQFFHQGWRNLSKQF-Q----LT-QRLAKQIILQCPDCQLIGTS	PTKIKTDNGLAYA---	GILYNPQGQAIVDHTHSTLK
**HML10**	WNSPVFVIKK	---------A	ELLIAPDKIQ	SHRLFHQNWRNLSKZF-Q----LT-QRLAKQIIPQCPDYQLTGTY	HKKIKVKKRLRQK---	--VFS--KKIIKHKKLRHVK
**Python-molurus**	YNTPVFVIKK	YHYMDGILVA	GLHIAPDKVQ	SHSYFHQNKKALIKQF-K----IS-ATEATAILQQCPTCS-KQAH	PKELKTDNGPGYT---	GIPYNSKGQAIIGRANQTLK
**gg01-chr7-5733782**	WNTPIFIIKK	YHYTDDILIA	GLVIAPEKVQ	AHATFHQNARGLHKLY-K----IS-LNEARGIVRACPSCS-NFSP	PKTFKTDNGPAYT---	GVLNSPAGQAIVERANETLK
**gg01-ChrU-163504869**	YNTPILSVKK	LQYVDDFLIA	AICQTPEPRT	AHATFHQNARGLHKLY-K----IS-LNEARGIVRACPSCS-NFGP	PKTLKTDNGPAYT---	GVPNSPTGQAIVERANQTLK
**HIV-1**	YNTPVFAIKK	YQYMDDLYVG	GLTTPDKKHQ	EHEKYHSNWRAMASDF-N----LP-PVVAKEIVASCDKCQ--LKG	VKTIHTDNGSNFT---	GIPYNPQSQGVVESMNKELK
**HIV-2**	YNTPTFAIKK	IQYMDDILIA	GFSTPDEKFQ	EHEKYHSIIKELTHKF-G----IP-LLVARQIVNSCAQCQ--QKG	ITHLHTDNGPNFT---	GVPYNPQSQGVVEAMNHHLK
**Visna**	CNTPIFCIKK	GIYMDDIYIG	GFMLPEDKRQ	EHNKWHQDAVSLHLEF-G----IP-RTAAEDIVQQCDVCQENKMP	PKSLQSDNGPAFV---	GIPWNPQSQALVERTHQTLK
**EIAV**	YNSPIFVIKK	YQYMDDLFMG	GFETPDDKLQ	EHENWHTSPKILARNY-K----IP-LTVAKQITQECPHCT--KQG	PKSLHTDNGTNFV---	GIPYHPESQGIVERANRTLK
**HTLV-1**	GNNPVFPVKK	LQYMDDILLA	GLPVSENKTQ	LHSFTHCGQTALTLQG-------ATTTEASNILRSCHACRKNNPQ	PSYINTDNGPAYI---	HVPYNPTSSGLVERSNGILK
**HTLV-2**	GNNPVFPVKK	VQYMDDILLA	GLPISQEKTQ	LHGLTHCNQRALVSFG-------ATPREAKSLVQTCHTCQTINSQ	PLHINTDNGPAFL---	HIPYNPTSSGLVERTNGVIK
**BLV**	GNNPVFPVRK	VSYMDDILIA	GFQVASEKTR	WHKLTHCNSRALSRWPNP----RISAWDPRSPATLCETCQRLNPT	PKKLNTDQGANYT---	HVPYNPTSSGLVERTNGLLK
**HSRV**	MNTPVYPVPK	QVYVDDIYLS	GYVVSLKKSE	FLNCPHS-----LLPLT------------SNAGLLKHCLMAGKW	EAFTYS---VAVSQ--	VZ-----------------N
**HFV**	MNTPVYPVPK	QVYVDDIYLS	GYVVSLKKSE	AHNLAHTGREATLLKIANLYWWPNMRKDVVKQLGRCQQCLIT-NA	PKVIHSDQGAAFTS--	STPYHPQSGSKVERKNSDIK
**HERVL66**	----------	----------	----------	----------------------RISLPQDAVQTILSQCTTCQQL	PIQIQTDNGSHFKG--	HIPYHLQAAGLTERMNELLN
**SnRV**	VNSAIWPVGK	LQYVDDILLM	GVKINPKKSH	HHLYGHPSEESLRKVLTKR---FVWEDMGQHCKEITNTCLTCAKY	PYSIQSDNGTAFT---	GAIYHPQSQGKVERKHRLLK
**gg01-chr4-77338201**	WNTPIFVIQK	YHYMDDILFA	SLVVAPEKVQ	QSQLQPSGAVAVRTVGGG-----GILGEPEQFTGESFWNALALYE	WITLANKMGQDHICLA	DVLLVPRQLSLVTVLPQMIM
**HERVL40**	XNSLVXPVXK	FHYIDXVLLV	KLQKHSGTCL	IHHPTGHGNTSTIXDWAQSKGPYVSDAEATTACQTCDSCQKLPVX	PDYLQSDNGAPFIT--	HAPYYPRASGIVERWNGLLI
**Gypsy**	WSAPVWVVPK	LFYLDDIIIF	NFKLQPSKSE	QDKLYSEIKEKLSTNTQQRIEKLNTSRVEPTTVQPNSTIFRKENR	PTVLKNYTTPSIPZDK	ZHPFHATKEVLLTGZVVZSK
**HERVL74**	FNSPILPVLK	ZHYIDDILLQ	EATYSPFTIL	VTKAHFASQNTQCWALEKG---IQWNFHLPXRPQIAGXIERIMVS	SYICGPNNVSSYLY--	HKGWRPDRRHILSWDPLTID
**gg01-chrU-52190725**	FNTPILPVRK	IQYVDDLLLA	SYNYRIHXXX	SWNSFGKLCGSLLSQHIMVSLYLNRRRQQDLRRVLRSDLCDFRIN	PZEIPNKTFDCMHFAL	LVTFLVTSLNAPRKKSETME
**gg01-chr7-7163462**	LNGGRYTEAH	LKYVDHLLLS	GLRVPRNKSQ	ZNPMTLRVPTLLRFFLNLS---VFPASQHSLVLEETSSLARMERN	YRPVTGKEHIAFN---	IAGNCZREGGEKKKQKETTR

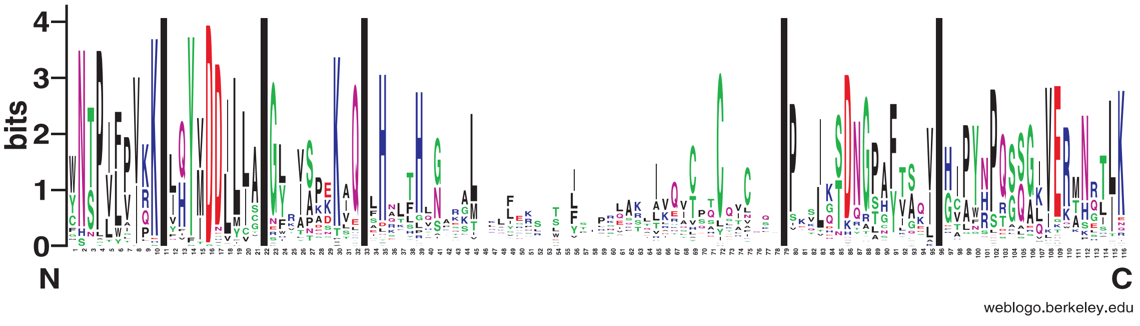

Supplement: Additional File 5 — Putein validations. Validation of puteins from Pol alignment. Excised parts of RT and IN are shown. [file 1742-4690-2-50-S5.pdf]
