# Supplementary material for: Use of Endogenous Retroviral Sequences (ERVs) and structural markers for retroviral phylogenetic inference and taxonomy
Source: Retrovirology. 2005 Aug 10;2:50. doi: 10.1186/1742-4690-2-50 (PMC1224870; doi:10.1186/1742-4690-2-50)
Supplement: Additional File 6 — Pol FASTA sequences. Pol FASTA sequences. [file 1742-4690-2-50-S6.pdf]

Pol FASTA sequences

```
>HERV-S
SGGGHFAWSGLKHHGQRIQTLSSCNKASNAWIYTPPAPVLPQPQRVTSTHQYHLPGGHTE
ITKTIKKLEELQIVSGTHRPYHFPVWPVRKPDGTWRMTVDYRELNEATPPLHAAVPSIMD
FMEHLTTELGQYHFVVDLANAFFSNHIAPESQEQFTFTWEGSQWTFTMLPQGYVHSPTIC
HGFVAMDLAAWNCPKGVSLFHYIDDIMILADLEGAGPLLQQQLAACGWTINESKVQGPGL
SAKLLGVIWLDKTKAIPEAITDKIQAYPZPTTVRQLQTFVGFLGYZQALVSHLAQMIKPL
YQLTKQGATWNWVDEANSLSGHQAGYSLDMSLTSDZSGRPFEFDVHVATDGFGWGLWQCI
EHFRMPVGFCPTYGRELSYGNLLEKQLAAAYAALQACENLTGWPTVVMQMTYPIAEWVGS
WVITPQTGRVQTSTLAKWGAYLEQQSTLNTSPLAAELPEVLGHVVLRQGKAMGPEAPLTM
SHHHLRKGALSFLMDIVYRWVQPSATAAWTAVAVQPTTHTIWFYTGCGQSSQWAELRVVW
MVITKEESPGNLYQZLGSLLRVNLVVDYLEVIELACGHRPIWGQTMWQDIWEMGHQVSGH
MPLTTPSNDEANALTKVQWLELAPTZDVALWLHRKLRHGGGGVNWCNRSVGAELSLPSQD
ILEACQKCSACTQAYPRYRQLPSVTQQVMVRQMPLTTWQIDYIGPLPKFQGYTHALMAVD
MATGLLFTYPCRVADQQHTIZTLEHVCALYSHSLAIESDRRTHFTGQQVQQWAEQIDIQZ
GFHVSYNPQITGMIEQHSILKNGLHLHVTHPSLWGWSSRLDLVFQILNEWPRKGGPAPVE
ALLHWATAPIQHRYRPRMTSSDQVWGQMVICCCLPQCPRRQENRKPGVDHGPSKHTIADL
AIIAPCGEDLQCDLHVTPRVFTVWPPQLTIHRGMAREGTLPGDIGTVCVTQLPCDFSMST
RHKITTESZZDVVPSPREEAIGDCIVIQKKKWARILPEGRDLPLLVPVPALPFWPQVDML
QQHRGLGPHLCZGDQCLQLLS
>gg01-Chr4-48130894
PICLPQPRRITNTKQYRLPGGQQEITKTVQELERVGIIRPAHSPYNSPIWPVRKPDGTWR
MTVDYRELNKVTPPIHAAVPNIASLMDTLSREIETYHCVLDLANAFFSIPIAKESQDQFA
FTWEGRQWTFQVLPQGYVHSPTFCHNLVASDLANWNKPSTVKMFHYIDDLMLTSDSIEAL
EKTVPSLITYLQEKGWAINPQKVQGPGLSVKFLGVVWSGKTKVLPSAIIDKMQAFPVPTK
PKQLQEFLGILGYWRSFIPHLAQLLKPLYRLTKKGQVWDWGRTEQEAFQQAKIAVKQAQA
LGIFDPTLPAELDVHVTQEGFGWGLWQRQSSVRIPIGFWSQIWHGAEERYSMVEKQLLAT
YSALQAVEPITQTAEVIVKTTLPIQGWVKDLTHIPKTGVAQSQTVARWVAYLSQRSRLSS
SPLKEELQKILGPVTYYSETPEEIVVTCPEKSPVQEGKYPIPEDAWYTDGSSRGNPSKWR
AVAYHPSTETIWFEEGDGQSSQWAELRAVWMVITQEPGNSALNICTDSWAVYRGLTLWIA
QWATQDWTIHARPIWGKDMWVDIWNVVRHRTVRAYHVSGHQPLQSPGNDEADTLARVRWL
GSTPSEDIAHWLHRKLRHAGQKTMWAAAKAWGLPIQLPDIVQACQDCDACSRMRPRPLPE
TTAHLARGHNPLQRWQIDYIGPLPRSEGARYALTCVDTASGLMQAYPVAKANQAYTIKAL
TRLMASYGTPEVIESDQGTHFTGATVQKWAEDNNIEWRFHLPYNPTGAGLIERYNGILKA
ALKADSQSLQGWTKRLYETLRDLNERPRDGRPSALKMLQTTWASPLRIQITSKDTSLKPQ
VGTMNNLLLPAPDDLEPGRHKVKWPWKVQAGPKWCGLLAPWGRLLEVGGSVNPSVIGVWP
TEVIVDTPVFIARGT
>HERV-L
IVNQKQYRIPGGIAEISATIKDLKDAGVVIPTTSPFNSPIWPVQKTDGSWRMTVDYRKLN
QVVTPIAAAVPDVVSLLEQINTSPGTWYAAIDLQAFFSIPVHKAHQKQFAFSWQGGQQYTF
TVLPQGYINSPALCHNLFGETLIAFLPQDITLVHYIDDIMLIGSSEQEVANTLDLLVRHL
RARGWEINPTKIQGPSTSVKFLGVQWCGACRDIPSKVKDKLLHLAPPNQERGTTPSGPIW
ILEATHSSLGVLLRPIYRVTRKAASFEWGPEQEKALQQVQAAVQAALPLGPYDPADPMVL
EVSVADRDAVWSLWQAPIGESQRRPLGFWSKALPSSADNYSPFERQLLACYWALVETERL
TMGHQVTMRPELPIMNWVLSDPSSHKVGHAQQHSIINGSGIYVIGLAGLGTSKLHEEVAQ
MPMVSTPATLPSLPQLHMASWGFPMILTEEEKTRAWFTDGSARYAGTTRKWTAAALQPLS
RTSLKDVVKGNLPSGQLRAVHLVVHFAWKEKWPDVRLYTDSWAVANVWLDGGTWKKHDWK
IGDKEIWGRGMWMDLSEWSKTVKIFVSHVSAHQRVTSAEEDFNNQVDRMTHSVDTTQPLS
PATPVIAQWAHEQSGHGGRMEVMHGLSNMDPLTKADLAMATAECPICQQETNTEPSIGTI
PRGISQLWWQVDYIGPLPSWKGQRFVLTGIDTYSGYGFAYPARNASAKTTIRGLTECLIH
RHGIPHSIASDQGTHFTAKEVWQWAHAHGIHWSYHVPHHPEAAGLIERWNGLLKSQLQCQ
LGDNTLQGWGKVLQKAVYALNQRPIYGTVSPIARIHGSRNQGVEVEVAPLTITPSDPLAI
FASCSRDITFCWPRGLSSRGRNAPPGDTTTIPLNWKLRLPPGHFGLLLPLSQQAKKGVTV
LAGVIDPLS
>pt01-Chr5-53871501
TPILTSPSDNWLQEFPQAWAETGGLGLAKFQAPIIVDLKPTAVPVSIRQYPMSZEARMGI
QQHINKFLELGVLRPCRSPZNTPLLPVKKPGTQDYRPIQDLREINKRTMDIHPTVPNPYN
LLSSLRPDHNWCTVLDLKDAFFCLPVAPQSQELFAFEWRDPEKGISGQLTWTRLPQGFKN
SPTLFDEALHRDLTDFHTQHPDLTLLQYVDDLLLAAPTKEACLQGTRHLLQELGDKGYRA
```

SAKKAQICQTKVTYLGYILSEGKRWLTPGQIETVARIPPPRNPKEVREFLGAAGFCRLWI
PGFAELAAPLYALTKRSNPFIWLEEHQQAFEALKKALLSAPALGLPDTSKPFTLFVDERR
GIAKGVLTQKLGPWKRPVAYLSKKLDPVAARWPPCLRIMAATAMLVKDSAKLTLGQPLTV
ITPHALEAIVRQPPDHWITNARLTHYQALLLDADRVNFGPPVTLNPATLLPVPEDPLSPH
DCRQVLAETHGTREDLQDYELPDADHTWYTDGSSFMDAGTRKVGAAVVDGHATIWAQALP
PGTSAKAELIALTKALELSQGKKANIYTDSRYAFATAHTHRSIYERRGLLTSERKEIKNK
AEIIALLKALFLPKKVAIIHCPGHQKGHDPVAQGNRQADQAAKQAARVETLTLVSETRKA
DQMPSPTSYTYTPEDQKEAVALGATENQETKNWEKDGKTVLPQKEAMAMLQQMHAWTHLS
SKKLRLLIEKTDFLIPRVGTLLEQVTLACKACQQVNAGATRVPARIKAWGNRPRTLLEVD
FTEVKPHCGGYKYLLVFVDTFSGZVEAFPTRQETAHIVAKKILDRIFPRFGLPKVIGSDN
GPAFVSQVSQGLARILGINWKLHCAYRPQSSGQVERMNRTIKETLTKLTLETGLKDWRCL
LSLALLRARNMPNRFRLTPYEILYGGPPPLSTLLDSYSPSDLKTDLQARLKGLQAVQAQI
WTPLAELYQPGHPQSSHPFQVGDSVYVRRHRSQGLEPRWKGPYIILLTTPTAVKVDGVAA
WIHASHVKAAPEVSGPASPERWRLRRSGDPLKIRLSRV
>BaEV
WNTPLLPVKKPGTQDYRPVQDLREINKRTDIHPTVPNPYNLLSTLKPDYSWYTVLDLKDA
FFCLPLAPQSQELFAFEWKDPERISGQLTWTRLPQGFKNSPTLFDEALHRDLTDFRTQHP
EVTLLQYVDDLLLAAPTKKACTQGTRHLQELGEKGYRASAKAQICQTKVTYLGYILSEGK
RWLTPGRIETVARIPPPRNPREVREFLGTAGFCRLWIPGFAELAAPLYALTKESTPFTWQ
TEHQLAFEALKKALLSAPALGLPDTSKFTLFLDERQGIAKGVLTQLGPWKRPVAYLSKKL
DPAAGWPPCLRIMAATAMLVKDSAKLTLGQPLTVITPHTEAIVRQPPDRWITNARLTHYQ
ALLLDTDRVQFGPPVTLNPATLPVPENQPSPHCRQVLAETHGTREDLKDQELPDADHTWY
TDGSSYLDSGTRRAGAAVVDGHNTIWAQSLPPGTSAQKAELIALTKALELSKGKKANIYT
DSYAFATAHTHGSIYERRGLLTSEGKEINKAEIIALLKALFLPQEVAIIHCPGHQKGQDP
VAVNRQADRVARQAAMAEVLTLATEPDNTSHIIEHTYTSEDQEEARAIGATENKDTRNWE
KEGKIVLPQKEALAMIQQMHAWTHLGNRKLKLLIEKTDFLIPRASTLIEQVTSACKVCQQ
VNAGATRVPAGKRTRGNRPGVYWEIDFTEVKPHYAGYKYLLVFDTFSGWVEAFPTRQETA
HIVAKKILEEIFPRFGLPKVIGSDNGPAFVSQVSQGLARILGNWKLHCAYRPQSSGQVER
MNRTIKETLTKLTLETGLKDWRRLLSLALLRARNTNRFGLTPYEILYGGPPPLSTLLNSF
SPSNSKTDLQARLKGLQVQAQIWAPLAELYRPGHRTSHPFQVGDSVYVRRHRSQGLEPRW
KGPYIVLLTTPTAIKVDGIATWIHASHAKAAPGTGPTSSGTWRLRRSEDPLKIRLSRT
>MLV
AQMGPMGQPLQVLTLNIEDEHRLHETSKEPDVSLGSTWLSDFPQAWAETGGGLAVRQAPL
IIPLKATSTPVSIKQYPMSQEARLGIKPIQRLLDQGILVPCQSPWNTPLLPVKKPGTNDY
RPVQDLREVNKRVDIHPTVPNPYNLLSGLPPSHQWYTVLDLKDAFFCLRLHPTSQPLFAF
EWRDPEMISGQLTWTRLPQGFKNSPTLFDEALHRDLADFRIQHPDLILLQYVDDLLLAAT
SELDCQQGTRALQTLGNLGYRASAKAQICQKQVKYLGYLLKEGQRWLTEARKETVMGQPT
PKTPRQLREFLGTAGFCRLWIPGFAEMAAPLYPLTKTGTLFNWGPDQQKAYQEIKQALLT
APALGLPDLTKFELFVDEKQGYAKGVLTQLGPWRRPVAYLSKKLDPAAGWPPCLRMVAAI
AVLTKDAGKLTMGQPLVILAPHAEALVKQPPDRWLSNARMTHYQALLLDTDRVQFGPVVA
LNPATLPLPEEGLQHCLDILAEAHGTRPDLTDQPLPDADHTWYTDGSSLLQEGQRKAGAA
VTTETEVIWAKALPAGTSAQRAELIALTQALKMAEGKKLNVYTDSYAFATAHIHGEIYRR
RGLLTSEGKEINKDEILALLKALFLPKRLSIIHCPGHQKGHSAEARNRMADQAARKAAIT
ETPDTSTLLIENSSPYSEHFHYTVTDIKDLTKLGAIYDKTKKYWVYQGKPVMPDQFTFEL
LDFLHQLTHLSFSKMKALLERSHSPYYMLNRDRTLKNITETCKACAQVNASKSAVKQGTR
VRGHRPGTHWEIDFTEIKPGLYGYKYLLVFDTFSGWIEAFPTKKETAKVVTKKLLEEIFP
RFGMPQVLGTDNGPAFVSKVSQTVADLLGDWKLHCAYRPQSSGQVERMNRTIKETLTKLT
LATGSRDWVLLLPLALYRARNTGPHGLTPYEILYGAPPPLVNFPDPDMTRVTNSPSLQAH
LQALYVQHEVWRPLAAAYQEQLDRPVVPHPYRVGDTVWVRRHQTKNLEPRWKGPYTVLLT
TPTALKVDGIAAWIHAAHVKAADPGGPSSRLTWRVQRSQNPLKIRLTRE
>FLV
TLQLEEEYRLFEPESTQKQEMDIWLKNFPQAWAETGGMGTAHCQAPVLIQLKATATPISI
RQYPMPHEAYQGIKPHIRRMLDQGILKPCQSPWNTPLLPVKKPGTEDYRPVQDLREVNKR
VEDIHPTVPNPYNLLSTLPPSHPWYTVLDLKDAFFCLRLHSESQLLFAFEWRDPEIGLSG
QLTWTRLPQGFKNSPTLFDEALHSDLADFRVRYPALVLLQYVDDLLLAAATRTECLEGTK
ALLETLGNKGYRASAKKAQICLQEVTYLGYSLKDGQRWLTKARKEAILSIPVPKNSRQVR
EFLGTAGYCRLWIPGFAELAAPLYPLTRPGTLFQWGTEQQLAFEDIKKALLSSPALGLPD
ITKPFELFIDENSGFAKGVLVQKLGPWKRPVAYLSKKLDTVASGWPPCLRMVAAIAILVK
DAGKLTLGQPLTILTSHPVEALVRQPPNKWLSNARMTHYQAMLLDAERVHFGPTVSLNPA
TLLPLPSGGNHHDCLQILAETHGTRPDLTDQPLPDADLTWYTDGSSFIRNGEREAGAAVT
TESEVIWAAPLPPGTSAQRAELIALTQALKMAEGKKLTVYTDSRYAFATTHVHGEIYRRR

GLLTSEGKEIKNKNEILALLEALFLPKRLSIIHCPGHQKGDSPQAKGNRLADDTAKKAAT
ETHSSLTVLPTELIEGPKRPPWEYDDSDLDLVQKLEAHYEPKRGTWEYRGKTIMPEKYAK
ELISHLHKLTHLSARKMKTLLEREETGFYLPNRDLHLRQVTESCRACAQINAGKIKFGPD
VRARGRRPGTHWEVDFTEIKPGMYGYKYLLVFIDTFSGWAEAYPAKHETAKVVAKKLLEE
IFPRYGIPQVLGSDNGPAFISQVSQSVATLLGINWKLHCAYRPQSSGQVERMNRSIKETL
TKLTLETGSKDWVLLLPLVLYRVRNTPGPHGLTPFEILYGAPPPMAHFFDTDISSFATSP
TMQAHLRALQLVQEEIQRPLAAAYREKLETPVVPHPFKPGDSVWVRRHQTKNLEPRWKGP
HIVLLTTPTALKVDGVAAWIHASHVKAAGPTTNQDLSDSPSSDDPSRWKVQRTQNPLKIR
LSRGT
>GaLV
GIRPIQKFLDLGVLVPCRSPWNTPLLPVKKPGTNDYRPVQDLREINKRVDIHPTVPNPYN
LLSSLPPSYTWYSVLDLKDAFFCLRLHPNSQPLFAFEWKDPEKNTGQLTWTRLPQGFKNS
PTLFDEALHRDLAPFRALNPQVVLLQYVDDLLVAAPTYEDCKKGTQKLQELSKLGYRVSA
KAQLCQREVTYLGYLLKEGKRWLTPARKATVMKIPVPTTPRQVREFLGTAGFCRLWIPGF
ASLAAPLYPLTKESIPFIWTEEHQQAFDHIKKALLSAPALALPDLTKFTLYIDERAGVAR
GVLTQLGPWRRPVAYLSKKLDPASGWPTCLKAVAAVALLLKDADKLTLGQNVTVIASHSE
SIVRQPPDRWMTNARMTHYQSLLLNERVSFAPPAVLNPATLPVESEATPVHCSEILAEET
GTRRDLEDQPLPGVPTWYTDGSSFITEGKRRAGAPIVDGKRTVWASSLPEGTSAQKAELV
ALTQALRLAEGKNINIYTDSYAFATAHIHGAIYKQRGLLTSAGKDINKEEILALLEAIHL
PRRVAIIHCPGHQRGSNPVATNRRADEAAKQAALSTRVLAGTTKPQEPIEPAQEKTRPRE
LTPDRGKEFIKRLHQLTHLGPEKLLQLVNRTSLLIPNLQSAVREVTSQCQACAMTNAVTT
YRETGKRQRGDRPGVYWEVDFTEIKPGRYGNKYLLVFDTFSGWVEAFPTKTETALIVCKK
ILEEILPRFGIPKVLGSDNGPAFVAQVSQGLATQLGNWKLHCAYRPQSSGQVERMNRTIK
ETLTKLALETGGKDWVTLLPLALLRARNTGRFGLTPYEILYGGPPPILESGETLGPDDRF
LPVLFTHLKALEVRTQIWDQIKEVYKPGTVTIPHPFQVGDQVLVRRHRPSSLEPRWKGPY
LVLLTTPTAVKVDGIAAWVHASHLKPAPPSPDESWELEKTDHPLKLRIRRRRDESA
>PERV
GRRGSDPLPEPRVTLKVEGQPVEFLVDTGAKHSVLLQPLGKLKDKKSWVMGATGQQQYPW
TTRRTVDLGVGRVTHSFLVIPECPAPLLGRDLLTKMGAQISFEQGKPEVSANNKPITVLT
LQLDDEYRLYSPLVKPDQNIQFWLEQFPQAWAETAGMGLAKQVPPQVIQLKASAAPVSVR
QYPLSKEAREGIRPHVQRLIQQGILVPVQSPWNTPLLPVRKPGTNDYRPVQDLREVNKRV
QDIHPTVPNPYNLLCALPPQRSWYTVLDLKDAFFCLRLHPTSQPLFAFEWRDPGAGRTGQ
LTWTRLPQGFKNSPTIFDEALHRDLANFRIQHPQVTLLQYVDDLLLAGATKQDCLEGTKA
LLLELSDLGYRASAKKAQICRREVTYLGYSLRGGQRWLTEARKRTVVQIPAPTTAKQVRE
FLGTAGFCRLWIPGFATLAAPLYPLTKEKGEFSWAPEHQKAFDAIKKALLSAPALALPDV
TKPFTLYVDERKGVARGVLTQTLGPWRRPVAYLSKKLDPVASGWPICLKAIAAVAILVKD
ADKLTLGQNITVIAPHALENIVRQPPDRWMTNARMTHYQSLLLTERVTFAPPAALNPATL
LPEETDEPVTHDCHQLLIEETGVRKDLTDIPLTGEMLTWFTDGSSYMVEGKRMAGAAVVD
GTRTIWASSLPEGTSAQKAELMALTQALRLAEGKSINIYTDSRYAFATAHVHGAIYKQRG
LLTSAGREIKNKEEILSLLEALHLPKRLAIIHCPGHQKAKDPISRGNQMADRVAKQAAQG
VNLLPMIETPKAPEPGRQYTLEDWQEIKKIDQFSETPEGTCYTSDGKEILPHKEGLEYVQ
QIHRLTHLGTKHLQQLVRTSPYHVLRLPGVADSVVKHCVPCQLVNANPSRIPPGKRLRGS
HPGAHWEVDFTEVKPAKYGNKYLLVFVDTFSGWVEAYPTKKETSTVVAKKILEEIFPRFG
IPKVIGSDNGPAFVAQVSQGLAKILGIDWKLHCAYRPQSSGQVERMNRTIKETLTKLTTE
TGINDWMALLPFVLFRVRNTPGQFGLTPYELLYGGPPPLAEIAFAHSADVLLSQPLFSRL
KALEWVRQRAWKQLREAYSGGDLQVPHRFQVGDSVYVRRHRAGNLETRWKGPYLVLLTTP
TAVKVEGIPTWIHASHVKPAPPPDSGWKAEKTENPLKLRLHRVVPYSVNNSSSZWZTPCG
QPELPZTLISHLVTYZLRYRYZYZQHSRGGSLGDLVAZIICLPSISNPWSQZPGHTPRCT
PCLRVLRLPRTPKZZRILWKSSGFLLQAMELHNFZZWELEMASLSARQSKLLFCZQSYQL
ZSIZLWPWEMERLATAGTKRCTKZANKLSFVRPRLLKNKFHZKRKTRKYSKVGKWYILGN
SVLWRLWEKERICSDYSPQNRNSDGTSGCYRTKZGFGRTRTSNPRTEAISZPLZLQYNLW
ISPHZAZHHYZNRGETFZPHPGSFSSSZLHDSRGYLFLLALLSFGPTLLZGNGZRREIQC
DKGTZRPMYMGIPKZAYPYZGFWKRHLHRDGSPIPPTPLZPHZSLZSNLZESISGTWLZQ
VVGMZYWINPLCFHLGFQPNZRLLRYGPNCPPGVLLSRKSSPZZIZLZIZSAKKRAHIPD
TSCNARIGSGCRRGNRNGCPNHRTATAGERTZZPTSNCNGRSPSPRKICQZPGGIPNLLI
ZSGSTEQKGVRSVISKRRRVMCSLKRGMLLLCGSFRSYQ
>pt01-Chr10r-17119458
GPVVTNPHNRTMSILTINLEDEYRLHQEKAAPDQDIATWLQQYPEAWAEMGGLGLAKHRP
ALFIELKPGTDPMRVRQPMPLEAKRGIAPHIRRLLDQGVLRPCHSPWNTPLLPVRKPNSG
EYRPVQDLREINKRVVDIHPTVPNPYTLLSTLNPKHQWYTVLDLKDAFFSLPLAPQSQKL
FAFEWNDPDRGISGQLTWTRLPQGFKNSPTLFDEALHEDLGEYRRKHPEIILLQYVDDLL

IAAETQEACIQGTKGLLQALGNLGYRASAKKAQICKTEVIYLGYLLKGGQRWLTDARKQN
VLQIPRPQSTQQVREFLGSAGFCZLWIPGFAELAKPLYQATRGQQPFNWTDEAELAFQQI
KTALLSAPALGLPDVTKPFHLYVDENKGVAKAVITQNLGPWRRPVAYLSKKLDPVAAGWP
LCLZMIAAMALMVRDADKLVMGQELRVVTPHAIEGVLKQPPNRWMSNARLTHYQGLLLNP
LKIIFLPPTTLNPASLLPNPDLDAPLHDCTEILAQVHGVREDLQDRPFPDADLVWFTDGS
SFMHQGQRYAGAAVTSETEVIWAEPLPPGTSAQKAELIALTQALTLGAGKKLTVYTDSRY
AFATAHIHGAIYRERGLLTAEGKEIKNKQEILALLTALWRPEKLAIVHCPGHQKLTTPTA
QGNFLADQTARNVAKAPSQLLALQLPDLGPRDLPYFPEYSEQDLQWIDKLPLKQIQNGWW
TDTNDQTILPEKLGQQVLEHIHRTTHLGARRMIDLIRRSKLKIRHIDETASSIVTSCKVC
QLNNATPKSQAAAGTRLRGTRPGIYWEVDFTEIKPGKYGYRYLLVFVDTFSGWTEAFPTK
RETAQVVAKKILEDILPRYGFPIQIGSDNGPAFVAKVSQDLASILGANWKLHCAYRPQSS
GQVERMNRTLKEALTKLTIETGANWVVLLPIALFRAHNTPYKLGLTPYEIMYGRPPPLVP
SLKDDLLKSETENVSEFLFSLQALQKIHQEIWPKLRELYETSPPPTPHPYQPGDWVLVKR
HRQETLEPRWKGPLQVLLTTPTALKVEGIASWIHYTHVKPVDPTSDLLGPITAAAAEAPD
TWTVDRAKNNPLKLTLHRQHNSLQTCS
>HERV-T
NTLLLDLQTLFPRVSAESNPPGLAKHHPPVVIELLATAIPVQAKQYPTSQQAREGINPHI
QRLLQAGILTPCQSAWNTPFLPVQKPGTNDYWPVQDLRDVNKWTVAVHPTIPNPYTLLSL
LPPEHTVYTVLDLKDAFFAIPLAPKIQPIFAFKWTDPRSGDTTQLTWTQLPQGFKNSPTL
FGEALQQDLTFRASHLNCTLLQYVDDILITTETMDGCLQHTRDLLYLLQELGYGVSAKKA
QLCLPRVSYLGYKINKGKRALTSARKEAILRIPTPATKRQVHEFLGALGYCRLWISGFAE
ITKPLYTATKGNGPLIWTDTEEQAFQNLKKALTAAQALALPNISKPFHLFVHESQGVAKR
VLTQTLGTWRGPVAYLSKKVDPVASGWPSCLAAIVATASLVQETDKLTLGQNLTLTVPHA
VETZLRSASGKWMSNARILQYQSLLLDQPCLTFSPTRCLNPATLLPDPDSNTPVHDCQEL
LEATETGRPDLQDVLLRKADATVFTDGSSFLEQGIRKAGAAVTTETDVLWVQALPASTSA
QKAELIALTQALRWVKDKRINIYTDSRYAFATVHVHGAIYZERGLLRSEGKIIKNKEEIL
ALLEAVWLPQQVAVIHCKGHQKENTAVARGNPKAVSAAREVALSSAPSINLLPAVSFPQP
DLPDNPAYSTEEEKLAANLRANKNQKGWWILPDSRIFVPPALGETLVSHLHSTTHLGTKL
AQLLRSCFKIPHLQSLTDQAVLWCTACAQVNAKQGPKPSPGHRLRRNSPGEKWEIDFTEV
KPHRAVYKYLLVSVDTFSGWTEAFATKNETANTVVKFLLNEIIPWYGLPAAIGSDNGPAF
TSSIAQSVSKALNIQWKLHCAYQPQSSGQVECMNHTLKNTLTKLILDTSENWVKLLPLAL
LRVRCTPYQAGFSPFEIIYGRAPPILPKLKDAHFAEISQANLLQYLQSLQQVQEIILPLV
ZEAHPSPVPDQMGPCHSFQLSDLVFVKKFQREGLTPAWKGPHTVILTTPTALKVDGIPAW
IHHSHIKKANKAQTETWVPKPGSGPLKLHLSRVKLLN
>ERV-3
APMQPCTQEEEKLLFRPIDKIPGVWAEDKPPGLAVNQALVVELKPGATPVQVCQYPLSQE
DIWGIYKHLKWLCDHGIIIQCQSPWNTPLLLVQKPLPGPGSDEYIPVQGLHAVNQATVTI
HPVVPNLYTLMGLILASATWFTVLDLKDAFFCLYLAPVSQPIFAFZWDNSVTGTGGQLAW
TSLPQGFKNSPTIFGEALASDLKAYTPPNDNCALLQYIDNLLLAAPTQEDCYWGTQDLLH
LLWKAGYRVSKKKAQICHEKVKYLGFIVSQGERWLGHGZKQAICALPTPTTWRQIGEFLG
AAGFCHIWIPNFSLIARPLYEATREGEKEPLLZKADQKKVFKQIKEALTQAPALGLPDTT
KPFFLYVHELKGMAIGVLTQIIGSWHRPVVYLSKQLTVVLGWPPCCKALATTTLLTQEAD
KLTLGQQLTIQVPHAVITLMDQREQHWLSNPTMTZYQGLLCENPRITLETVNTLNPATLL
PVKPGITFRDCGNVDEVFSSRGDLTDQPFKDPDVEYFIDGSSFVLEGVCWAGZAVVTLDS
VVEAZPQPAGTSAQKAELAALTRVLQLAEDKKISVYTDSKYAFATLHVHGAIYKERGLLT
ATGKEIKWKEZILQLLDAVWIPEKVAAMHCRGHQRAGTSEAKGNRKADREAKWAAMVTPH
FKKEALAMPLLPEPPLQEVSSYSPNEKAVLPKKLENILKEDGGNFDGRSAIPKMVAPKFV
KZLHHELVGKTALETLLGRHFYVPRLTAITQAVCEQYLTCAQNNPZQWPTRPPGIQEIGT
MPCENLLMDTELPQAGSYRYMLVLVYTFSGWAKAFPTRSKNSZEVTKILLRNIIPRFGLS
VTLGSDNRLAFLAEIIQELTRVLKIKWKLH
>HERV-E
SKLRATISFTEHGSLLLKLPGTGVIMTLMLPREEEWRLFLTEPGQEIRPALAKRWPRVWA
EANPPGLAVNQAPVLIEVKPGVQPVRQKQYPVLREALEGIQVHLKCLRTFRIIVPCQSPW
NTPLLPVPKPGTKDYRPVQDLRLVNQATVTLHPTVPNLYTLLGLLPAEDSWFTCLDLKDA
FFSIRLAPERQKLFAFQWEDPESGVTTQYTWTQLPQRFKNSPTIFGEALARDLQKFPTRD
LGCVLLQYVDDLLLGHPTAVGWPREQMLYSGTWRTVGIRCPRKKAQICRQQVCYLGFTIQ
QGEHSLGSERKQVICNLPEPKTRRQVREFLGAVGFCRLWIPNFAVLAKPLYEVTKAGDQE
PFEWGSQQQQAFHELKERLMSVPALGLPDLTKPFTLYVSESEKMAVGVLTQTVGPWPRPV
TYLSKQLDGVSKGWPPCLRALAATALLVQEADKLILGQNLNIKDPHAVVTLMNTRGHHWL
TNARLTKYQSLLCENPHITIEVCNTLNPATLLPVLEIPVEHDCVEVLDSVYSGHQTGNYT
WMGAALSTHKKRDVQGMRWLWTLLLKPDRFPRALQLRKLNSLLFGPNSVKVRLTFTLTLD

MSFPFKCMEHYVKKRAYTLGEKTNINKKSCNYKQYGNPTRWLLYIAEDTSELPPWWVWGI
PALTRLKKQHLPFRASVTAPLLPQAPDLVPTYSKEEKDFLQAEGGQVMEEGWIWLPDGRV
AVPQLLGAAVVLAVHKTTHLGQESLEKLLGWYFYISHLSALAKTVTQRCVTCRQHNARQG
PAVPPGIQAYGAAPFEDLQVDFTEMPKCGGNKYLLVLVCTYSGQVEAYPTRTEKAHEVTR
VLLRDLIPRFGLPLRIGSDNGLVFVADLVQKTAKVLGITWKLHAAYQPQSSGKVERMNRT
IKNSLGKVCQETGLKWIQALPMVLFKIRCTPSKRTGYSPYEILYHRPPPILRGLPGTPRE
LGEIELQRLQASGKITQTISAWVNERCPVNLFSPVHPFSPGDLVWIKDNVACLCPRWKGP
QTVILSTPTAVKVEGIPTIHHSRVKPAVPETWEARPSPENPCRVTPKKTTSPAPVTPGSL
VHARPKHAEAHHGIHFSILDLYSKGFN
>gg01-ChrU-126703652
PLRAEDREGMQPVIDRFINHGLLVECESKYSITILPVRKPDGSYWVVZNLRAINKIVEDL
YPLVANPYTLLTKLSTELMYFTVLDLKDAFCLPLAAESQLLFAFEWENPSTGRKSQLTWT
VLPQGFKNSPTIFGNQLAKDLEQWERPSNEGVLLQYVDNLLIATKTKEDCLKWTISLLNF
LGLNGYRVSPQZAQVAQQQVTYLGZEIAAGMRTLGTAQKEGICQTPEPRTSRELRTFLGM
MGWCHLWIPNYELLVKPLYVLLKSCPKDLIWDGETRRAFQQLKQELMKAPALGLPDVTKP
FLLFSYEKQGIALGVLAQNVEPYQRAVAYFSKQLDEVSKGWPGCLRAVAALVVNIQEAWK
FTMGQKITMLVSHVVSTVLEVKGFLKYZAVLVEQDDVEIMITNIINPASFLSGVTGEPIS
HDCLETIEAVYSCCIDLKEDPLEDGEDTWYTDRSSFVZQGICKAGYAVFTLMRZLEAKAL
PPSRGHRRLRYSLNKGLEXXXXXXXXXXXXXXXXXXXXXXXXXXXXXXXXXXXXXXXAEE
MDNALADREAKQAAEGKVVEGSLIPDGKIQVDGEPRYSKEDWNLISDLEGQVKEEGWAIT
PQGKLIIPTAILWAVVMAEHRKTHWGTEALYKYLVZQVAAZNLSTTTRQVTQQCEVCLQN
NPKTGHRVQLGQIGKGNYPGQQWQIHFSELPRKGGYRYMLVLTDTFSGWPETYPQRTNKA
REVTKILLQEVIPRFGVLGVISSDRCLHFVAKIVQQVSTLLGIDWQLHTPYRPQLSGQVE
KMNHZLKLQTVKIGQEVGIPWPQALPLALLRIRTKPZTKEKLSPYEILYGZPYAVQKGIS
MPVGDQVLSEYVISLEATQKLKKTIFGTRAQGLDGLVHDILPGNYVYVKSLSDSPLZPRW
EGPYQVLLTTHTAAKVEGLIPWIHHTRLKKIMRPQWTAEERPLRVVIKKNV
>HERV-ADP
NPLVWLREGNRGGLTITPLQIELKQAEEVVCRKQYPISLEGRKCLQPVIEELIKDGLLEP
CMSPYNTPILPVKSPGLYRLVQDLRTINQIIQTRHPVMPNPYTFLRKIPYEHKWFRVVDL
KDVFWACPLDFRSRDLFAFEWENPITGRKQZYCWTVLPQGFKFVQILENVLEESQTSRGT
QLLQYVDYLLISGEKRARVSETTTNLLNFLGERGLZVSKNKMQFVEKEVKYLGHLINEGK
WRINPERLLEIMGLPLPKTKRELZNFFGLTHYYRLWMSYAQKTKILYLKLLEEEPNPLZW
SPEEIQAVKELKQALITAPVLAFPPLEKPFHLFVTVDQGVALGLLTQTLGGKRQPVAFVS
KLLDSVSCRWPKCMKAVAATALLVEESQKLTFGGALIVSTPHQVRNILNZKARTWLMDSQ
ILKYEAILVEZDDLVITDTCLNLASFLZKGEENKETSDHNCLNIIEYQTKVRPDFREAPL
HVVLFVDGSSZVIDGRRHSRYTVIDENKHSLCEKGRLPNGWLAQTCELHALNQALNLLED
QEGTIYTDSKYAHGVLHTFGKIWTEQGLINSRZKELVHGELVKQVLESLLLPAEVAIVHV
NDHQIGNTVEAIGNRLADEAAKQASLEEEIRLFSLIPDISKVVLRPQFTKEEKEELDKIG
VTQTEDGKWVLPAGREMIKPLMRELMSILHKGSHWGPQALCDAILRNYVCIGIZTLTKQV
CGSCVICQSLNKMLARKQAMGGRPPGLRLFQSIQVDFREMPKIVRLKYLLVIVDHLSGWM
EGFPLPTATTRNVKIILEZIVPRFGLAENIDSDNGSHFTSRVLRGIMEGLQIRWDYRTPW
HPPSSGKVERMNQTFKKHITKLILETKMPCTTCLQIALLSIRTAPRKDGIAPYKLLYGLP
YLGRATDLPTMETKDQFLRNYLLAISSTLSSLRLKGLLIQVPLEFMVTFQLGDLVLVKTW
KEDKLHPSRDGPYQVLLTTETAMRTAEWGWTHYTGQMTGKRDPRRERERPVKSARVTQEP
LKLTQKNLKRKYGLASSGS
>gg01-chrU-49656081
PVVWVKEGNRGGLRITPLKIKLQEKYEIIRQRQYPIPFEGRMGLKPVIQGLLKDGLLEPC
MSPFNTPILPVRKPDGSYRLVQDLRKINEIVQKRHPAVPNPYTLMSKIPNENRWFSVIDL
KDAFWSIPLDHESRDIFAFEWEDPESGRKQQYRWTVLPQGFTESPFVLLLQYVDDLLLSG
PEKVEVKRATNELLNFLGKQGLRVSKNKLQYVEREVGYLGHLVSEGKRRITPERIQGIIE
LPLPRTQRELRKFLGVVGCCRLWIESYAQKTKRLYLKLLDGEPNILEWTAEEKQLIEELK
QSLITVPVLALPALNKPFQLFVTVDQGAALGVLTQEWGDKRQPVAYLSKLLDPVSQGWPQ
CIQAVAATALLAEESRKITFGRRLVVTAPHQVKTILTQKAGRCLTTCLNPATFLWKGEEK
EENIEHNCLDLIECQTKVRPGLQDTPLGEGKLLFIDGFSRVVHGKRYNGYAVVEGAEGEI
REMGQLPNNWSAQTCELYALNRVLHLLKGEEGTIFTDSRYAYGVVHTFGKIWEERGLINS
KGKELVHEALIRQILENLLLPTEIAVVHVKGHQKGNSITAQGNRIADKTAREAALQSZII
MNYLVPEVPPPPPKQIFSEKEKDTFRDLGAIESEGKWILPDGSELLNKAVTREILTILHQ
GSHWGVQAMCDAVLRKYVSAGIYTLAKQVCRECAVCQRVNNKVIRSRPTGGREPGIRPFQ
SIQVDFTELPKVGRVKYLLVLVDHLTGWVEAFPSVFATANTVTKVILEQIIPRYGVVEHI
DSHQGSHFTSQVLWKLMHTLEIKWEFHTPWHPSSSGKVERMNQTIKRHLTKLVLETRLPW
TKCLPLALLZIRTAPRKDVGVSPYEMLFGLPYLGKKNEIPQFETKDAFLKLYTRVILLLI

SSQNPRTISINPPLEFPVHPFQAGDWVLTQMWKETKLQPDWEGPFQVLLTTETAVRTAEK
GWTHYTRVKASTSPDAWEAVGTEEPLKIRIRKRKL
>HERV-I
PQVSPKGFLTSLNLLTTADEKYIHPDVWSREENZGKLRILPIHIKLNTPHWEVVRRKQFP
IPLEGMLGLKPIIESLINDGLLEPCMSPYNTPILPVKKSDGSYRLVKDLRAINQTVQTTN
PVVPNPYTILSKIPYNHQWFTVIDLKDAFWACPWLESRDTFAFEWEDPQLGZKQWYQWTV
LPQGFMDSPNLFGQILEQVLDKVSVPKQLCLLQYVDDILISGEDIEKEAGFSTHIFDHLQ
FEGLRVSKGKLQCMEPKVKYLGRLISAGKRRIGPEWVEGIVSLPLPQTKQELRKFLGLVG
YCRLRINSHALNSKLLYQKLAQGKPEHLLWTSKEVDQVKELKGILITALALALPSLENTL
HLFVSVKNGVALGVLIQEHRGCWQPMAFLSKILDLVTCGWPQCIQSIAATAVLVEESRKL
TFGWRLTVSTPHQVRAILNKKAGRZLTDSRILKYGAILLKKDEEHLCLDLIDYQTKLRPD
LGKIPFKTGRHLFIDGSSQLIEGKRHNGYPVIDGESLCIRTESRKLPNNWSAQTCELFAL
SQALKHLQNQEGTIYTDSKYAFGVAHTFGKSWTECGLTNSKGHDLVHKELIIQVLDNLQL
PEEIAIVRVPGHQKSLSFESRGNNLTNQIAKQAAVSSETPMFHLTPCLPSPTAISFFSSI
EKEEZRZEPKEKTRMKTAVTRPKGNVIQARYVEDLVSTYLRTHWGSQAMCDAVLRVYGCI
RIYTLAKQVTDSCLICKKTSKQILRKPPLGKRFRAKTISKCSINYTEMPPIGHLKYLLVI
IDHFTYWVETIPHSNATTSNVVKALIENIVPRFGLIQSTDSDNGTHFTAYVIKKLAQVLD
IKWKNHIPWHLSSSRRVERMNQTLKSHLTKLVLETZLPWTKCLPIALLRIRTAPQRDTGL
SPYEMLYGLPYLYSTANIPTFKIKDQFLKNYILGPYSTFSSLKTKGLLAQAPPLEFPAHZ
RQPGDHVLIKGWKEGKLKPAWEGPYLVLLNTKTAVRTANEDGLITPASKRRCHLQDHKPS
LQGSPQPN
>HERV-Hconsensus
PQVWDISTPSLATDHMPITIPLKPNHPYPAQCQYPIPQHALKGLKPVITRLLQHGLLKPI
NSPYNSPILPVQKPDKSYRLVQDLRLINQIVLPIHPVVPNPYTLLSSIPSSTTHYSVLDL
KHAFFTIPLHPSSQPLFAFTWTDPDTHQAQQITWAVLPQGFTDSPHYFSQALSHDLLSFH
PSASHLIQYIDDLLLCSPSFESSQQDTLLLLQCLFSKGYRVSPSKAQISSSSVTYLGIIL
HKNTRALPADRVRLISQTPTPSTKQQLLSFLGMVGYFRLWIPGFAILTKPLYKLTKGNLA
DPIDPKSFPHSSFHSLKTALETAPTLALPDSSQPFSLHTAEVQGCAVRILTQEPGPHPVA
FLSKQLDLTVLAWPSCLRAAAAAALILLEALKITNYAQLTLYSSHNFQNLFSSSHLTHIL
SAPRLLQLYSLFVESPTITIVPGPDFNPASHIIPDTTPDPHDCISLIHLTFTPFPHISFF
PVSHPDHTWFIDGSSTRPNRHSPAKAGYAIVSSTSIIEATALPPSTTSQQAELVALTQAL
TLAKGLRINIYTDSKYAFHILHHHAVIWAERGFLTTQGSSIINASLIKTLLKAALLPKEA
GVIHCKGHQKASDPIALGNAYADKVARQAASSPTSVPHGQFFSFTSVTPTYSPTETSTYQ
SLPTQGKWFLDQGKYLLPASQAHSILSSFHNLFHVGYKPLARLLEPLISFPSWKSILKEI
TSQCSICYSTTPQGLFRPPPFPTHQARGFAPAQDWQIDFTHMPRVRKLKYLLVWVDTFTG
WVEAFPTGSEKATAVISSLLSDIIPRFGLPTSIQSDNGPAFTSQITQAVSQALGIQWNLH
IPYHPQSSGKVERTNGLLKTHLTKLSLQLKKDWTVLLPLALLRIRACPRDATGYSPFELL
YGCTFLLGPNLIPDTSPLGDYLPVLQQARQEIRQAANLLLPTPDPQPYEDTLAGRSVLVK
NLTPQTLQPRWTGPYLVIYSTPTAVRLQDPPHWVHHSRIKLCPSDSQPNPSSSSWKSQVL
SPTSLKLTRISEEQ
>HERVH-RGH2
SIPPSTTHYSVLDLKHAFFTIPLHPSTQPLFVFTWTDPDTHQSQQLTWAVMLQGFRGSPY
YFSQALSHDLFLHPSLLLIQYIGDVLLVAPPELLNKTHFLLQHLFSKGYPSKAQMSSPSV
TYLGIILHKNTRALPADSVZLISQTPTPSTKQQLFSILGMVGYFRVRIPGFAILTKPLYK
LTKGNLVDPIDPKSFPHSSFHSLKTALETVSTLALPDIPTLFIHTAEVQGCAVRILTQGP
GSHPVAFLSKQLDLTVLGWPSCLHAASAATLILLEALKITNYAQLILYSSHNFQNLFSSS
HLTHILSAPRLLQIYTPFILPITIIPGLDFNPASHIILDTIPDPHDCISLIHLTFTPFPH
ISFCPVSHPDHTWFIDGSSTRPNRHSPAKAGYAELVALIQALTLAKGLRVNIYTDSKYAF
HILHHHAVIWAERGFLTTQVSSIINASKKTLLKAALLPKEAGVIHCKGHQKTSDPIALGN
AYADKVARQAASSPTFVPHGQFFSFTSVTPTYSTAETSTYQALPPQGKWFLDQGKYLLPA
SQAHSILSSFHNLFHVGYKPLACLLGPLISFPSWKSILKEITSQCSICYSATPQGLFRPP
PFPTHKARGFAPAQDWQIDFTHMPRVRKLKYLLVWVDTFTGWVEAFPIESEKATAVISSL
LSDIIPWFGLPFSIQSDNGPAFTSKSQAVSQALGIQWNLHIPZHPQSSGKVKPTNGLLKT
HLTKLSLQLKKDWTVLLPLALLRIRTCPRDAQGTPFELLYGRTFLLGPNLIPDTSPLGDY
LPVLQQARQESHQAANLLLPTPDPQPYEDTLAGRSVLVKNLTPQTLQPRWTGPYLVIYST
LTAVRLQDPPHWVHHSRIKLCPLDSQPNPSSSSWKSQLLSPTS
>HERVH-RTVLH2
ASLTIPRLZHTHCHLFPSSKPPSHPPLVSPLLNPQVZDTSIPSLVTDHAPLTISLKPNHP
YPRQCQYPIPQHALKRLKPVITRLLQHGLLKPINSPYHSPILPVLKPDKAYRLVQNLHLI
NQIVLPIHPMVPNPYTLLSSIPPSTTHYSVLDLKHAFFTIPLHPSSQRLFAFTRLTLTPI
RLQITWAVLPQGFTDRPHYFSQAQVSSSSVTYLSIILIKTHVLSLLIMSNZSPKPQFFTK

QQLLSFLGMVSAVRILTQEPGPHPVAFLSKZLNLTVLAZPSCLRAAAATALILLETLKIT
NYVQLTLYISHNFQNLFSSSHLMHYFLLPGSFSCTHSLLSPTITIVPGPDFNPASHIIPD
TTPDPHDCISLIHLTFTPFPHISFFPIPHPITLDLLMASTRPNCHTPAKADYAIVQATSP
PLRTLISFPSWKSIPKEITSQCSICYSTNPQGLFRPPPFPTHQAZGFAPTQDWQISFTZH
APSQITXKYLLVZVDTFTGZVDAFPTGSEKATVVISSLLSDIIPRFGLPTSIQSHSRPAF
IGEISQAFFQALSIQZNLYIPYSPQSSGKVEQTNSLLKTHLTSSATNLKRTRQYFYHFPF
SEFRPVLRMLQGTAHLSSYRYSFLLGPSLIPDTRPIWTVLQKTCHPYYLLSS
>HERV-Fc1
TKFPSRPQFPISVEHRQGLKPIITRLLQQHILIPVNSRCNTPILPIRKASGAYRLVQDLR
IINEAVVPIFPVVPNPYTLLSRIPPTTTHFTVLDLKDDFFTIPLHPDCYFLFAFTWEDPD
THVSSQFAWTVLPQGFRDSPHLFGQALAKDLSTCTLADSTLLLYVDDLLLCSPSLSVSQQ
DTATILNFLGKQGYRVTPHKVQLCTPTVTYLGISLTATTKSLTTDRVSLIKDLQLPQDAD
KILSFVGLVGFFRHWIPNFGVLAKPLYQAAKETPTSPLSDPALVARHFHRLQQCLLTAPV
VSLPNPLRPFHLYTDELQGVATGLLGQPVGPTYQVVAYLSRQLDPSTRGWQPCLRALAAA
AELTKEALKLTLSHPLTVYSPHRLTDVLSHKCLAHLAPSRIQLFHVLFVENPDITLTASP
PLNPATLLPIEASEPPPVLSHSCPELLTSNPNSRLGLFDRPLSNPDSTLFVDGSSVLTPC
GRRQAAYAVVTHDKTVEAAALPLGTTSQKAELLALTRALLLSQGQRVNIYTDSKYAYSIA
HTHSVLWQERGFLTMKGTSIVNGPLIHKPLNALQAPREVAIIHCKSHQHSKDPVAQGNNL
ADSTAKSLALTSAPAPAPAMFLSGSRTPAYSPQETFHLISNLKGMTDQDGZIWVDNRIAL
PESQAQAIITDVHKTLLIGPKLLHQFLEPIFLCPGLQSLIHQVHQTCAVCSTVNTQGGLR
RPGPHHQLRGHQPGEDWQLDFTHMPRHKHYRYLLTLVDTFTGWIEAFPTARETGEVAVSV
LLEHIIPRFGLPRSLQSDNGPAFVSKITQQVSESLRVTWKLHIPYRPQSSGKVERANSLL
KEHLTKLTLETKLSWVTLLPLALTRLRAAPRGPTGLSPFELLYGRPFLLPGLPPTVSPPP
LASYLPYLTLLRDLLRKHADACLPEPTPSSPDAPVVLSPGDSVLLKELQSKTLTPRWSGP
YTVILTTPRATKLLGLPSWYHLSQLKKAPTQHDWSSKLTPTRLRITHPDLPHYASYS
>HERV-Fc2
PQVWDTNTPVVATHHKPVLIKLKDPLKFPAPPQFPISLEHRRGLKPIIIRLLQQHILITT
NSPCNTPILPVRKASGTYCLVQDLRLINEAVIPTVPVVPNPYTLLSRIPPSRSHFTVLDL
KDAFFSIPLDPACYFLFAFTWEDPDTGVSKQLTWTVLLQGFRDSPHFFGQALAQDLARCP
LEASTVIQYVDDLLLCSPSETDCLKDTCTLLNFLGNZGYRVTPSKAQLCTSKVTYLGIRL
TPNSKGLTSDRISLLQNLQPPQDAEDILSFLGLVGFFRHWVPNFGVLARPLYQATKQTPL
GPLSEPKLVANLFNKLKNCLITAPVLSLPNPLRPFHLFTDEREKVATGLLAQLVGRTHQP
VAYLSKQLEPTVQGWQPCLQALAAAAKLTKEALKLTLGHPLTVFSSHRLQDLLSHKCLSH
LTPSRLQLFHLLFIENPHITLTTSPTLNPATLLPCPEQDPASLHSCSZILSTLPPAHLSL
QDNPILDPHQTLSVDGSSISTPQGQRRAAYAVVTSSQVVEAKPLPTGITSQKAELIALTR
ALILSKNKKVNIYTDSKYAYLIAHTYSILWQERGFLTTKGTPIVNGPLIEKLIQVLKAPT
QVAIIHCKSHQNSKDPISLGNNFANTTARATALLAPSPTPVCFLSPAYTPDYSPEELVHL
MGRSGVKTNSNERFHSHTPNKSNQGWIFVDDRVVLPCSQKKLILTDMHRSLHIGPKALYN
FLEPIIYHPSLYSLLKQIHQECHVCTVANPQGKLKLPGPCHQLREHQPGEDWQVDFIHMP
RTKSSVIAPGPSDFLLRLLYRKALPDSPSQDGSLCFQEIGSLGPGAVDHACNPRTLRGRG
GQITZDREFKTQPEQHEETPSLLKIQIRSSPSPSPSPHGLPLNGAQAGVQWPDLGSLQPP
PPSCLPWPPKVPRLQPLRLPRRG
>HERV-W
NIGEGTPVCCPLLEEGINPEVWATEGQGRAKNARPVQVKLKDSTSFPYQRQYPLRPKAQQ
GLQKVKDLKAQGLVKPCSNPCSTPILGVQKPNRQWRLVQDLRIINEAVPLZPAVPSPYTL
LSQIPEEAEWFTVLDLQDAFFCIPVHPDSQFLFAFEDTSPTSQLTWTILPQGFRDSPHLF
GQALPRLEPILIPGHLSFGVDDLLLAAHSETLCHQATQAFNFLATCGYMVSKPAQLCSQQ
YLGLKLSKGTRALSEEHIQPILAYPHPKTLKQLRGFLGVIGFCRKWIPRYGEIARSLNTL
IKETQKANTHLVRWTTEVEVAFQALTQAPVLSLPTGQFSSYVTEKTGIALGVLTQRGMSL
QPVAYLTKEIDVAKGZPHCLRVVVAVAVLVSEAVKIIQGRDLTVWTSHDNGILTAKGDLW
LSDNCLLKCQALLLEGPVLRLCTCATLNPATLPDNEEKIKHCQQVISQTYATRGDLLEVP
LTDPDLNLYTDGSSFVEKGLRKVGYAVVSDNGILESNPLTPGTSAQLAELIALTWALELG
EEKRANIYTDSYAYLVLHAHAAIWKEREFLTSERTPIHQEAIRKLLLAVQKPKEVAVLHC
RGHQKGKEREIENCQADIEAKRAARQDPPLEMLIKQPLVZGNPLRESPSTQQEKQNGEPH
EDSFLPSGRLATEEGKILLPATIQWKLLKTLHQTFHLGIDSTHQMAKSLFTGPGLFKTIK
QIVRACEVCQRNNPLPYRQAPSGEQRTGHYPGEDWQDFTHKPKPQGFQYLLVWDTFTGWA
EAFPCRTEKAQEVIKALVHEIIPRFGLPRGLQSDNSPAFQATVTQGVSQALGRYHLHCAZ
RPQSSGKVEKMNETLKGHLKKQTQETHLTWPALLPIALKRICNFQKAGLSPYEMLYGRPF
ITNDLVLPKTANLVADITSLAKYQQVLTLQGTYPZEEGKELFHPCDMVLVKSLPSNSPSL
DTSWEGPYPVILSTPTAVKVAGVESWIHTLESNPGYCQRNLKIRRQR
>HERV-FRD

```
LTHGLISCNSPYNTPILTVKKTNGEYRLVQNLZVINEAVVRIHPMPQPLCNSRRFPDAQW
FSVLDFKNFFCIPLDPSSQFIFVLELENEKGRSZQLTWTVLPQGFRDSPHLFGQALARYL
QDLSLYMGGHLLQYMDDLLICSPTRELGIQHLNFLADRRYKVSKAKTQLLRQAIQYLGII
MSLKEHKLSAEZIQAILRITPTTQKQFZAFLGITRYCRLWILECGGIIKSLYQALKEGTN
RDTIWEKNQEQAFRZLKTALSQAPALELPILTKPFQFFITEKAGIALGVLTETFGPVKHA
VGYFSKNLDPAAQWWPHCLKVVGTAALILEEDFNITMGQSIWLLMSHQIDPLLNLKGPZW
LTDNTLIKYQVLLLENPQVTVEQCSTINPALLLPLPGDDNSTYSCCEILNKIYASWEDLK
DQPIDNPDKIWFSDGSSFVRDGTRYAGYATESHHQVVETKALSPGTSVQLAEFITLTRAL
KLGERKRITIYTDSKYAFLVLHAHMAIWKERRYLTAQDAPIKYGSQVLELLEAAHLPQEV
AVEHCEGHHRGFYZTTWGNRLADKNAEEAAMSNDTFMGDLFLSLPSELPFLQYTKEEIDW
SSQHGYKEETSGWYRLEEFSIYLPMESLQGKAFHWEVDGEIGGEMKKLAGRWRKVWGILV
DAELVVGVCLVSLLVRVYSESTLQTVLGRAPISIPFFFSAIMQIGAIPIKVILEKMGPKM
EPPNGVIKSFYNFFHLAKDSLGQICKZVFSGKGLNKTIQQVCQAYTLCTINNSQRGKPPP
LISPIQRRSTYPGEDWQMDFTHLPAGFRZKYLLVCVDTFTKWVEACSTKTEKAQEVAKFL
LKECISWFELPRSLQSDNGPSFISZVTQQVSRAVSKHKCTFTLPGDQPSGKVERTKETLE
HILNKLCQKTAQPWVDLLLLAPLWIHIAPQTPLQLIPFEALYGKPFLYSDLMLDEKTAKI
TYCVSSLAGFQQALQEFWLQRKPKSEGEKYQPLYPPGSLVLIKAWRDGTLNSQLTPVRRG
LFTLRAIKVSEIVSWIHHLSEAMERLQNTRSGSNALSSRTHLRGT
>MER4like
PILLLQDRLFVSSLETNINPEVWATQGEIGQARTSTLIWIHLKDLTSFPNQKQYPLKPEV
RKGLEAIIDNLRMQGLLKLCNSPCNTLTLGVQKPNKEEWRLVQDLHTINKAVVSIHLVVP
NPYILGTKWFTVLDLKDAFFLHTIIPSSQYLFALEDPSNRTTQLTLMVLPQGFZDSPHLF
GKVLSKGLSEFLYPQVKVLQYVDDIPLGFPSEKISQEGRKALLNYLANRRYKVSKSKSZL
CQTSVKYZGLVLSDGTRMLGEERIKPISFLPLPQTLNQLRRLLGITGFCRLHTPGYGVTV
ZHFYHLIKETKAAKSHSLICKEAAKRTFLVNPEYVRQVSVNLESLFCQGZKHAPMTQPQE
ILMTCSQGGQSSLVLYILGRYETPINICKMNIVQSGEVGQLEAKAEKLKVRRGLSGHRZI
RDKRMIPLSFZLASPNEAIRYAFILASSGLILNRMGGSLPZVVSSLTFTFSLVILGPQDZ
FSFYRPVKTSLAKGTSLSLPIRELFNLYVLERKRMALGVLTZAQGPAQQPIGYPSKEFNL
VAKRWSACRQAVALVALLVPEATKLTKGNNIAIHTPHNVAELLSSGESLDYGQLLPQIIS
SAIRGICSPVKKLSLPKPSHLLPSESWGANITANTSANLHSQRGPERNPLRETTLDSLWR
SSFVEQRIQKAGYAAIILNNIIESTLLSWAQSQIAELIALKRRLELSKGKAVNISTDTKY
AFLVPSAHATIZKERDFLTPMGLPLNTIRILTRLLSLIFLPWEVAIIHCKGQQKGTYKIA
EGNKLAEQAAKLSVKGPEISDPLEPPLIERLHKRNKTSVFLCGDGMGHFLNIPFSPQDGC
NERMANFIYQLPDNEKHKTLHQAFPLGQDKTYLLAQRLFTGKNLLKMAKQVANALKTALK
IIPSIDGFFPLEPKDCEATHKDWQMDFTHIPMIRGLQFLLVWVDTFTNWVEAFPCCTENA
SEVRKVLITEKIPHFGLPWYLQTDYGSCFKAVVNQAGAGGARSSKTYKTVFSAWRPQFSE
KVEKTSDIIRKLSQETHLPQIILHVALLCVRHTPLKLGLNLLSLFEMVYRZPFLTNDCLL
DQZTSDLIKHVTSLAICQQELKQLLEAQSHEPRPPLFDPGDLVLIKALLSLSSTLGLKLK
GCYTVLLPTPTAVKVTGIDSCINYTZVKAZGTDRITSFDPEEHPSSSVMKSDPKLKTTKD
>WDSV
DPIDCPYEKSGTKTTQDVITTKNAEIMVTVNHTKIPMLVDTGACLTAIGGAATVVPDLKL
TNTEIIAVGISAEPVPHVLAKPTKIQIENTNIDISPWYNPDQTFHILGRDTLSKMRAIVS
FEKNGEMTVLLPPTYHKQLSCQTKNTLNIDEYLLQFPDQLWASLPTDIGRMLVPPITIKI
KDNASLPSIRQYPLPKDKTEGLRPLISSLENQGILIKCHSPCNTPIFPIKKAGRDEYRMI
HDLRAINNIVAPLTAVVASPTTVLSNLAPSLHWFTVIDLSNAFFSVPIHKDSQYLFAFTF
EGHQYTWTVLPQGFIHSPTLFSQALYQSLHKIKFKISSEICIYMDDVLIASKDRDTNLKD
TAVMLQHLASEGHKVSKKKLQLCQQEVVYLGQLLTPEGRKILPDRKVTVSQFQQPTTIRQ
IRAFLGLVGYCRHWIPEFSIHSKFLEKQLKKDTAEPFQLDDQQVEAFNKLKHAITTAPVL
VVPDPAKPFQLYTSHSEHASIAVLTQKHAGRTRPIAFLSSKFDAIESGLPPCLKACASIH
RSLTQADSFILGAPLIIYTTHAICTLLQRDRSQLVTASRFSKWEADLLRPELTFVACSAV
SPAHLYMQSCENNIPPHDCVLLTHTISRPRPDLSDLPIPDPDMTLFSDGSYTTGRGGAAV
VMHRPVTDDFIIIHQQPGGASAQTAELLALAAACHLATDKTVNIYTDSRYAYGVVHDFGH
LWMHRGFVTSAGTPIKNHKEIEYLLKQIMKPKQVSVIKIEAHTKGVSMEVRGNAAADEAA
KNAVFLVQRVLKKGDALASTDLVMEYSETDEKFTAGAELHDGVFMRGDLIVPPLEMLHAI
LLAIHGVSHTHKGGIMSYFSKFWTHPKASQTIDLILGHCQICLKHNPKYKSRLQGHRPLP
SRPFAHLQIDFVQMCVKKPMYALVIIDVFSKWPEIIPCNKEDAKTVCDILMKDIIPRWGL
PDQIDSDQGTHFTAKISQELTHSIGVAWKLHCPGHPRSSGIVERTNRTLKSKIIKAQEQL
QLSKWTEVLPYVLLEMRATPKKHGLSPHEIVMGRPMKTTYLSDMSPLWATDTLVTYMNKL
TRQLSAYHQQVVDQWPSTSLPPGPEPGSWCMLRNPKKSSNWEGPFLILLSTPTAVKVEGR
PTWIHLDHCKLLRSSLSSSLGGPVNQLLS
>hg15-chr3-152465283
```

```
PTDIGRIKSVQPMKVQIDHPKPLPKLPQYPLKSEAIQELZPIVEDLIKQGLILPCRSPCN
TPILAVKKPRMRLKICSRFTGNNKIVIPRFPVVPNSNILLSNVPTFQVFTIIDLCSAFFS
IPVYKESQYZFAFTWZNQQYTWPVMPQGFQSPFYFSQALHQDLMTLQLPQNSTLIQYIDE
YMLPTKECSVMDSVYLLQQLSYKGHKASVEKLKFSRQKVHHLGCDLAAEGISLSTKRITA
IQSFPZPATKRQLRSFLGLAGCCRFZVPIFSLIASPLYELTNNAIPEPLPWEDSHEQAVG
QIKLTLQQPSSLGLPNYTKPFTLFVHECNNQALGVLAQEHGGEHRPIAYYSLQLYPVTKA
YPNZLKAAAAVAKLVETSSDLVLGNELNLQITRCVKSIKFQPNPHFSVSRLTSYELLLLS
PSNLHLKTVIYLTLILLSLPNDGEDHNCVSVMIDLALCPHRNRTLNCNNPCGDYZIMGVG
LFHAVLMIVNSLMRSDGFIKGNSPAHALVCHHVRRYFATHSLLLPQPCGTVSZLKTSFLH
KWPSLRYVFISSENRIILVVSEIVALHVDFZDTPLDNPELIFFIDGSYVRNSEGKFQARY
TVTTQNELIEKGTLPQFKLAQPAELFALTZVCHIAKDKSVNIYTDSRCAFGILHDFGMIZ
KLZGFFTSSGTPIKNZLKVDELFSAILLLLQVAVVKIEAHTCRTELESQDNALADVYAES
GSAETVKICNLNELHKINPSQLPYDDLFNKQCNASHLEKQNWYLKGCKFNVKQTHSPDSC
LVLPESLKLPLLKALHSTTHHGTDKMIQLEKNTGGVTSKTAKMVYNQCLTCQSNNPGKTI
KVSGNICLPPDGVFZHLQIDFIQLSPSMGYQYILAIVCMFSGWIEAFQCSKADAVKKLEN
LFPFLGDPEKSSSDRGTHFIGQVIKQLNKVLPMQRHYHCPYHPHSSEKVERTNVILKLQL
AKLTESIGLPWPKVLPLALMAIRSTFIGKHKLTPYEIVTGRPHASPALLNSDITKYCKAL
MHYAKVYFCQVKETFZDPLTEQSDPPHGLEPGDWVFWKQYQRKTIFEPCWKGPYQVLTTH
TAVKLQGLEPWVHVSQLKRTPSDLWNCTPVGDFDKVDQGNLSPGRDPS
>Xen1
QTEAISLCSVPLLLFTDLTAISDCNIPAHVLSRIPPVLWSQGPEDIGRLRVPPVAVCLKE
GAVLPRKPQYPLKPAQSESINKQLQTLLENGAIKRQSSPCNTPLFPVKEKGKAGEPDKYR
LVQDLRAVNEATVMETPLVSNPHTILSGIPPSATHFRAVDLTNAFYSIPLREDCQYLFAF
THERQQYVWTVLPQGAQNSPTHFSLALTSILDSWISSHPEITLLQYVDDLLLCAPDLPTC
EASSTDLLSFLADQGCKASKEKLQWCQTTVVFLGQCISQGTRHITEGRIKTLQDIPLPKG
HKPLHAFLGLISYCRSWIPEASLLMQPLYDVLKSDPFTMTPAAEDSFHTLKSILSIAPAL
GLPDYEKPFKLFVSERQGHALGVLAQSYGQRIRPIGYFSGQLDNVAKGSPSCMRAVYAAR
LLLDKTADLILGHECTLLAPHDIAAILNQTQPKHMSAARPTAQCAVLLPDNVTLQRCTIL
NPSTLLPIPEPGGVEGHAVDFHDCFELMQQETAHLPTVSDTALDNPDLTLFVDGSRFSDA
SGKFHTGYAVTTTDSVLEAQPLPASCSAQEAELKALTAACKLAAGKRANIFSDSRYAQGV
ALDFGTIWKTRGYLTATGSPIKNGRSVADLMEALTLPEQVAVLKVKAHGRLTSPEAIGNH
LADTTAKEIAVAPLPDAPPLTTSTPLSKLHSWTVLTNYKLAKPQPAPKKSTDGSARGQLV
KTAYSCNKKPCIPRSLYPSLVQWAHGPTHVSKNLMNNLISKLYFAPGITTLTRNYTAACT
ICAQCNPGRMEKPPVLNLAKPLYPFQRIQIDHIQMPRCGRFEYVLVVVDMFSGWPEAFPV
ANMTAKTTAKKLLSEIVCRYGVPEVIESDQGPVFTASVTKDIWTALGVTLHFHTPYHPQS
SGKVERMNGTLKTKMLKMSQDSGMLWPDSLPIALFSVRYTPRGVNNLSPFEILFGCAPRL
GCYFPQTLQLQFDVLNEYVCQLSNELSKVHGQVFSSIPDPTSVEGSHSLVPGDWVLVKKF
LRKSSLEPRFDGPFQVLLTTATSVKLDGKNTWIHASHCKKSAPPASPASDSDSAPDSILP
ASP
>ALV
TVALHLAIPLKWKPDHTPVWIDQWPLPEGKLVALTQLVEKELQLGHIEPSLSCWNTPVFV
IRKASGSYRLLHDLRAVNAKLVPFGAVQQGAPVLSALPRGWPLMVLDLKDCFFSIPLAEQ
DREAFAFTLPSVNNQAPARRFQWKVLPQGMTCSPTICQLVVGQVLEPLRLKHPSLRMLHY
MDDLLLAASSHDGLEAAGEEVISTLERAGFTISPDKIQREPGVQYLGYKLGSTYVAPVGL
VAEPRIATLWDVQKLVGSLQWLRPALGIPPRLMGPFYEQLRGSDPNEAREWNLDMKMAWR
EIVQLSTTAALERWDPALPLEGAVARCEQGAIGVLGQGLSTHPRPCLWLFSTQPTKAFTA
WLEVLTLLITKLRASAVRTFGKEVDILLLPACFREDLPLPEGILLALRGFAGKIRSSDTP
SIFDIARPLHVSLKVRVTDHPVPGPTAFTDASSSTHKGVVVWREGPRWEIKEIADLGASV
QQLEARAVAMALLLWPTTPTNVVTDSAFVAKMLLKMGQEGVPSTAAAFILEDALSQRSAM
AAVLHVRSHSEVPGFFTEGNDVADSQATFQAYPLREAKDLHTALHIGPRALSKACNISMQ
QAREVVQTCPHCNSAPALEAGVNPRGLGPLQIWQTDFTLEPRMAPRSWLAVTVDTASSAI
VVTQHGRVTSVAAQHHWATAIAVLGRPKAIKTDNGSCFTSKSTREWLARWGIAHTTGIPG
NSQGQAMVERANRLLKDKIRVLAEGDGFMKRIPTSKQGELLAKAMYALNHFERGENTKTP
IQKHWRPTVLTEGPPVKIRIETGEWEKGWNVLVWGRGYAAVKNRDTDKVIWVPSRKVKPD
VTQKDEVTKKDEASPLFAGISDWIPWEDEQEGLQGETASNKQERPGEDTLAANES
>gg01-chr1-156168845
RATVYHRALHFAIPLHWKREASPVWVDQWPLPTDKLAALTQLVSRELEAGHIEPSLSRWN
TPIFVIRKPLGSFRLLHDLQAVNAQLVQFSPVQQGDLSLAAVPRGWPLMVIDLKDCFFSI
PLAEQDREAFAFTVPVRNNQGPAQRFQWKVLPQGMACSPTICQLVVNTIIAPVRRDMPDC
QIVHYMDDLLLAAPTGSQLQALEARVSGALTGAGFTISQEKVQRGPGIEFLGYKFGSSTV
IPEGLEIKPHVTNLWDVQKLVGAIQWVRGALGIPPRLMKALYDQLKGSDPRERREWTPEM
```

DAAWQEILSLCSTASLSRWTPGIPLEGAVTRCQDGAVAVIGHALGSCPQPLRWLFPAVRA
FTPWVELLSLLLSKARTAAFRDFGKDLDIVHLPRFFRDSQILPDEILLALHGMGGKLRYA
GSLPIFELARPLWVSLRLRVVASPLDGPTVFTDASSLTGQGAVVWKNQTGDWEIRTFQDH
GVSVQTLEAWAVAMALLLWLETPCNVVTDSAFIAKMLLRMGQEGPIPPHYCGSPQFNDTD
LANETVDNVGKKVACVICRLNVSLPWEPQELLLLGSQGVPNDTWVNTTWLSPGCIGFAKV
PNHNYSLLDSISQKGPYWYSRINRSDPFTDVFPRDRTHITGEKSIAVTRQTKPHSCCLTT
APGIWISVDTTVTIARPDGFPRNLTCSGYNAGGSNTSGCCVGPALRGQGEGRTSGIMTQL
KHCRQVFICGDHAWQGIPVKLVGGPCYLGQLTVLSPKVSEWLQIMNRTRMRTKRDLQQLS
PNCSDEVRLWGMTARVFASLFVPIGAAQVLKEIERLACWSVKQANATTLVLNEMLEDMNS
IRRALLQNWAAIDFLLLAQSHGCEDVEGFCCFNLSDHSASIHKQLQWMQGHTQKIKVLAD
PFGEWLESLFGELEPWLKQMLKTLIVGLAIFLAIMLSSMLCSAS
>MPMV
LTAAIDILAPQQCAEPITWKSDEPVWVDQWPLTNDKLAAAQQLVQEQLEAGHITESSSPW
NTPIFVIKKKSGKWRLLQDLRAVNATMVLMGALQPGLPSPVAIPQGYLKIIIDLKDCFFS
IPLHPSDQKRFAFSLPSTNFKEPMQRFQWKVLPQGMANSPTLCQKYVATAIHKVRHAWKQ
MYIIHYMDDILIAGKDGQQVLQCFDQLKQELTAAGLHIAPEKVQLQDPYTYLGFELNGPK
ITNQKAVIRKDKLQTLNDFQKLLGDINWLRPYLKLTTGDLKPLFDTLKGDSDPNSHRSLS
KEALASLEKVETAIAEQFVTHINYSLPLIFLIFNTALTPTGLFWQDNPIMWIHLPASPKK
VLLPYYDAIADLIILGRDHSKKYFGIEPSTIIQPYSKSQIDWLMQNTEMWPIACASFVGI
LDNHYPPNKLIQFCKLHTFVFPQIISKTPLNNALLVFTDGSSTGMAAYTLTDTTIKFQTN
LNSAQLVELQALIAVLSAFPNQPLNIYTDSAYLAHSIPLLETVAQIKHISETAKLFLQCQ
QLIYNRSIPFYIGHVRAHSGLPGPIAQGNQRADLATKIVASNINTNLESAQNAHTLHHLN
AQTLRLMFNIPREQARQIVKQCPICVTYLPVPHLGVNPRGLFPNMIWQMDVTHYSEFGNL
KYIHVSIDTFSGFLLATLQTGETTKHVITHLLHCFSIIGLPKQIKTDNGPGYTSKNFQEF
CSTLQIKHITGIPYNPQGQGIVERAHLSLKTTIEKIKKGEWYPRKGTPRNILNHALFILN
FLNLDDQNKSAADRFWHNNPKKQFAMVKWKDPLDNTWHGPDPVLIWGRGSVCVYSQTYDA
ARWLPERLVRQVSNNNQSREZFSPZDRSFPCSQIZTSIIISSGAZZYYLKYLKFKPVLEI
RVKPWQKYNKNMVNLVTVLEDMFPPHRLTLLQLFLALLILLIQZQTPZNGSVCQLPLPLA
IHIZEVVPVNATRSHMILYMPLAITTINNVTLVIKHISLPLZLEIELLPLVTGMSLQYZG
LVTTSLQQAVPMVKRAKWSVGIADLLFIYLMEEGLKIRPATLZZIKSLRNCTGRCSQNFL
TILWPCPKPVVKKKLTHTLLISLPLYIVYSMLPNPVZPKIAGCAYSQEIPFLLPCPIMIH
SALTLPVYLITPALZPPLFZYSPLTSLIPIAFTLIIKTTHLTZMZVZLALLIALAIITFL
QPPNPLIPYAPQTARFLYAVTIRHTLIYPQIGREVVYLLLFCPIZTSFQVVSLSPFQLLI
IFZAKPKEQSNLSPCSZGZVZLLQYLLGLLVZGFPSLNIQNYLINZYQMFKLFLALYKIS
KIRZTLZQKZYCKTEEDZIYLQQSREVSAZPYRKNVVSTPINLESSETRLKTYKTTZKDA
EDNZSTTHFGPVFMDSSLMLCPYZALCFAYCLCYLSVQLFSTSLZPLLNIKLRASRPNLY
KSIIIALNKKTVVAHIZPZHRPPPLZARLDSQZRVREZHFSLTZDRRAVKATAZSNDGZZ
ZQEMYHSNLRQAQPPRDVSFVFYNZKGZHVRSRAARMMSWPLFALAPCYEFKMAYFLVLL
RLTFPPARMFPALGLTWLSLLCYZACAQYLSPPTCCLCIZGNTLPPLNETZSEHCLVSIS
CVSCSLQFPLPPPGSYCZSRGSGQFSFSVRSRAARMMSWPLFALAPCYEFKMAYFLVLLR
LTFPPARMFPALGLTWLSLLCYZACAQYLSPPTCCLCIZGNTLPPLNETZSEHCLVSISC
VSCSLQFPLPPPGSYCZSRGSGQLAPNVGHEX
>JSRV
MMHMGALQPGLPTPSAIPDKSYIIVIDLKDCFYTIPLAPQDCKRFAFSLPSVNFKEPMQR
YQWRVLPQGMTNSPTLCQKFVATAIAPVRQRFPQLYLVHYMDDILLAHTDEHLLYQAFSI
LKQHLSLNGLVIADEKIQTHFPYNYLGFSLYPRVYNTQLVKLQTDHLKTLNDFQKLLGDI
NWIRPYLKLPTYTLQPLFDILKGDSDPASPRTLSLEGRTALQSIEEAIRQQQITYCDYQR
SWGLYILPTPRAPTGVLYQDKPLRWIYLSATPTKHLLPYYELVAKIIAKGRHEAIQYFGM
EPPFICVPYALEQQDWLFQFSDNWSIAFANYPGQITHHYPSDKLLQFASSHAFIFPKIVR
RQPIPEATLIFTDGSSNGTAALIINHQTYYAQTSFSSAQVVELFAVHQALLTVPTSFNLF
TDSSYVVGALQMIETVPIIGTTSPEVLNLFTLIQQVLHCRQHPCFFGHIRAHSTLPGALV
QGNHTADVLTKQVFFQSAIDAARKSHDLHHQNSHSLRLQFKISREAARQIVKSCSTCPQF
FVLPQYGVNPRGLRPNHLWQTDVTHIPQFGRLKYVHVSIDTFSNFLMASLHTGESTRHCI
QHLLFCFSTSGIPQTLKTDNGPGYTSRSFQRFCLSFQIHHKTGIPYNPQGQGIVERAHQR
IKHQLLKQKKGNELYSPSPHNALNHALYVLNFLTLDTEGNSAAQRFWGERSSCKKPLVRW
KDPLTNLWYGPDPVLIWGRGHVCVFPQDAEAPRWIPERLVRAAEELPDASDATHDPE
>MMTV
MIGAIESNLFADQISWKSDQPVWLNQWPLKQEKLQALQQLVTEQLQLGHLEESNSPWNTP
VFVIKKKSGKWRLLQDLRAVNATMHDMGALQPGLPSPVAVPKGWEIIIIDLQDCFFNIKL
HPEDCKRFAFSVPSPNFKRPYQRFQWKVLPQGMKNSPTLCQKFVDKAILTVRDKYQDSYI
VHYMDDILLAHPSRSIVDEILTSMIQALNKHGLVVSTEKIQKYDNLKYLGTHIQGDSVSY

QKLQIRTDKLRTLNDFQKLLGNINWIRPFLKLTTGELKPLFEILNGDSNPISTRKLTPEA
CKALQLMNERLSTARVKRLDLSQPWSLCILKTEYTPTACLWQDGVVEWIHLPHISPKVIT
PYDIFCTQLIIKGRHRSKELFSKDPDYIVVPYTKVQFDLLLQEKEDWPISLLGFLGEVHF
HLPKDPLLTFTLQTAIIFPHMTSTTPLEKGIVIFTDGSANGRSVTYIQGREPIIKENTQN
TAQQAEIVAVITAFEEVSQPFNLYTDSKYVTGLFPEIETATLSPRTKIYTELKHLQRLIH
KRQEKFYIGHIRGHTGLPGPLAQGNAYADSLTRILTALESAQESHALHHQNAAALRFQFH
ITREQAREIVKLCPNCPDWGHAPQLGVNPRGLKPRVLWQMDVTHVSEFGKLKYVHVTVDT
YSHFTFATARTGEATKDVLQHLAQSFAYMGIPQKIKTDNAPAYVSRSIQEFLARWKISHV
TGIPYNPQGQAIVERTHQNIKAQLNKLQKAGKYYTPHHLLAHALFVLNHVNMDNQGHTAA
ERHWGPISADPKPMVMWKDLLTGSWKGPDVLITAGRGYACVFPQDAETPIWVPDRFIRPF
TERKEATPTPGTAEKTPPRDEKDQQESPKNESSPHQREDGLATSAGVDLRSGGGP
>HML1
KPPEPIPLTWKTQKPVWVDQWPLPKNKLEALHILVLAQLKLGHIEPSFSPWNSPVFVIQK
KSGKWRMLTDLRAVNAVLQPMGTLQPGLPSPTMIPEYWPLIIIDLKDCFFTVPLAPQDFE
KFAFTVPTLNNVAPAARYHWKVLPQGMLNSPTICQYYVGCILKPVRDKFPQCYIIHYTDD
ILCAAPSRSVLISCFSALQQAVTAAGLVIAPEKIQTSSPYHYLGMQLEDKVIKPQKVQLR
RDSLKTLNDFQKLLGDINWIRPSLGIPTYAVSNLFATLRGNPDLRSKRSLTPEADSELRL
IEKCIQQSQVTRVNPHLPFEILIFPTEHSPTGLIIQGHNLIEWCFLPHSSLRMLTIYLDQ
ISTLIGQARSHLLRLSGTEPQKIIVPLTRLQVQQAFATCIAWQVHLAGFPGIIDNHCPNV
KLFQFLKLTSWILPNVTRNTPLPEAVTVFTDASSNGRAAYTGPRERVLNTGAISAQRAEL
LAVRAVLEEFPEPVNIVSDSAYVVHVAHNIETALIQFMPDDTFLFQKFQSVLRARSSPFY
ITHIRAHTPLPGPLSAANARADTLVTPIFTDAENFHALTHVNAAGLRNKFPLTWKQAKII
VRHCPTCQVLILQPLSSGVNPTGLSQNALRQMDVTHYPPFGKLSFIHVTIDTFSHFIWAT
CQTGESTAHGKRHMLSCFSVMGCPEKLKTDNGPGYTSAAFKKLPQTWAIAHTTGIPYNSR
GQALVERAKKTLKDQLRKQDTKLKGDAVTPHAKLNLALFTLNFLNLTRNQPFTAAEQHFT
VNKFDPQKGMRVWWKDVKTNTRELGTVITWGRGFACVSPGKGLQPVWVPSRQLKLHHNSK
DETLPETKGKDPSETKEQLSPPDTYPS
>HML2
KSKKRRNRVSFLGAATVEPPKPIPLTWKTEKPVWVNQWPLPKQKLEALHLLANEQLEKGH
IEPSFSPWNSPVFVIQKKSGKWRMLTDLRAVNAVIQPMGPLQPGLPSPAMIPKDWPLIII
DLKDCFFTIPLAEQDCEKFAFTIPAINNKEPATRFQWKVLPQGMLNSPTICQTFVGRALQ
PVREKFSDCYIIHYIDDILCAAETKDKLIDCYTFLQAEVANAGLAIASDKIQTSTPFHYL
GMQIENRKIKPQKIEIRKDTLKALNDFQKLLGDINWIRPTLGIPTYAMSNLFSILRGDSD
LNSKRMLTPEATKEIKLVEEKIQSAQINRIDPLAPLQLLIFATAHSPTGIIIQNTDLVEW
SFLPHSTVKTFTLYLDQIATLIGQTRLRIIKLCGNDPDKIVVPLTKEQVRQAFINSGAWQ
IGLANFVGIIDNHYPKTKIFQFLKLTTWILPKITRREPLENALTVFTDGSSNGKAAYTGP
KERVIKTPYQSAQRAELVAVITVLQDFDINIISDSAYVVQATRDVETALIKYSMDDQLNQ
LFNLLQQTVRKRNFPFYITHIRAHTNLPGPLTKANKQADLLVSSALIKAQELHALTHVNA
AGLKNKFDVTWKLAKDIVQHCTQCQVLHLPTQEAGVNPRGLCPNALWQMDVTHVPSFGRL
SYVHVTVDTYSHFIWATCHTGESTSHVKKHLLSCFAVMGVPEKIKTDNGPGYCSKAFQKF
LSQWKISHTTGIPYNSQGQAIVERTNRTLKTQLVKQKEGGDSKECTTPQMQLNLALYTLN
FLNIYRNQTTTSAEQHLTGKKNSPHEGKLIWWKDNKNKTWEIGKVITWGRGFACVSPGEN
QLPVWIPTRHLKFYNEPIRDAKKSTRGDGDTAIEHR
>HML3
KWLTDKPIWIEQWLLSKDKLEALEKLVTEQLENGHIAPTFSPWHSPVFVIKKKSGKWRML
TDLRAINVIQPMGALQPGLPSPAIIPKNWPLIVIDLKDCFFTFPLAKQDCEQFAFTIPAV
NNLQPAKHYHWKVLPQGMLNSPIICQTYVGQAIEPTRENFQCHIIHYMDDILCAAPTREI
LLZCYDHLZNLISRAGLIIAPDKIQTTTPYSYLGNLVNDTTIVPQKVTIRGDQLKTLNDF
QKLLGDINWIQPALGIPTYAMSNLFAILRGNPSVTSPWQLTKEAEAELQVIEKQVHKAZI
NRIDPEKTLDLRIFSTQHSPTGVIVQEQDLIEWLFLPHTNSRTLTHLNQIAIMIGIGRTW
IVKZHGYDPGKIIVPLTKAQIQQFVNSLTWQTHLTDFVGSLNNHFPKMKLFQFLKLTNWI
LPKITKFKPIEGAENVFTDGSSNGKASYFGLKGKVFQTPSAQKVELVAVIEGLTAFDMPI
NAISNSSYVVHSTQLIENAQLRFHTDEQLMTLFTQLQTVVRSRMHSFYITHIRAHTPLPG
PLTEGNQMADRLIANAISNARHFHNSTHVNASGLQCRYSITWKEAKAIIQZCPTCQMVHS
SSFTGGFNPRGLEPNSLWQMDVTHVPSFGRLAYVPVCVDTFSHCVWTTCQSGESSACVKC
HLQCFVVMGIPASIKTDNATGYTNQALATFFSVGNIKHITGIPYNSQGQAIVERMNLSLK
QQLQKQKGRDREYGTPQIQLNLALLTLNFLSLPKGQKLSAAEQHLQKLAAKTETEKLIWW
RDPITKSWEIGKIITWGRGYACISPGQNQQLIWILSRHLKPYHEPDAKEATPGDPEDPSV
AAMLRLTLGGP
>HML4
KWLTDKPIWMEQWPMSKEKLEALENLVTEQLEKGHIAPTFSPWNSPVFIIKKKSGKWRML

TDLRAINSLIQPMGTLQPGLPSPAMIPKNWPLIVIDLKDCFFTMPLAEQDCERFAFPIPA
VNNLQPAKCFHWKVLPQGMLNSPTICQVYLGQAIESTCKKFSQCYIIHYMDDILCAAPTR
EILLQCYDHLQNLISHAGLITAPDKIQTTAPYSYLGTLVNDTTIVPQKVTTHRDQLKTSN
DFQKLLGDINWIZLALGIPTYAMSNLFSILRGDPSLISPRQLTKETEVELQLIEKQVHKA
QINRINPKKTLDLLIFSTQHSPTGVIVQEQDLVQGLFLPHTSSRTLTPYLDQISTMIGNG
RTQIDKLHGYDPGKSIVPITKAZIQQAFINSLTWQTHLANFVGILDNHFPKMKLFQFLKL
TNWILPKISKFKPIKGAENVFTDGSSNSKASYSGSKGKVFKTPYMSAQKAELVAVIKVLT
AFNMPINVTSDSSYVVHSTQLVKNAQLZFHTDEQLMTLFTQLQKAVRSRMHPFYITYIRA
HTPLPEPLTEGNQMADHLVATAISNARHFHNLTHVNASGIKHRYSITWKEAKAIIQZCPT
CQMVHSSSFTGGVNPRGLEPNSLWQIDATHVPSFGRLAYVHVCVDTFSHFFWATCQSGEF
SACVKYHLLQCFVVTSISASIKTDNASGYTRQAVATFFSIWNIKHITGIPYNSQGQAIVE
ZMHLSLKRQLQKQKAGNRDYGTPHMQLNIALLTLNFLSLPKGQMLSAAEQHLQKPAAKTV
AEQLVWWRDPITNSWEIGKNIITWGRGYACVSPGQNHQLIWIPSRHLKPCHEPARSWGPP
GCSHV
>HML7
QWMCESPVZVEQWPLFKHKLEALIEIVNDLLQANTIEPSLSPWNSPVFVVQKKSGKWRMV
TDLRAVNAVIKPMGALZPSMPSPSMIPKEWPLIIIDLKDCFFHIPLDKSDCEKFAFTIAS
INNSASAARYQWKVLSQGMINSPTICQFVGTVLQPIRQTFKNNYILHYMDDILIAAPTKD
ELIQCFTSIEZAVANAGLNIAPDKIQQATPFLYLGMQLEARSTKPQKVQLHTTLNTFNDF
QKLLDINYLRPTLGIPTYALSHLFATLSGDSDLNSPHSLTEPAKQELSFVEZVREAQISC
IDPNLPLQFLVFPSIHSPMELIVQNDSLVEWVFLPNSASKTLSIYLDQMATSFDLVIRQR
ITKISSFDPDIFVVPLSKNEVKNAFSTSLCWQTNLADFIGTIDNHLPKSKFFQFLZNTSW
TLKKLTRSSPLEAATTIFTDGSSNGKARYVGPKDKVISTPYTSAQKTZLFAVISALQDFD
QPLNIVSDSAYVVHATKAIETATIKNITDTNIFSLFSLLQKTTRNZNHPFFITHIRSHTN
LPGPLSSGNHKVDTLVSLAIIDAEQFHQLTHTNVSGLKHKYSLSWKQPKQIVQHCSQCQV
LVLPTQSPGVNPRGLSPNAIWQMDVTHVPSFGKLAYVHVTVDTFSNFIWATCQTREAISH
VKKHMFSCFAVMEIPGELKTDNGPAYCSKAFKNFPDQWHIKHVTGIPYNPQGQAIVEKSN
RTLKLQLLKQKEGDKELSTHHIQLNLALLTLNFLNIPKSSSVTAAKKTFLWZPSHSKPRK
GSMVKDVQSTICSKVSILMWGRGYACVSPGEHQSPVWIPTRHLKLWPEDACKKETEKFAE
KTPEQETPNTSNHQKEKVTM
>HML8
QWKSDIPIWIZQWLLSKEKLEALTQLVSEHLQLGNVESSLFSWNSPVFLVKKKSGKWRMV
TDLRAINAVIKPMGAVQPGIPAPALIPKNWPLIVIDLKDCCFHIALHKSECEKFAFTIPS
INNQEPAARYQWKVLPQGMLNSLTICQLHVGQVLSPVRAQFPKPYILHYIDDILIAAPTD
KELIDCYQILSRCVTEAGLHITQGTIQQTTPVQYLGMVVDKQCIRPQKIQIRRDSLKTLN
DFQKLLGNINYLRPNLGIPTYVLSNLFSMLLGDSDLRSPWTLTPEALLELEFVEERIQTT
QLSRVQPFQPFQLLVFASLHSPTGLIVQHNDLVEWCFLPHSVLKTLCVYLDQIAILIGQA
WCRILQISGFDPNVIVVPLNQLKVQAAFQHSVLWQIHLADFIGIIDNHYPKNKLFDFIKM
TSWVVPKLTKDQPIPKAITVFTDGSSNGNAVIGPTDKLISTSYTSAQTAELIAVITALQD
FPKPLNIVSDSTYVVHATKNIETATIKHIDNSELASLFSRLQHAVCQHRHPFYITHIRSH
TTLPGPMSAGNHKVDCLVSFTIQEAQEFHNLTHVNAAGLKDKFALSWKQAKFIVHSCPQC
QVFVLPNQGPGVNPRGLTANALWQMDVTRASSFGGLSYVHVSVDTFSGFIWATCQTGEGM
THVKKHLYSCFAVLGLPYQIKIDYTAGYVSKAFDSFMQQWGISHITGIAYNPQGQAVVER
ANRTLKTQLSKQSEQPKHDLTTPHSQLHLALFTLIFLNVPKDNTLTAAKRHYTGKKFSLN
EDKPVLWKNSQTNTWEPGTIITWGRZYTCVSPGDHQSPVWVPTRRLKLQVNLTMKTTEKR
RLCQRLPSDW
>HML6
RXSGFLMGVIDISPPPXALPLEWLSDKXXWVDQWPLXQEKLXQLQQLVKEQLDAGHIEES
XSPWNSPVFVIPKKSGRWRLLHDLRAINAQIKPMGXLQQGLPSPXAIXRDWPLVVIDLKD
CFFTIPLHEKDKPRFAFSVPSINQREPVSRYQWRVLPQGMLNSPTLCQHFVGQALKEPRN
MFPTAYIIHFMDDILLAAPTDQILHQLFRETKQAXTKWNLKIAPEKVQTTSPYHYLGTIV
XERSVXPQKVVLCKDRLQTLNDFQQLLGXINWLXPMLGIXXYQLTHLXQTLQGDSSLNSX
WQXXKEAEXELXLVEQMXQQRHAXQLXXQKXLLXFILPXPHSPTGXLGXXXDXSVTVIEW
LFXPNQTXKXXQVYLSLITQIVTMGXHRSKMLMGYXPXKIIVXLDSQQXXXAWEMXXXWQ
IAFAXFVGXIDXXXPSDKIXQFYKXHSFILPVITHHKPIXGGXTYFTDGSSKGXXAXYGP
KHTQXIMXSGVSAQXSEXIAVIQVLXLTASDPINIVCDSAYVVNVASHIETATIKSTLXP
ELLNLFLRLQQAIXSRAAPFHISHIRSHTQLPGPLSLGNDRADKLIGSVFQQXQASHALL
HQNTSALTRMFHLPXSQAXAIVQACPTCQHVPGVAPVEGCNPRXLAPNEIWQMDVTHIAA
FGKLSYVHVTIDTYSHMLHATCQTGXTAGHXQXHCLSSFAHMGXPKQLKTDNGPAYVSHA
FQNXLQLWAITHKTGIPYNPRGQGIIERAHQTLQXMLKKQKGGIGDQLPPQTKLHLALFT
LNFLTPGTDSKTPAEXHWQMLEGKRKVYPKVLWKSXEEGQWKGPVDLLMWGXGYACVFTG

DGQTVWVPSRCVRPWNGRLEEPMVANHGPVPPV
>HML9
NSPVFVIPKKSGKWGLLHDLRAINAQIKPMGALQQGLPSLAAFPRDWPLVVIDLIVFFTI
LLHQQDRPGYAFSVPSVNQREPVSCYQWKVLPQGVLNSPTLYQNFVGQALKEPCNVFPTG
YVIHYMDDILLAAPTDQILHQLFRETKQALTKWNLKIAPEKVQTTSPYQYLGTIITERSV
QPQKVVICRDRLQTLNDFQQLSGDINWLHPMLGIATYZLKHLYQTLQGDSSLDSPRQLTK
EAEAELZLVEQMLQQQHATWLQPQKPLLLFILPTSHSPTGLLGQFIHKSVIVLEWLFYPI
TVKSLQIYLSLITQLITIGRHRSKMFMGCDPDKIIVPLDSQQQATAWEMSTAWQITLTDF
VGIIDNHYPSDKILQFYEVHPFTLPVITHHKPIPGRQTYFTDDSSKGCAAIYGPKHTETI
KTSGVSAQCSELVAVIQVLQLTALSPINIVCDSAYIVNVASHIETATIKSTLEPELYNLF
LRLQQAVHSHAAPFHISHICYHTQLPGPLSLGNNKADKLIGSVFQQAQSSHAFLHQNTSA
LTRMFHLPCSQAZAIAQAYPTCQPVPGVAPMEGCNPLGLAPYEIWQMDVTHIAAFGKLSY
VHVTTDTYSHMLHVTCQTGETAGHVDHCLSPFAHMGVPTQZKTDNGPAYVSHAFQNFLQL
WAITHKTEIPYNPQGQGIIEWAHQTLQCMLKKQKWGLGDQLPPQTKLHLALFTLNFLTPG
MDIKTLAEZHWQMLEGKRKVYPKVLQKSPEEGQWKGPVDLLIWGRGFACVFTGDGQSVWV
PSRCVZPWNGRLEKPWISTVGPAPPVRAMS
>HML5
VMATVIIPPLPLMRLSQDPIWVDQWPLKGEKLQRAHEFVEEQLKASHIEPSKRPWNSPIF
IIPKKSGKZRLLHDZHAINANLKPMGPLEQVFSSPSVIPZDWHIIVIGLKYCICTIPFAE
QDREKFVFIIPAINNERPAPRFHWKVLPEGMLNSPTMCQYHVNQALLLSRKEFPNCKTIH
FMDDILLAAPMDPALLSLCASVVKNTQLRGLIIAPEKVQMSSPWKYLGYILTSWSVRPQK
VKLNTSNLHTLNDYQKLLGNINWLCPTLGITTDKLQNLFSILKDNAALDSPRYLTPAAQR
EIEEVEQAISQRQLDHIDPWYSVQLSVFPTKHSPTGLVGQMAPELRFLEWVFLLHTGTKP
FSPYIQLVSKVIYSGHRRCNLLLGYDPDIIRIPLSKKQCEAVLPLYIDLQTARSDYTGHI
EPALPADKLLQFLSCTSVVLPKIVQSPTPNALTLFTNGSGKHGKVAVWWRLHNSLTHSGF
NSTQRAEVGPLILTLETFTQPINIVSDSAYSVHLLQNLETALIKSTLEPTLCALFLZLQH
LLDQHTHPIFITHIRAYSZLPGPLAYGNDQADLQVCQSHQFFHQGWRNLSKQFQLTQRLA
KQIILQCPDCQLIGTSPPSTGVNPRGLEPNQLWQTNVTHIPEFGKLRYVHISVDTNSHLI
SACAIPGESTQYVIKHLLLTFACMEHPTKIKTDNGLAYASSZFQQCHHIZNIQHSAGILY
NPQGQAIVDHTHSTLKNMLRKQKRGNMSKDPATPLAQALFTLNFZNLNDKFQSAVEKHFA
KTSQDIKPAVLWKDVNSNVWRGPNELLTWGRGYACVHTPSGPLWIPRQHFEPYHGMARTQ
PSTRNEENDPAGPTALDDAASSDDTSPGHYLGDAEEDNSGG
>Python-molurus
TNLWGRDLMSQFQTKLILQLMAVSAVLRLELQSQTPVWIDQWPLTVEKLTVLKQLVNEQL
DNGHLEPSLSPYNTPVFVIKKKSGKWRLLHDLRAVNAIIKPMGALQCGLPNPNLIPQSYD
MMIIDLKDCFFSIPLHPKDKHIFAFTVPALNNSQPTSRYQWKVLPQGMLNSPTMCQYFVS
QVLQPYRDKYSSFLVYHYMDGILVATPGPKGTVLKTLPHLQRILQQGGLHIAPDKVQLSS
PFHYLGHKVLESHANPCLPNIQLPPAPTLLQLQQYLGSINWIRPYIGLSTIQLSPLFTAL
TKGKQPADVIPLQKEQLDAVKLINQALRTKWVDRMKDNLPFSLLLLNTLTLPTGAVVQVT
PSLLILEWIHLSHTPRATLTTKPAQFASLITKGRLRSKALAGVEPSVIYVPCSLSTWESL
LAVSDHLQFALADWPGTVSCHPPPDPRVQLLRTTPFIWQTPMSPFPIHDAVTVFADGSKK
SGACAWQIDGQWHTKTTPPQSSAQRAELYASILAFEAFPHQSFNLILDSFYVSQIISSIY
QAYLSPSIEPTLLSLFLSLQHLISKRTHFFHVAHIRSHQPFPGILQEGNSVADAAAAAAS
SSPLIFAFSSPTPWESHSYFHQNKKALIKQFKISATEATAILQQCPTCSKQAHSVPMGVN
PRGTECNQIWQMNVTQYASFSPWKYLHVSIDTYSGFIMATPQRDEASKRIINHCIRTFAT
LGRPKELKTDNGPGYTGSAFAAFCQKWGITHKFGIPYNSKGQAIIGRANQTLKHALDRYI
ETKGKGPPSLPAVQDILNLCLYTLNFLNLTGLPATSAACRHFASQPAADHQRPLVYYRCL
PDPAWRGPAQLITWGRGYAAIQLPDKVLWVPGRCVRPCHLPLSPDAQDVPPPPHIRGLTG
V
>gg01-chr7-5733782
PICLTWITDKSVWIEQWPLKKVNLDNVIKLVAKQLQKGHIQPSTSPWNTPIFIIKKKSGK
YRLLHDLRVVNAQMQPIGALQPGLPNPTMIPENWHLLIVDLKDCFFTINIHPKDTSRFAF
TVPATNKGAPAARYEWTVLPQGMKNSPTLCQLFVDVALEPIRKAWSHAVIYHYTDDILIA
ZSRPFTTDQELYLKRTLQRKGLVIAPEKVQREAPWKYLGWVIAQSHVCPQKLTLHTDIQT
LNDTQKLLGDLQWLWPVVGLLNDDLNSLRPLLKGTDPAACISVSPEQQQSIEHLAQTVVE
RSVDRRDPSLPIDITVLLGZTQLLAALTQRKKKKGEQADIRVLEWLFTALQPRTTIQQTI
DNLAELVHRGHKRVLSIAGEEPGTIYLLVKHTDLDWYIQNSTELASSLLSSGANLEICPL
ALPVLKWMTQRRWLVIPKLSRAPLQNAITVFMDAGRKSRKAVAVVRGDTGSYKQPGDSLQ
TLELSAVVWAFFNWPLQPLNVVSDSLYVVGLVERVEDTRVRELSNQSLYELLTTLQRVTL
QRAITIRRERYSIIHIRSHKWQEGLGEGNQRADDLVAVSVPVDRLVQAWEAHATFHQNAR
GLHKLYKISLNEARGIVRACPSCSNFSPGLGLEVNPRGLGPLEICQMDVTHIPEFSRLKY

VHVTVDTYSKFIWATVGEKVLHVIHHLTVCFAIMGLPKTFKTDNGPAYTSQGFGRFCEKW
GVQHITGVLNSPAGQAIVERANETLKRYVSRLSDVRDTQEHLARALFTVNYLCMYGEGEE
TPAQVHSRVPRVGNTQPVSVEYHNPRTGIWERPAEVKYMGWGYVCAYTDRSTMGSGQMD
>gg01-ChrU-163504869
PKLEHLLFLPVHCMMLZCGILITKNHKTVTLPDSPHPLLGQDLLEQLGAEVKFESGKMKF
KVKDDSFIKMLSLALITASEDSGIPEEIINQIYPGVWATEVPGHAKNASPIVIKVKQEAH
PPRIKQYPLRAEDREEIQLIIDZFIKYGLLVECESKYNTPILSVKKSDWSYHIVQDLRAI
NKIVDLYPLVANPYTLLTRLSNELAWFTVLDLKDAFFCLPLSPESQLLFAFEWENPKSER
RTQLTWTVLPQGFKNSPTIFGNQLAKGLEQWEHPSGKGVLLQYVDDFLIATETEELCIAW
TISLLHFLGLMDTVSPQKAQVAKQQAVYLGYGITAGLRTLGTARKEAICQTPEPRTAKEL
CMFLGMTGWCQLWIHNYGLLVKTLFALLKTNPNVLTWGGETRRVFKLLKHELMQAPALGL
PDTTKPFWLYSYEKQGIALGVLAQDLGPYRRAVAYFSKQLDEVSKGWPGCLRAVAALVLN
VQEARVEGWLHSQGGHGSFEQLEEQNRPNNWPSVGMLTDVTSLPIDITMLLGRTQLLAAL
TQREKKKGEQADIRVLEWLFTALQPRTTIQQTIDNLAELVRKRRKZVLSIAGEEPGTIYL
PIKRTDLDWYIZNSTELASSLLSSGANLEVRPLASPVLKWMTQRRWLVISKLSRAPLQNA
ITVFTDVGESHEKRWRRVLPSATVMLASCVSPLAHTDQLPDQGQMTADAHQYDAVKWRLF
TNTHSLYTLLELSCPLLIRPYWILTSYHNSLFGSYCLLIPSPSYLVYFVSKDSLSEQQHP
FTMLRERPIPYSTYPRKAPTTTTWCEGQQWRHRLLQAQPGDSLQTLELPAVVWAFLNWPL
QSLNMVSDSLYVVGLVERVEDARVRELSNQRLYKLLPYKGQAIRRERYSIIHIRSHKWQE
GLGEGNQRADDLVAVSVPVDRLVQAREAHATFHQNARGLHKLYKISLNEARGIVRACPSC
SNFGPGLGLGVNPRGLGPLEIWQMDVTHIPEFGRLKYVHVTIDTHSKFIWATVQVGEKVF
HVIRHFTVCFAIMELPKTLKTDNGPAYTSQGFGCFCEKWDVQHITGVPNSPTGQAIVERA
NQTLKRYVCRLSDVRDTQEHLARALFTVNYYVYLEKARKPLLSDTLTAIVQDLLVVPLLV
NSPZIPTAHLLSCRTVQASVKQSLQSSVRRRSWTGLAQPQLDLLHAVGAIEASDAQIMMN
NPNPILQALTSLKYLHKFSFFSFPSALTTFPSTFTCQQNIQQTLLNHSRGT
>HIV-1
TRANSPTRRELQVWGRDNNSPSEAGADRQGTVSFNFPQVTLWQRPLVTIKIGGQLKEALL
DTGADDTVLEEMSLPGRWKPKMIGGIGGFIKVRQYDQILIEICGHKAIGTVLVGPTPVNI
IGRNLLTQIGCTLNFPISPIETVPVKLKPGMDGPKVKQWPLTEEKIKALVEICTEMEKEG
KISKIGPENPYNTPVFAIKKKDSTKWRKLVDFRELNKRTQDFWEVQLGIPHPAGLKKKKS
VTVLDVGDAYFSVPLDEDFRKYTAFTIPSINNETPGIRYQYNVLPQGWKGSPAIFQSSMT
KILEPFRKQNPDIVIYQYMDDLYVGSDLEIGQHRTKIEELRQHLLRWGLTTPDKKHQKEP
PFLWMGYELHPDKWTVQPIVLPEKDSWTVNDIQKLVGKLNWASQIYPGIKVRQLCKLLRG
TKALTEVIPLTEEAELELAENREILKEPVHGVYYDPSKDLIAEIQKQGQGQWTYQIYQEP
FKNLKTGKYARMRGAHTNDVKQLTEAVQKITTESIVIWGKTPKFKLPIQKETWETWWTEY
WQATWIPEWEFVNTPPLVKLWYQLEKEPIVGAETFYVDGAANRETKLGKAGYVTNRGRQK
VVTLTDTTNQKTELQAIYLALQDSGLEVNIVTDSQYALGIIQAQPDQSESELVNQIIEQL
IKKEKVYLAWVPAHKGIGGNEQVDKLVSAGIRKVLFLDGIDKAQDEHEKYHSNWRAMASD
FNLPPVVAKEIVASCDKCQLKGEAMHGQVDCSPGIWQLDCTHLEGKVILVAVHVASGYIE
AEVIPAETGQETAYFLLKLAGRWPVKTIHTDNGSNFTGATVRAACWWAGIKQEFGIPYNP
QSQGVVESMNKELKKIIGQVRDQAEHLKTAVQMAVFIHNFKRKGGIGGYSAGERIVDIIA
TDIQTKELQKQITKIQNFRVYYRDSRNPLWKGPAKLLWKGEGAVVIQDNSDIKVVPRRKA
KIIRDYGKQMAGDDCVASRQDED
>HIV-2
PSGADTNSTSGRSSSGTVGEIYAAREKAEGAEGETIQRGDGGLAAPRAERDTSQRGDRGL
AAPQFSLWKRPVVTAYIEDQPVEVLLDTGADDSIVAGIELGDNYTPKIVGGIGGFINTKE
YKNVEIKVLNKRVRATIMTGDTPINIFGRNILTALGMSLNLPVAKIEPIKVTLKPGKDGP
RLKQWPLTKEKIEALKEICEKMEKEGQLEEAPPTNPYNTPTFAIKKKDKNKWRMLIDFRE
LNKVTQDFTEIQLGIPHPAGLAKKKRISILDVGDAYFSIPLHEDFRQYTAFTLPAVNNME
PGKRYIYKVLPQGWKGSPAIFQYTMRQVLEPFRKANPDVILIQYMDDILIASDRTGLEHD
KVVLQLKELLNGLGFSTPDEKFQKDPPFQWMGCELWPTKWKLQKLQLPQKDIWTVNDIQK
LVGVLNWAAQIYSGIKTKHLCRLIRGKMTLTEEVQWTELAEAELEENKIILSQEQEGYYY
QEEKELEATIQKSQGHQWTYKIHQEEKILKVGKYAKIKNTHTNGVRLLAQVVQKIGKEAL
VIWGRIPKFHLPVERETWEQWWDNYWQVTWIPEWDFVSTPPLVRLTFNLVGDPIPGAETF
YTDGSCNRQSKEGKAGYVTDRGKDKVKVLEQTTNQQAELEVFRMALADSGPKVNIIVDSQ
YVMGIVAGQPTESENRIVNQIIEEMIKKEAVYVAWVPAHKGIGGNQEVDHLVSQGIRQVL
FLEKIEPAQEEHEKYHSIIKELTHKFGIPLLVARQIVNSCAQCQQKGEAIHGQVNAEIGV
WQMDYTHLEGKIIIVAVHVASGFIEAEVIPQESGRQTALFLLKLASRWPITHLHTDNGPN
FTSQEVKMVAWWVGIEQSFGVPYNPQSQGVVEAMNHHLKNQISRIREQANTIETIVLMAV
HCMNFKRRGGIGDMTPAERLINMITTEQEIQFLQRKNSNFKNFQVYYREGRDQLWKGPGE
LLWKGEGAVIVKVGTDIKVVPRRKAKIIRDYGGRQELDSSPHLEGAREDGEMACPCQVPE

IQNKRPRGGALCSPPQGGMGMVDLQQGNIPTTRKKSSRNTGILEPNTRKRMALLSCSKIN
LVYRKVLDRCYPRLCRHPNT
>Visna
MPSLWKKRTYAKGLPAEETAGKQQEGATCGAVRAPYVVTEAPPKIEIKVGTRWKKLLVDT
GADKTIVTSHDMSGIPKGRIILQGIGGIIEGEKWEQVHLQYKDKIIRGTIVVLATSPVEV
LGRDNMRELGIGLIMANLEEKKIPSTRVRLKEGCKGPHIAQWPLTQEKLEGLKEIVDRLE
KEGKVGRAPPHWTCNTPIFCIKKKSGKWRMLIDFRELNKQTEDLAEAQLGLPHPGGLQRK
KHVTILDIGDAYFTIPLYEPYRQYTCFTMLSPNNLGPCVRYYWKVLPQGWKLSPAVYQFT
MQKILRGWIEEHPMIQFGIYMDDIYIGSDLGLEEHRGIVNELASYIAQYGFMLPEDKRQE
GYPAKWLGFELHPEKWKFQKHTLPEITEGPITLNKLQKLVGDLVWRQSLIGKSIPNILKL
MEGDRALQSERYIESIHVREWEACRQKLKEMEGNYYDEEKDIYGQLDWGNKAIEYIVFQE
KGKPLWVNVVHSIKNLSQAQQIIKAAQKLTQEVIIRTGKIPWILLPGREEDWILELQMGN
INWMPSFWSCYKGSVRWKKRNVIAEVVPGPTYYTDGGKKNGRGSLGYITSTGEKFRIHEE
GTNQQLELRAIEEACKQGPEKMNIVTDSRYAYEFMLRNWDEEVIRNPIQARIMELVHNKE
KIGVHWVPGHKGIPQNEEIDRYISEIFLAKEGRGILQKRAEDAGYDLICPQEISIPAGQV
KRIAIDLKINLKKDQWAMIGTKSSFANKGVFVQGGIIDSGYQGTIQVVIYNSNNKEVVIP
QGRKFAQLILMPLIHEELEPWGETRKTERGEQGFGSTGMYWIENIPLAEEEHNKWHQDAV
SLHLEFGIPRTAAEDIVQQCDVCQENKMPSTLRGSNKRGIDHWQVDYTHYEDKIILVWVE
TNSGLIYAERVKGETGQEFRVQTMKWYAMFAPKSLQSDNGPAFVAESTQLLMKYLGIEHT
TGIPWNPQSQALVERTHQTLKNTLEKLIPMFNAFESALAGTLITLNIKRKGGLGTSPMDI
FIFNKEQQRIQQQSKSKQEKIRFCYYRTRKRGHPGEWQGPTQVLWGGDGAIVVKDRGTDR
YLVIANKDVKFIPPPKEIQKE
>EIAV
MQKCSKKREARGSREAPETNFPDTTEESAQQICCTRDSSDSKSVPRSERNKKGIQCQGEG
SSRGSQPGQFVGVTYNLEKRPTTIVLINDTPLNVLLDTGADTSVLTTAHYNRLKYRGRKY
QGTGIIGVGGNVETFSTPVTIKKKGRHIKTRMLVADIPVTILGRDILQDLGAKLVLAQLS
KEIKFRKIELKEGTMGPKIPQWPLTKEKLEGAKEIVQRLLSEGKISEASDNNPYNSPIFV
IKKRSGKWRLLQDLRELNKTVQVGTEISRGLPHPGGLIKCKHMTVLDIGDAYFTIPLDPE
FRPYTAFTIPSINHQEPDKRYVWKCLPQGFVLSPYIYQKTLQEILQPFRERYPEVQLYQY
MDDLFMGSNGSKKQHKELIIELRAILLEKGFETPDDKLQEVPPYSWLGYQLCPENWKVQK
MQLDMVKNPTLNDVQKLMGNITWMSSGIPGLTVKHIAATTKGCLELNQKVIWTEEAQKEL
EENNEKIKNAQGLQYYNPEEEMLCEVEITKNYEATYVIKQSQGILWAGKKIMKANKGWST
VKNLMLLLQHVATESITRVGKCPTFKVPFTKEQVMWEMQKGWYYSWLPEIVYTHQVVHDD
WRMKLVEEPTSGITIYTDGGKQNGEGIAAYVTSNGRTKQKRLGPVTHQVAERMAIQMALE
DTRDKQVNIVTDSYYCWKNITEGLGLEGPQSPWWPIIQNIREKEIVYFAWVPGHKGIYGN
QLADEAAKIKEEIMLAYQGTQIKEKRDEDAGFDLCVPYDIMIPVSDTKIIPTDVKIQVPP
NSFGWVTGKSSMAKQGLLINGGIIDEGYTGEIQVICTNIGKSNIKLIEGQKFAQLIILQH
HSNSRQPWDENKISQRGDKGFGSTGVFWVENIQEAQDEHENWHTSPKILARNYKIPLTVA
KQITQECPHCTKQGSGPAGCVMRSPNHWQADCTHLDNKIILTFVESNSGYIHATLLSKEN
ALCTSLAILEWARLFSPKSLHTDNGTNFVAEPVVNLLKFLKIAHTTGIPYHPESQGIVER
ANRTLKEKIQSHRDNTQTLEAALQLALITCNKGRESMGGQTPWEVFITNQAQVIHEKLLL
QQAQSSKKFCFYKIPGEHDWKGPTRVLWKGDGAVVVNDEGKGIIAVPLTRTKLLIKPNZV
LLQEARPNYHCQLCFLRSLGIDYLDASLRKKNKQRLKAIQQGRQPQYLLZGLIYGSIWZR
GNMVSIAFYGGIPGGISTPITQQSEKSKCEENTMFQPYCYNNDSKNSMAESKEARDQEMN
LKEESKEEKRRNDWWKIGMFLLCLAGTTGGILWWYEGLPQQHYIGLVAIGGRLNGSGQSN
AIECWGSFPGCRPFQNYFSYETNRSMHMDNNTATLLEAYHREITFIYKSSCTDSDHCQEY
QCKKVNLNSSDSSNSVRVEDVMNTAEYWGFKWLECNQTENFKTILVPENEMVNINDTDTW
IPKGCNETWARVKRCPIDILYGIHPIRLCVQPPFFLVQEKGIADTSRIGNCGPTIFLGVL
EDNKGVVRGDYTACNVRRLNINRKDYTGIYQVPIFYTCTFTNITSCNNEPIIX
>HTLV-1
AKRPPVILPIQAPAVLGLEHLPRPPEISQFPLNPERLQALQHLVRKALEAGHIEPYTGPG
NNPVFPVKKANGTWRFIHDLRATNSLTIDLSSSSPGPPDLSSLPTTLAHLQTIDLKDAFF
QIPLPKQFQPYFAFTVPQQCNYGPGTRYAWRVLPQGFKNSPTLFEMQLAHILQPIRQAFP
QCTILQYMDDILLASPSHADLQLLSEATMASLISHGLPVSENKTQQTPGTIKFLGQIISP
NHLTYDAVPKVPIRSRWALPELQALLGEIQWVSKGTPTLRQPLHSLYCALQRHTDPRDQI
YLNPSQVQSLVQLRQALSQNCRSRLVQTLPLLGAIMLTLTGTTTVVFQSKQQWPLVWLHA
PLPHTSQCPWGQLLASAVLLLDKYTLQSYGLLCQTIHHNISTQTFNQFIQTSDHPSVPIL
LHHSHRFKNLGAQTGELWNTFLKTTAPLAPVKALMPVFTLSPVIINTAPCLFSDGSTSQA
AYILWDKHILSQRSFPLPPPHKSAQRAELLGLLHGLSSARSWRCLNIFLDSKYLYHYLRT
LALGTFQGRSSQAPFQALLPRLLSRKVVYLHHVRSHTNLPDPISRLNALTDALLITPVLQ
LSPADLHSFTHCGQTALTLQGATTTEASNILRSCHACRKNNPQHQMPQGHIRRGLLPNHI

WQGDITHFKYKNTLYRLHVWVDTFSGAISATQKRKETSSEAISSLLQAIAYLGKPSYINT
DNGPAYISQDFLNMCTSLAIRHTTHVPYNPTSSGLVERSNGILKTLLYKYFTDKPDLPMD
NALSIALWTINHLNVLTNCHKTRWQLHHSPRLQPIPETHSLSNKQTHWYYFKLPGLNSRQ
WKGPQEALQEAAGAALIPVSASSAQWIPWRLLKRAACPRPVGGPADPKEKDHQHHG
>HTLV-2
HRSRPYGYTPDTRARAGKAPRHPDPRRQWANQHPVQTPPNPPTHILALPKVPRYPFLLPL
RHPQQMDHHWKGRPTTMPGASIPPRRPQPPPIAANSHSKHHRPRTPSPTSPSGPISFKPE
RLQALNDLVSKALEAGHIEPYSGPGNNPVFPVKKPNGKWRFIHDLRATNAITTTLTSPSP
GPPDLTSLPTALPHLQTIDLTDAFFQIPLPKQYQPYFAFTIPQPCNYGPGTRYAWTVLPQ
GFKNSPTLFEQQLAAVLNPMRKMFPTSTIVQYMDDILLASPTNEELQQLSQLTLQALTTH
GLPISQEKTQQTPGQIRFLGQVISPNHITYESTPTIPIKSQWTLTELQVILGEIQWVSKG
TPILRKHLQSLYSALHGYRDPRACITLTPQQLHALHAIQQALQHNCRGRLNPALPLLGLI
SLSTSGTTSVIFQPKQNWPLAWLHTPHPPTSLCPWGHLLACTILTLDKYTLQHYGQLCQS
FHHNMSKQALCDFLRNSPHPSVGILIHHMGRFHNLGSQPSGPWKTLLHLPTLLQEPRLLR
PIFTLSPVVLDTAPCLFSDGSPQKAAYVLWDQTILQQDITPLPSHETHSAQKGELLALIC
GLRAAKPWPSLNIFLDSKYLIKYLHSLAIGAFLGTSAHQTLQAALPPLLQGKTIYLHHVR
SHTNLPDPISTFNEYTDSLILAPLVPLTPQGLHGLTHCNQRALVSFGATPREAKSLVQTC
HTCQTINSQHHMPRGYIRRGLLPNHIWQGDVTHYKYKKYKYCLHVWVDTFSGAVSVSCKK
KETSCETISAVLQAISLLGKPLHINTDNGPAFLSQEFQEFCTSYRIKHSTHIPYNPTSSG
LVERTNGVIKNLLNKYLLDCPNLPLDNAIHKALWTLNQLNVMNPSGKTRWQIHHSPPLPP
IPEASTPPKPPPKWFYYKLPGLTNQRWKGPLQSLQEAAGAALLSIDGSPRWIPWRFLKKA
ACPRPDASELAEHAATDHQHHG
>BLV
LERLQALQDLVHRSLEAGYISPWDGPGNNPVFPVRKPNGTWRFVHDLRATNALTKPIPAL
SPGPPDLTAIPTHLPHIICLDLKDAFFQIPVEDRFRSYFAFTLPTPGGLQPHRRFAWRVL
PQGFINSPALFERALQEPLRQVSAAFSQSLLVSYMDDILIASPTEEQRSQCYQALAARLR
DLGFQVASEKTRQTPSPVPFLGQMVHNQIVTYQSLPTLQISSPISLHQLQAVLGDLQWVS
RGTPTTRRPLQLLYSSLKGIDDPRAIIQLSPEQLQGIAELRQALSHNARSRYNEQEPLLA
YVHLTRAGSTLVLFQKGAQFPLAYFQTPLTDNQASPWGLLLLLGCQYLQTQALSSYAKPI
LKYYHNLPKTSLDNWIQSSEDPRVQELLQLWPQISSQGIQPPGPWKTLITRAEVFLTPQF
SPEPIPAALCLFSDGATGRGAYCLWKDHLLDFQAVPAPESAQKGELAGLLAGLAAAPPEP
LNIWVDSKYLYSLLRTLVLGAWLQPDPVPSYALLYKSLLRHPAIFVGHVRSHSSASHPIA
SLNNYVDQLLPLETPEQWHKLTHCNSRALSRWPNPRISAWDPRSPATLCETCQRLNPTGG
GKMRTIQRGWAPNHIWQADITHYKYKQFTYALHVFVDTYSGATHASAKRGLTTQMTIEGL
LEAIVHLGRPKKLNTDQGANYTSKTFVRFCQQFGISLSHHVPYNPTSSGLVERTNGLLKL
LLSKYHLDEPHLPMTQALSRALWTHNQINLLPILKTRWELHHSPPLAVISEGGETPKGSD
KLFLYKLPGQNNRRWLGPLPALVEASGGALLATNPPVWVPWRLLKAFKCPKNDGPEDAHN
RSSDG
>HSRV
MNTPVYPVPKPDGRWRMVLDYREVNKTIPLTAAQNQHSAGILATIVRQKYKTTLDLANGF
WAHPITPESYWLTAFTWQGKQYCWTRLPQGFLNSPALFTADVVDLLKEIPNVQVYVDDIY
LSHDDPKEHVQQLEKVFQILLQAGYVVSLKKSEIGQKTVEFLGFNITKEGRGLTDTFKTK
LLNITPPKDLKQLQSILGLLNFARNFIPNFAELVQPLYNLIASAKGKYIEWSEENTKQLN
MVIEALNTASNLEERLPEQRLVIKVNTSPSAGYVRYYNETGKKPIMYLNYVFSKAELKFS
MLEKLLTTMHKALIKAMDLAMGQEILVYSPIVSMTKIQKTPLPERKALPIRWITWMTYLE
DPRIQFHYDKTLPELKHIPDVYTSSQSPVKHPSQYEGVFYTDGSAIKSPDPTKSNNAGMG
IVHATYKPEYQVLNQWSIPLGNHTAQMAEIAAVEFACKKALKIPGPVLVITDSFYVAESA
NKELPYWKSNGFVNNKKKPLKHISKWKSIAECLSMKPDITIQHEKGISLQIPVFILKGNA
LADKLCGGMPLETRENZEESITGKEVKNLVTQILLLLIDVPGMRDTRFLNCPHSLLPLTS
NAGLLKHCLMAGKWSPKAEMIILAAERSEHZQRZLRMKLEKGGKVDIVTPSLTQEVTQMD
PSKSHRQZKCVSTLYNIISAZAIEAFTYSVAVSQSTKQZZCNHKEVVZNRLISLLVIZKI
IZDKVZGLRYEVCGSTRRVTRKSTVIGHNTSKVARGKVKZDRIFPZRRQLFIHZGZCFKE
YYSNRLIVLTMMEIVYKDSFZIVR
>HFV
MNPLQLLQPLPAEIKGTKLLAHWDSGATITCIPESFLEDEQPIKKTLIKTIHGEKQQNVY
YVTFKVKGRKVEAEVIASPYEYILLSPTDVPWLTQQPLQLTILVPLQEYQEKILSKTALP
EDQKQQLKTLFVKYDNLWQHWENQVGHRKIRPHNIATGDYPPRPQKQYPINPKAKPSIQI
VIDDLLKQGVLTPQNSTMNTPVYPVPKPDGRWRMVLDYREVNKTIPLTAAQNQHSAGILA
TIVRQKYKTTLDLANGFWAHPITPESYWLTAFTWQGKQYCWTRLPQGFLNSPALFTADVV
DLLKEIPNVQVYVDDIYLSHDDPKEHVQQLEKVFQILLQAGYVVSLKKSEIGQKTVEFLG
FNITKEGRGLTDTFKTKLLNITPPKDLKQLQSILGLLNFARNFIPNFAELVQPLYNLIAS

AKGKYIEWSEENTKQLNMVIEALNTASNLEERLPEQRLVIKVNTSPSAGYVRYYNETGKK
PIMYLNYVFSKAELKFSMLEKLLTTMHKALIKAMDLAMGQEILVYSPIVSMTKIQKTPLP
ERKALPIRWITWMTYLEDPRIQFHYDKTLPELKHIPDVYTSSQSPVKHPSQYEGVFYTDG
SAIKSPDPTKSNNAGMGIVHATYKPEYQVLNQWSIPLGNHTAQMAEIAAVEFACKKALKI
PGPVLVITDSFYVAESANKELPYWKSNGFVNNKKKPLKHISKWKSIAECLSMKPDITIQH
EKGISLQIPVFILKGNALADKLATQGSYVVNCNTKKPNLDAELDQLLQGHYIKGYPKQYT
YFLEDGKVKVSRPEGVKIIPPQSDRQKIVLQAHNLAHTGREATLLKIANLYWWPNMRKDV
VKQLGRCQQCLITNASNKASGPILRPDRPQKPFDKFFIDYIGPLPPSQGYLYVLVVVDGM
TGFTWLYPTKAPSTSATVKSLNVLTSIAIPKVIHSDQGAAFTSSTFAEWAKERGIHLEFS
TPYHPQSGSKVERKNSDIKRLLTKLLVGRPTKWYDLLPVVQLALNNTYSPVLKYTPHQLL
FGIDSNTPFANQDTLDLTREEELSLLQEIRTSLYHPSTPPASSRSWSPVVGQLVQERVAR
PASLRPRWHKPSTVLKVLNPRTVVILDHLGNNRTVSIDNLKPTSHQNGTTNDTATMDHLE
KNE
>HERVL66
QEEARGICHLYTNSWSVANGLTTWMPQWQRNKWLIGNKEVWGKQYWEDISILAHTTIITV
FHVDAHASLLSLDRLFNQQADQQAKISTITANSDPEEADWAAITQWVHHQCGHLGVQGTM
AWGVQRRISLPQDAVQTILSQCTTCQQLKTKPIPQRAMGHIHRGKMPGQICQMDYICPLP
LSKGCQYICTAVDTYSGLLVACAYANANQINTIKTLNILILYYGVPIQIQTDNGSHFKGE
AVQTFAAQDGIEWIFHIPYHLQAAGLTERMNELLNKQLKVLGQGKLEKWKDHLFDTLQNL
NNWRLTTSETPVSQMLTPHLQIAKCASVVQPLSLKFWKIHPEAILPWKSTREATGLDLHS
FKSGIIPAQSTYMVASGLG
>SnRV
MASNKWFVYSDEPTKVILKRDKSKEKDETKKKKIKTEQNSDAAYETPGTAPVQKPLLETT
PEAELEKVLKGLEEWGYKALEKKRDPELWNPNQEGLDEYLFRGWVQGGLADSKKALEKNM
EKFVPLFVVTMSQAVPYWRQTMQARNQNGKKQKNRIAELEKEVADLTSAGRGADQVIAGM
DKELKKTAEKYQAKLEELEEQLAAMTVEKEELESQVEGLKESLVEAETKKVSLMEVLTMP
TRSKGPKKRGPDLKQIRSLHVMADSLGMDSDGIDWDWLARQAWDYEGDEDPHVKEAEEEA
WRERQTSQPSQPSQLRPFVAAGNGHREDQWRPLTVTELPAAVTAVGGAWDPTRETGSARW
KKIVKAAEAIGWGTGDVCQVVTAMSPSWADVPPEIRNRVATEKEIKAWLMKQGPGGGQGL
LEFTKLRQGPTENPSNYLEKALELYLDSQPGDRDGNKDDPAFLQQATQGLLPWLKKAVIL
GGKNTSWQEMTSFCQRLWLVRDQFADKTGVSKARPIVRNEGPRPQQGHSKIVFGGNCRNC
GKAGHMARDCWAKGGGQEGKGPRQNTTWKPKSGAIASAPPAESPYADCAKQLADIEKRLK
DLTTAGGGPKGPNPFHKPZGVAALAAPLCSLKGRPHVSVEIEGHKIECLVDTGAEVSLTS
LQLQAQRFEQVVGLGGKPVRVGIADHVDTTVGQVKGKGCWRISQELAENILGNDLLRSLG
LIVDQCNGVLWQASEGLGPDNWMAAETLRIYSIKSPGHYNLPELLATKDEQLADILWNNV
EAFATHRNDCGNLQGMTASFTADHPKMIKQYPVPDASHASIKETVEALLEQGVLRKCNST
VNSAIWPVGKPDGSWRLTIDYRPLNSAVSCPYPTVASTPELFAKLEKKYQVYSSLDISNG
FWSIRLEEECQYLFAFTFDTQQYTWTRLPQGFHASPGIFHQALYNGLASCKTAIESQGCK
LLQYVDDILLMSEDRDHHLRSLAILLQGLKDLGVKINPKKSHFCKDQVQYLGVNVGADTR
SLIDARSQLIRTLDIPLTVQGLRSALGLFNFCRAWIPEFSRKTQSLYDMLKGDCKSTDKL
KWTEDNLNKFKLLKDEVASACVLGLPDPTLPFRHLIGIRQGHFLCSLVQKDDNGMWHVLG
FYSRKMTPVESNLGICEQYAECAAWAISACNLVSGFGRKIIVTSHSPVKFILQTTPNVSN
QRLARWHRILTQEDITIETDASIQGWFVPEPYEGEQHQCRPHDDTITWRVSTRAMPTGEK
WWIDGSRYWDHDKGGYVTGWAALREDKKNQLGGALEGHVSAQVAELVALREALRLQRPLT
LYTDSTYVLGICTKYLAVWKRRGMVNADGSQISNQNILQEIWQLIEHDSTQTLGIVKVKA
HTQRKCSTHEQQLNNDVDQPAKQYAKEEPNMSVIAPLQVYPLWIGLVPCKEPKLWENIQH
HITKVDLPDFQKQLAEIMPQQDISHCTLAYFDKPSPEATKYHDKIKPYLGKGQQLTLCDT
YIGKEGAAILGQLRPDMQALHQAEGEVHVSLGTRAGHCPQELGTMLTNLLKSTQERIWQD
PPVFKLHDETGKVQGYVIKTTLGMKTWIMNDHLVTQSEQTEGRAKLSSTEGYALAQQYHH
LYGHPSEESLRKVLTKRFVWEDMGQHCKEITNTCLTCAKYKVLRAGPPMGVGRSAEGPCQ
KLQVDHVGPLGPGTHGYRYLTTMVDVYTGWFWAKPCRGPTTGATIAALEEHISIWGVPYS
IQSDNGTAFTSKAMQEWANTYGIEWKVGAIYHPQSQGKVERKHRLLKDRLKRATHEGKNW
VQALPSILLFINSMHPRDQFSAYELMTGRVPHLGGYHPHPLETAKEEEVRIFLRATHDCM
QNKKWEEQLEKVTKAEAHSHWTQRSPNLEPGCIVLVRKFTGDAFSPKWEGPYVITETTKY
AAKVQAMSDKVTQHSGWIHRTHLVLFPSQNKRWADPGNPGNQPDEDCTGKKGTVSTADMS
PTTSTTRDRGINMGTEQTHRRRSPRFQRGSDSPSGGQZ
>gg01-chr4-77338201
FRITAIAWTFPLPLTWTTNNPVVVKQWPLKRESLLQAHQLVQEQYAQGHLKLSTSPWNTP
IFVIQKKSGKYRLLHDLRAVNDQMEPMGALQPGLPNPAMLPEDWPILIIDLKDCFFTIAL
HPQDTKRFAFTLPAINRGEPDKRFEWTVLPQGMRNSPTICQLYVDAALQPLRKEMPNTII
YHYMDDILFAQQDPFTEQQIERVKTVLAEFSLVVAPEKVQRSAPWKYLGWQITGKQVXXX

XXXXXXXXXXXXXXXXXXXXXXXXXXXXXXXXXXXXXXSRLDPNLPLDLMIWNNRTHLLG
AITQFRKKMGERVLEWLSPPLQKRKTITTKIENLAAVIRKGRVRILEVSGREPETIYLPM
EKATLDWYLLNSPDLAEALLASGARVKSGPLVPRALSGLAHSGSLLVGRSLSPLLKLDRI
RDPVLHLNLLLKPHQTLLHHLLPQQKVTITSVNQGLEILTTLEGFNNLGDQPFACRGCGR
VWGCGAWVLIWCGGCGKQVWVHQSQLQPSGAVAVRTVGGGGILGEPEQFTGESFWNALAL
YESTEQRALARIRWEFKGLVGRAYALLQSRILKAHCGDITSAPLLTLILLQISLIKSLWM
PAQPKQNIWITLANKMGQDHICLALSSPNNPFTTCLVGVPLDVLLVPRQLSLVTVLPQMI
MQINGTSTCLFPVAGIEPQELELLGSLKADFCLFFNWTSTQRGNNGTVCVNSSLSVYNNQ
TAWCNYTTPNISVSSNSPIKLPEGVFLICGDRAWGGVPSHLEGGPCTLGRLTLLNPNTS
>HERVL40
SNXDSIESXEEERESVPZVNTNTYAAEVVRDXXSXXNSLVXPVXKXNXSXRXTVXCXXXD
SVXXPVAPAAXDIVTXXESTTRXXXXWCAVLNIXNXXFXIPEASEDQXXFASVARPXHVF
XXLXQXYLNSPAICHXWMGQDLARXPLPSDVQSFHYIDXVLLVGKSEASXSTALTAVITL
LXXMADKLQKHSGTCLPSKVSWXYVGRFTXXNPSGSQEKAVSSGPXHQRGTLLDSLXIGD
GLCLAWAFYLVLYISXPTNQYPLSGPNQKAALEXVQXAVXRSLPLGPHNPNDTFELQXSV
TDDFANWSLWQGEATSTWGTPWGFGLTFLLIRLPDTPLLKSSYZLATELLSKLNTXPTEA
LTLQPDIPTLGWVNLDSMTNKEKGSTSLACQIEMAIFKNAAXXAPAASXLYMKKWQLSFG
GNFIPHSAARSKAAGSIGPSIHRGXPKCWAWFTDGLAKLKPNGVHWAAAAVQPQXQXXKT
ENRQGRSAQWXEFKAILITWTILPLIXXVIFLLTYTVANGLAVWSATWKTTDWQIKDTPL
WGRKLWKQIVAXDZTIWVTHVDAXVGPFSDETKWNQAAIEPAAQIATXAAWIHHPTGHGN
TSTIXDWAQSKGPYVSDAEATTACQTCDSCQKLPVXSAVREATLHRALPLSTSGXLLHRT
FDXLLGLPMASPLLTHFSXYGVAVPVQSADSSHTIVPETXLCXVFSFPDYLQSDNGAPFI
TKATQQWLSQGIQWTFHAPYYPRASGIVERWNGLLITDSKRFLTXSPSPPPGPHTLVDNL
VTXYGCPRKGSSPFGRFLSNDQYKRRVREV
>Gypsy
NKLWATQHRRRKTHPTLPYQLKFSGTRLRNPTGYINPTTHATSLPYITLNLQQKFPLSFL
IDTGSNNSFIDPESANQLECTILPTSTSITTALNSFKIEEKAIFPMPPEFKTEGQITLLK
FKFHSYFNGLIGMDLLSHLEAKVDLVNLQLVTSKSTLPIFLYTNQASKIFNIPAYSKVIL
PLPVKTNHGEFYCCTTQLNNELSLSEGLYKSNNNIANVEISNQSDSDKLLYLEYPLETIP
YNKNDHIELFNISATPLNNDTPQAPLHILTEHLNPEEKTALTTLCKQFRDIFYNPETPLT
FTNKITHSIPTIDNTPIHTKSYRYPFVHKTEVKKQIESMLDQQIIRSSHSPWSAPVWVVP
KKLDGTGNRKWRLVIDYRKLNDKTISDRYPIPNINDILDSIGKAKYFSTLDLTSGFHQIE
MNPKDIAKTAFTVEGGHYEFTRMPFGLKNAPATFQRVMDSVLGDLNGTICLFYLDDIIIF
SPSLQKHLLDIKMVFEKLRAANFKLQPSKSEFLRKEIEFLGHIVTQDGVKPNPNKISAIK
KFPCPTNRRAIKSFLGLLGYYRKFIRDFARITKPMTKQLKGKRQVTTDKDFVDAFEQCKT
LLSNDPILIHPDFEKPFILTTDASNFALGAVLSQGSLQNDRPVCFASRTLSDTEVNYSTI
EKEMLAIIWAVKYFRPYIYGVKFTIVTDHKPLIWLMNFKEPNSKIIRWRLQLMEYNFEII
HKKGSQNVIADALSRADPNLNYNETLTVKPCPTSEKPINEFNTQLILEIDTNTSYQTTTP
FKQKIRKKYSQPCFDFDNIVKILKGTLKPNRICAFLADDNNSALIEKAFSTYFAHKKHFK
IIRCKSLLHEIVGNPEQNKFIQEYHTNSNHRGIDETFLHLKRETYFPNMKNKISELIRNC
ETCLKLKYDRQPQNIVFETPETPSKPLDIIHIDIYTINNNFNLTIIDKFSKFAAVYPIPN
RNGINCIKAIKNFFSQFGLPKKLIHDQGVEFCNDIFRKFCSQYNILLHVTSFQQSSSNSP
VERLHSSLTEIYRIILDTRKKHKLPTDHEEIMSETVITYNNAIHSTTKHTPFELFNGRTH
LFEKTIIPNNEHDYLNKLNTFQDKLYSEIKEKLSTNTQQRIEKLNTSRVEPTTVQPNSTI
FRKENRRNKLTPRFSLHRTAKDKGKTLVTTRNQKIHKSKIRKISKPPNDLSLSTCIPDLA
MGHTNLSSSTTSIAPTSZQKCRZGKHSZZETIKKLATZSICPNTPTVLKNYTTPSIPZDK
MKHSPILYQYZMLNWPKLKVKZTHZHPFHATKEVLLTGZVVZSKSSPATWTPMMQRIZKQ
KLTTZKASPPLSQITSKYRTRSMMKFNYGSKTZQDTLTMNRIZLKTSSKTLKIQFTQKYT
TTKKKZRNYNIZIGLTTIZIYZLATZATLZKVHCLPKLMLSQNSSZTRQKZPKSNKFLKH
KTTQZNPSNTFITSZKZTHSITKTKZFLVSKFLFFZVVTTKWQDZFHFQZIPHNLZZHLS
TZYIITKVTACFQLCINVLZZKNNSSAKSTPSIILKIILAWDTLSKIRPATATX
>HERVL74
KYPMVGIDALTMSNIKXKLSLWHLQIGLTKWDPMDPPVKIVNMAQCKLKQGLQELKLIIZ
DLXSEGVIIPTASPFNSPILPVLKPGKNEWTSRWITATLMPWSHPLRPPYPISNIIEITD
SIQSVTGKYFAXIDLANMFCSVPISTASQLQFAFTSEGTQYTFTRLLMGYLNSLSSRTIF
AGKILTASXFPGAQVZHYIDDILLQGDSFDTLIKDIQVQHLLXLFVLEATYSPFTILLXL
TDXAIYKAHLNGLLNKRLWNLSKLKSTGTPVSALRDSFTVEALATSSHASWSLWTTYDAI
SCPGLLMQETALLGLMLTPLERQLLATYWALLEIEALADPEPVTLHTQLPIMPWVMEAAP
HKLSTATEASLXQDSQTWASGIFHLQEGVASSVLSPLPDHVLEEVTPPPDPLATWXAPWD
QLSEQQWEFIDYMDGIATIISDGAQWNVAAFHPLTRMSLIKDGTQGSAQLAKLQAVILAL
DALATNGLIXTFLQTLAIAQELSPRVKNSGNLLPHRYKIZIKITHVSAHTKATIQGLTRT

```
SLSPSEFQTLLIVTKAHFASQNTQCWALEKGIQWNFHLPXRPQIAGXIERIMVSSNNSFX
NXXPTNRPLNGSLSCQALISLNSRPFGXLTPYNXKKRKPFHHPFLYLKRDMXHLHXYEDS
YICGPNNVSSYLYKFSILPPSHKGWRPDRRHILSWDPLTIDCPIGQXAAHLIWDPSEPKL
PPPKNLPNIWVIRSGZLXRALPLIPSRKLKLVLQGXSHRQCGLPRRETENPTZZXKIXFG
DNNGINTISHNDKSRQHLLGNNARQGXRKSTSNN
>gg01-chrU-52190725
PDTVGRLDITLFEVTIKNPEIPVRIKQYPLSQEGRQGLKPEIERLLKRGLLEPCLSPFNT
PILPVRKPDGSYRLVHDLREINKRTVTRFPVVANPHTLLSQLSPENQWYSVIDLKDAFWA
CPLKEECRDYFAFKWEDPETHRKQQLRWTVLPQGFTESPNLFGRALEQILRGYNLGKEIA
LIQYVDDLLLAGDTLESVREASVKTKLLGLQGLKIPNQSYNYRIHXXXXXXXXXXXXXXX
XXXXXXXXXXXXXXXXXXXXXXXXXXXXXXXXXXXXXXXXXXXXXXXXXXXXXXXXXXXX
XXXXXXXXXXXXXXXXXXXXXXXXXXXXXXXXXXXXXXXXXXXXXXXXXXXXXXXXXXXX
XXXXXXXXXXXXXXXXXXXXXXXXXXXXXXXXXXXXXXXXXXXXXXXXXXXXXXXXXXXX
XXXXXXXXXXXXXXXXXXXXXXXXXXXXXXXXXXXXXXXXXXXXXXXXXXXXXXXXXXXX
XXXXXXXXXXXXXXXXXXXXXXXXXXXXXXXXXXXXXXXXXXXXXXXXXXXXXXXXXXXX
XXXXXXXXXXXXXXXXXXGIDVWVKIMYSLPGTGAECGLGGLAGSFMATEEFPQPLFTLQ
RKTQLNLTRDCYNLLSCSFDIIAVFFFSLTSSTSHPLGKZGDIVLFSTLILCLGCAITSS
SWNSFGKLCGSLLSQHIMVSLYLNRRRQQDLRRVLRSDLCDFRINSVIFTTZDPTCFFLZ
CSGIWMAKHITLDKNHTZALEKTGLTAKFSIWSLFSSCLMFLDLRKLVTLRSRCELSELA
LFAVIRRKYVKMRAENFPZEIPNKTFDCMHFALFGKKEKEKKKKGKATYTSQMLVTFLVT
SLNAPRKKSETMELSTVKISRKKKKMHKSZVKCLKTQNSDSVKLHDTWAYFKLISLIQLQ
SLPRGWWHLVWPCTSAHISANTEFAEEDLHTFSQEESQRSYLPRRDQSDLYVTGLGDIIL
ITLPGLAFPILKGTQSTKFNSPKKRWKQSXGKKKRKEK
>gg01-chr7-7163462
PVSKGEHKQMLLPGSLNMDHLLTDAQSIWLNGGRYTEAHNPPHPVGSRVCMRNFLQEVEL
KLKGFSVKKLRQZLGRMLTYRVDTYRVDNFFNRLLPQYRWTVLPQGFTESPILFGKVLEK
ILESFQAQETVILLKYVDHLLLSGTEKSKVKETTNRHPNFLGKYGLRVPRNKSQYVVKEV
RYLGCIFYEGKWRTNSDRVRELCDSLSWFWRGSLGAKLGSYNISTMAISLKNWIPFSIAS
HVSITGCRISLZGYLSLTAINIQSQRVAAPSKLLLIPLSIDLSLAMSPIWRASLPSRDKL
LAPPSQQRSNQVTRFSFGRRVNSFPELWISVILRGZRIVIEISSSSLSSPCLLVFAFAFL
GSEEVPSPGSSSTSSSTSYSFFCLFVIDVRTPASISCLLQGLLTLLLVYAVTHQIZLEDF
PLWLAPQLGNSSLQNSTIQSMVGTSQAPNKLYLLIYLSRTIPDSNAGSISRDPTDVQEZN
PMTLRVPTLLRFFLNLSVFPASQHSLVLEETSSLARMERNTFKDIDNMRTSMTTSSVFKK
RDKLRGKPGNRFESLKIQIIQHCIKAPGRCLHQCPYLVVYKHNSIARNYDVNYRPVTGKE
HIAFNPLYSAPTIKSKLIAGNCZREGGEKKKQKETTRYSVWSLVDCPGYNLIYEGHPTPV
MPCSHAVSGLKKFIFPTNVKSFRLPAFSTKNLSRSYLDKSVPVTGGQZAHGPPGFPKIKR
SSGNNLRRRTYFTNYDVGMQDDTI
```
